# Targeted interventions to improve the social and economic circumstances of people with mental ill-health from marginalised communities: a systematic review

**DOI:** 10.1017/S0033291725101128

**Published:** 2025-07-28

**Authors:** Helen Baldwin, Anna Greenburgh, Hannah Weir, Zara Asif, Dionne Laporte, Mark Bertram, Achille Crawford, Gabrielle Duberry, Shoshana Lauter, Brynmor Lloyd-Evans, Cassandra Lovelock, Jayati Das-Munshi, Craig Morgan

**Affiliations:** 1 ESRC Centre for Society and Mental Health (CSMH), King’s College London, London, UK; 2 Population Health Improvement United Kingdom (PHI-UK), London, UK; 3 Lambeth Vocational Services, South London and Maudsley (SLaM) NHS Foundation Trust, London, UK; 4 Independent Researcher; 5 Culturally Appropriate Peer Support and Advocacy Service (CAPSA), Black Thrive Global, London, UK; 6 Care Policy and Evaluation Centre (CPEC), London School of Economics and Political Science (LSE), London, UK; 7 Division of Psychiatry, University College London (UCL), London, UK; 8 ESRC Centre for Society and Mental Health (CSMH) Lived Experience Advisory Board (LEAB), King’s College London, London, UK; 9 South London and Maudsley NHS Foundation Trust, London, UK; 10 Department of Psychological Medicine, Institute of Psychiatry, Psychology & Neuroscience (IoPPN), London, UK

**Keywords:** common mental disorders, economic interventions, severe mental illness, social interventions, targeted intervention

## Abstract

People who experience mental ill-health are typically more disadvantaged across a range of social and economic domains compared with the general population. This disadvantage is further heightened for people from marginalised communities. Social and economic adversities can limit both the access to, and effectiveness of, interventions for mental ill-health; however, these challenges are often overlooked by mental health services. Therefore, adequate support for social needs is urgently required, particularly for those from marginalised and vulnerable groups. We conducted a PRISMA-compliant systematic review of three academic databases to identify social and/or economic interventions which were adapted or developed bespoke for people from marginalised or minoritised communities living with mental ill-health. All records were screened blind by two reviewers; quality appraisal was conducted with the Kmet tool. Seventy-eight papers were included, deriving mostly from high-income countries. The identified interventions targeted nine sociodemographic or socioeconomic groups including: people experiencing homelessness or unstable housing (*n* = 50), people with an offending history (*n* = 9), mothers (*n* = 6), people experiencing economic disadvantage (*n* = 3), older adults (*n* = 3), caregivers (*n* = 2), minority ethnic groups (*n* = 2), women with experience of intimate partner violence (*n* = 1), and people with comorbid intellectual disabilities (*n* = 1). All identified interventions demonstrated feasibility, acceptability, or effectiveness on at least one social and/or economic outcome measure, suggesting that targeted intervention can help to address social and economic needs and reduce systemic inequalities in mental health care. However, the evidence base is still sparse, and further replication is warranted to inform commissioners and policy makers.

## Introduction

Currently, social and economic needs are typically underassessed and poorly addressed by mental health services (Boardman, Killaspy, & Mezey, 2022; Lambri, Chakraborty, Leavey, & King, 2012), despite pronounced social and economic need in people with mental ill-health (Jones et al., 2020; Nuyen et al., 2020; Pevalin, Reeves, Baker, & Bentley, 2017; Phillips et al., 2023; Sareen, Afifi, McMillan, & Asmundson, 2011; Stain et al., 2012; Topor et al., 2019). A range of effective interventions have been developed to address these needs (Barnett et al., 2022; Killaspy et al., 2022).

The social and economic adversities experienced by people with mental ill-health are further pronounced among those from marginalised groups (Giebel et al., 2020) who may experience multiple, intersecting disadvantages resulting from their identity. This may include minority ethnic groups (Morgan et al., 2008, 2017), people living in unstable housing or facing homelessness (Queen, Lowrie, Richardson, & Williamson, 2017; Quirouette, 2016), and people experiencing economic hardship (Boardman et al., 2022). Marginalised groups also experience reduced access to (Schlief et al., 2023), and poorer outcomes from (Barnett et al., 2023), existing mental health interventions as a result of these unmet needs. As such, targeted intervention that addresses the specific social and economic needs of marginalised communities may work toward addressing these inequalities and achieving equity of care.

Indeed, such approaches have offered promising impacts for some minoritised groups with mental ill-health in the receipt of targeted psychological intervention (Arundell et al., 2021; Ellis, Draheim, & Anderson, 2022). However, there is currently no systematic evidence synthesis reviewing targeted interventions addressing social and economic needs of marginalised groups living with mental ill-health. As such, it is not clear which interventions currently exist and for which communities. This topic is even more pressing given the disproportionately harmful impacts of the recent COVID-19 pandemic and economic crises on marginalised groups (Camara et al., 2023; Das-Munshi et al., 2023; England et al., 2024; Siimsen et al., 2023; Thomeer, Moody, & Yahirun, 2023).

Therefore, we aimed to: (*i*) review existing evidence to identify interventions addressing social and/or economic needs that have either been adapted or developed bespoke for people from marginalised or minoritised sociodemographic or socioeconomic groups with mental ill-health and (*ii)* narratively examine the types of interventions studied and their respective outcomes.

## Methods

We conducted a two-stage systematic review in line with a predefined protocol. This review was conducted as part of a broader research program which sought to identify interventions designed to address social and/or economic needs in people living with mental ill-health (Greenburgh et al., 2025). Here, we review studies that reported targeted interventions to directly support the social and/or economic needs of marginalised groups experiencing mental ill-health. See Supplementary Materials I for the full inclusion criteria.

We first utilised bibliography searches of two recent reviews on this topic (Barnett et al., 2022; Killaspy et al., 2022) to avoid duplication of efforts. Together, these two reviews represent rigorous, broad, and relatively recent narratives on the subject area of social interventions for people living with mental ill-health. However, this current review represents a related but distinct topic of targeted intervention. Furthermore, the global context has shifted since the searches for these reviews were conducted, given the COVID-19 pandemic and worsening economic crises. As such, we then replicated the original search strategies from both reviews to identify recent literature (January 2020–February 2024). Searches were conducted in MEDLINE (Supplementary Materials II), PsycINFO, Web of Science (SciELO database), and the Cochrane Central Register of Controlled Trials (Supplementary Materials III). All records were double-blind-screened by two reviewers. Data extraction was conducted within a fit-for-purpose extraction form (Supplementary Materials I) by one researcher and checked by a second independent researcher. Quality appraisal was conducted using the Kmet quality assessment checklist (Kmet, Cook, & Lee, 2004) by one researcher, with a random sample (10% derived from a random sequence generator) conducted by two reviewers. Conflicts in decisions were discussed with the wider review team until a consensus was reached.

Data synthesis was conducted via a narrative synthesis of the identified interventions, whereby we provided a summary of the content and results for each of the included studies. We did not plan to conduct meta-analyses due to the expected heterogeneity of evidence.

## Results

**Figure 1.**
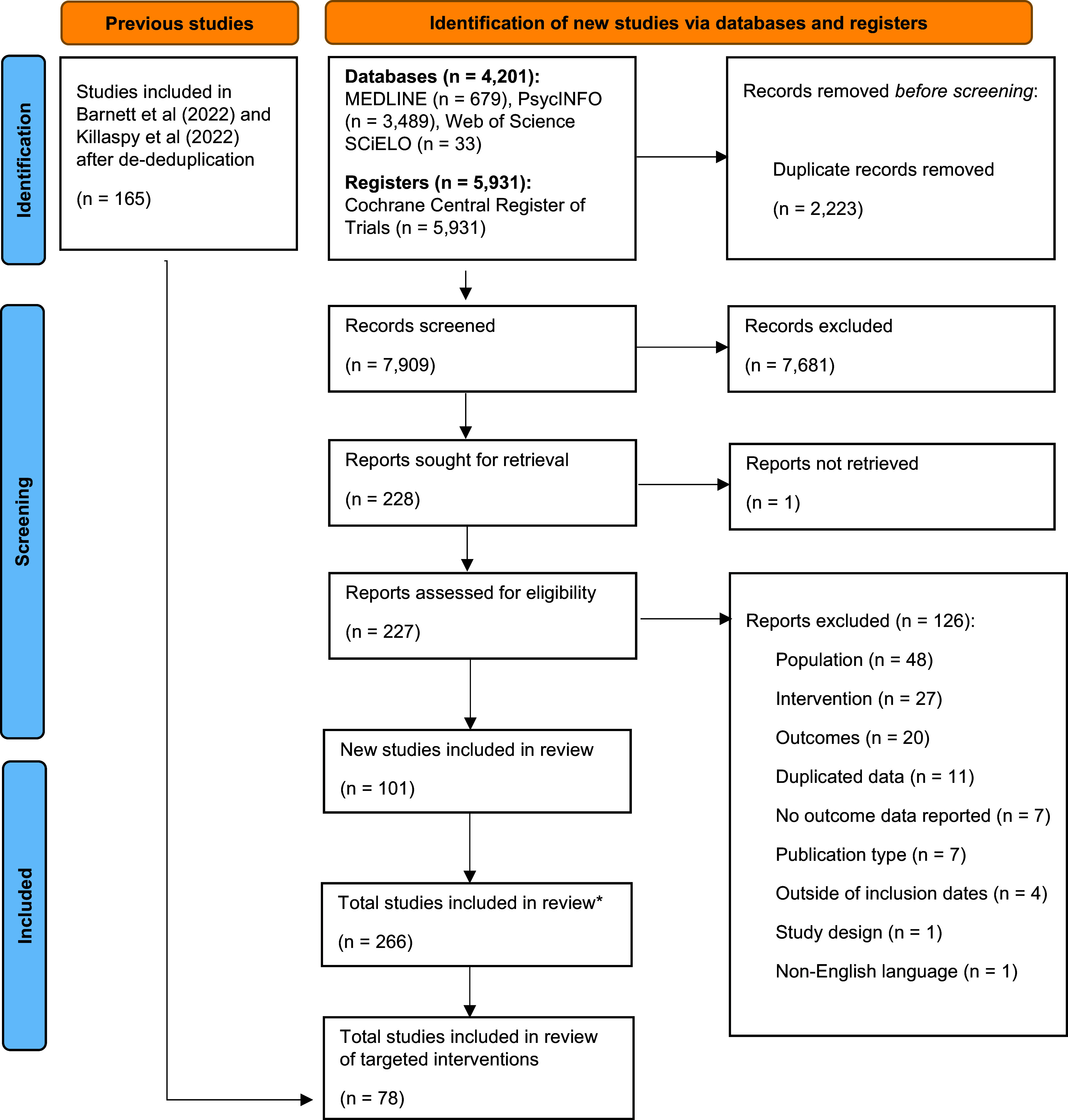
A PRISMA diagram demonstrating the flow of studies in the review. *Please see Greenburgh et al. (2025) for details regarding the broader systematic review of social and/or economic interventions for people living with mental ill-health.

Seventy-eight studies were included that reported on interventions adapted or developed bespoke for a specific sociodemographic or socioeconomic group (Figure 1). These groups included: people experiencing or at risk of homelessness, people with an offending history, mothers, caregivers, minoritised ethnic groups, older adults, people experiencing economic disadvantage, women with experience of intimate partner violence, and people with intellectual disabilities. The studies were conducted across 16 countries: USA (*n* = 36), Canada (*n* = 18), UK (*n* = 5), France (*n* = 4), the Netherlands (*n* = 2), Spain (*n* = 2), Australia (*n* = 2), Switzerland (*n* = 2), Portugal (*n* = 1), Norway (*n* = 1), Vietnam (*n* = 1), Pakistan (*n* = 1), Germany (*n* = 1), Finland (*n* = 1), India (*n* = 1), and Bangladesh (*n* = 1). Kmet quality scores ranged from 81–100 (quantitative) and 40–100 (qualitative). Summaries of the evidence from randomised (Table 1) and nonrandomised studies (Table 2) are described later. Key intervention terms are summarised in a glossary (Supplementary Materials IV).

**Table 1.** A summary of the characteristics of the included randomised controlled trials

Author, date	Location	Study design	Sample size	CMD/ SMI	Life domain(s)	Intervention	Target group	Quality score^a^	Social and/or economic outcomes
People experiencing homelessness or unstable housing									
Adamus et al. (2022)_u_	Switzerland	RCT/ Observational cohort study	141	SMI	Housing	Independent supported housing (ISH) compared with housing as usual	People experiencing unstable housing	Kmet Quant score = 92	After one year, ISH was noninferior to the control condition regarding social inclusion outcomes in terms of mean differences (95% CI) on the German version of the Social Functioning Scale, in both the RCT component (6.28 [−0.08 to 13.35]) and the observational component of the study (2.24 [−2.30 to 6.77]).
Aubry et al. (2016)_b_	Canada	RCT	950	SMI	Housing	Housing first with assertive community treatment compared with treatment as usual	People experiencing homelessness	High risk of bias	Participants allocated to HF spent more time in stable housing than those in TAU (71% versus 29%, adjusted absolute difference [AAD] = 42%, *p* < .01). Compared with TAU, HF participants who entered housing also did so more quickly (73 versus 220 days, AAD = 146.4, *p* < .001), and had longer housing tenures at 24-months (281 versus 115 days, AAD = 161.8, *p* < .01). HF participants were also assessed as having better community functioning (ASMD = .18, *p* < .01) over the two-year period, and showed significantly greater gains in community functioning within the first 12-months; although this attenuated by 24-months.
Aubry et al. (2019)_k_	Canada	RCT	201	SMI	Housing	Housing first with assertive community treatment compared with treatment as usual	People experiencing homelessness	Kmet Quant score = 92	In intent-to-treat analyses, compared with participants allocated to TAU, HF participants who entered housing did so more quickly (23.30 versus 88.25 days, *d* = 1.02, 95% CI: 0.50–1.53, *p* < .001), spent a greater proportion of time stably housed (*Z* = 5.30, *p* < .001, *OR* = 3.12, 95% CI: 1.96–4.27), and rated the quality of their housing more positively (*Z* = 4.59, *p* < .001, *d* = 0.43, 95% CI: 0.25–0.62). People allocated to HF were also more likely to be housed continually in the final 6 months (i.e., 79.57% versus 55.47%), χ^2^ (2, *n* = 170) = 11.46, *p* = .003, Cramer’s *V* = 0.26, 95% CI: 0.14–0.42).
Bitter et al. (2017)_k_	Netherlands	RCT	263	SMI	Social connectedness	The CARe Methodology compared with care as usual	People in sheltered or supported living	Kmet Quant score = 92	The mean score for model fidelity at T1 was 53.4% for CARe and 33.4% for CAU. At T2 this was 50.6% for CARe and 37.2% for CAU. The ICC for ‘team’ was .284 for social functioning.
Burnam et al. (1996)_b_	USA	RCT	276	SMI	Housing	Housing compared with care as usual	People experiencing homelessness	High risk of bias	Although housing outcomes improved from baseline for individuals assigned to the treatment condition, this differed little from individuals receiving CAU.
Caplan et al. (2023)_u_	Canada	RCT	43 parent–child dyads	CMD, SMI	Housing	Guided peer support group compared with treatment as usual	People experiencing homelessness or unstable housing	Kmet Qual score = 85	Parents in HF reported more positive changes, proportionally, in their relationships with their children, when compared with parents in the TAU group (no effect sizes reported).
Ellison et al. (2020)_b_	USA	RCT	166	SMI	Housing	MISSION-VET (Maintaining Independence and Sobriety through Systems Integration, Outreach and Networking-VETeran’s edition) compared with treatment as usual	People with a history of homelessness	High risk of bias	Veterans receiving MISSION-VET did not spend more days in housing compared with those receiving TAU during any part of the study. However, Veterans receiving MISSION-VET from peer specialists who were more adherent to the protocol showed greater housing stability between approximately 400- and 800-day postbaseline.
Fletcher et al. (2008)_b_	USA	RCT	191	SMI	Housing, Social connectedness	Integrated ACT (IACT) compared with ACT only (ACTO), or standard care (SC)	People experiencing homelessness	High risk of bias	There was a significant effect of intervention on stability of housing. The authors report continued improvement on housing until approximately 15-months postbaseline. The treatment contrast (ACTO and IACT versus SC) is significant and positive (*p* = .01) and both treatment groups reported a higher rate of stable housing than SC.
Goldfinger et al. (1999)_b_	USA	RCT	303	SMI	Housing	Housing placement (independent apartments) compared with staffed group homes	People with a history of homelessness	High risk of bias	Most study participants (76%) were in community housing of some sort at the end of the 18-month follow-up. However, 26.8% of the study participants experienced an episode of homelessness at some time during the study; 19.7% of those assigned to staffed group homes, compared with 35.3% of those assigned to the independent apartments (χ^2^ = 3.46, *df* = 1, *p* < .05, one-tailed).
Herman et al. (2011)_b_	USA	RCT	150	SMI	Housing	Critical Time Intervention (CTI) compared with treatment as usual	People experiencing homelessness	High risk of bias	Of the 117 participants with complete follow-up data, 31 (27%) experienced at least one homeless episode during the study. The odds of homelessness by treatment assignment was .22 (95% CI: .06–.88), with assignment to CTI associated with a fivefold reduction in the odds of homelessness compared with assignment to TAU.
Hurlburt et al. (1996)_b_	USA	RCT	361	SMI	Housing	The McKinney Project (Section 8 rent subsidy services + case management [CM]) compared with CM alone	People experiencing homelessness	High risk of bias	Participants who had access to Section 8 housing certificates were much more likely to achieve independent housing than clients without access to Section 8 certificates, but no differences emerged across the two different levels of case management.
Kerman et al. (2020)_u_	Canada	RCT	2111	SMI	Housing	At Home/Chez Soi Housing First compared with treatment as usual	People experiencing homelessness or unstable housing	Kmet Quant score = 88	The proportion of nights spent stably housed postrandomisation for frequent emergency department users was higher in intervention group (66.4%, 95% CI: 63.5% to 69.2%) than the control group (34.7%, 95% CI: 31.5% to 37.8%).
Kirst et al. (2020)_u_	Canada	RCT	Qual = 150 Quant = 2132	NR	Housing	At home/Chez Soi Housing First compared with treatment as usual	People experiencing homelessness or unstable housing	Kmet Quant score = 88	The increase in social network size over time relative to baseline was largest for participants allocated to HF compared with TAU (6 months: *B* = 0.25, 95% CI 0.15–0.34, *p* < 0.001; 12 months: *B* = 0.17, 95% CI 0.07–0.27, *p* < 0.001; 18 months: *B* = 0.21, 95% CI 0.11–0.32, *p* < 0.001; 24 months: *B* = 0.22; 95% CI 0.11–0.32, *p* < 0.001).
Korr and Joseph (1995)_b_	USA	RCT	95	SMI	Housing	Case management compared with routine care	People experiencing homelessness	High risk of bias	At a six-month follow-up, more than twice as many of the people assigned to case management were housed compared with the routine care participants. None of the people assigned to case management had returned to living on the streets or in shelters.
Lachaud et al. (2021)_u_	Canada	RCT	543	SMI	Housing	At home/Chez Soi Housing First compared with treatment as usual	People experiencing homelessness or unstable housing	Kmet Quant score = 88	The trajectory of Housing stability was ‘rapid and sustained’ for most of those allocated to HF (70.4%) compared with TAU (27.9%). In the HF condition, 14.2% were classed in ‘slow but sustained’ housing and 15.4% in ‘rapid then declining’ housing groups. In the TAU condition, 26.6% were classed as those who ‘never moved to housing’ and 16.9% in ‘rapid then declining’ housing.
Latimer et al. (2020)_u_	Canada	RCT	950	SMI	Housing	At home/Chez Soi Housing First compared with treatment as usual	People experiencing homelessness or unstable housing	Kmet Quant score = 96	The number of ‘days with stable housing’ were higher by 151.30 days (95% CI = 137.67–166.86) for those allocated to HF, compared with TAU. This equated to a cost difference of CAD$ 6,310.93 (95% CI: $309.31–$12,349.65). Thus, the Incremental Cost Effectiveness Ratio (ICER) was CAD$ 41.73 per each additional day of stable housing (95% CI: $1.96–$83.70).
Laurila et al. (2024)_u_	Finland	RCT	326	CMD, SMI	Housing	Individual long-term psychotherapy (LPP) compared with short-term (SPP)	People experiencing homelessness or unstable housing	Kmet Quant score = 81	Aspects of social support were assessed with the Brief Inventory of Social Support and Integration (BISSI). On all the BISSI subscales, with the exception of the size of the social network, there were statistically significant improvements in all the therapy groups over the follow-up. In the BISSI subscales, several statistically significant differences between the therapy groups were found, mostly in favor of LPP over SPP. Satisfaction in social support had increased more in LPP than SPP at the 1-year and 5-year follow-ups. More improvement in LPP than SPP was found in the availability of social support from professionals at the 1- and 2-year follow-ups. A greater increase in perceived availability of support from friends was found in LPP compared with SPP at the 3-year follow-up.
Lehman (1997)_b_	USA	RCT	152	SMI	Housing	ACT compared with treatment as usual	People experiencing homelessness	High risk of bias	People assigned to ACT spent significantly more days in stable community housing compared with TAU. People assigned to ACT spent an average of 58% fewer days on the street, 53% fewer days in jail and were significantly more satisfied with their housing at the 6-month, but not 2- or 12-month follow-up.
Lemoine et al. (2021)_u_	France	RCT	704	SMI	Housing	Un Chez Soi d’Abord Housing First compared with treatment as usual	People experiencing homelessness and with a history of offending	Kmet Quant score = 95	Over participants’ lifetimes, individuals in the HF group spent an average of 2685 days (95% CI: 2121–2913) in independent housing, compared with an average of 775 days for individuals in the TAU group (95% CI: 513–1346). Mean survival did not differ substantially between the two groups (HF: 11.41 versus TAU: 10.25). The costings equated to a mean cost of €320,039 (95% CI: €149,128–€808,839) in the HF group versus €309,876 (95% CI: €79,683–€829,315) in the TAU group. HF resulted in cost-savings in health service use, but increased costs associated with social services and welfare benefits. Overall, the ICER for the HF group compared with that of the TAU group was €5.3 per each additional day spent in independent housing.
Lipton et al. (1988)_b_	USA	RCT	49	SMI	Housing	Residential treatment program after discharge from inpatient care compared with standard care	People experiencing homelessness	High risk of bias	Overall, participants assigned to the residential treatment program, compared with standard postdischarge care, spent more nights in adequate shelter, fewer nights in hospitals/undomiciled and were more satisfied with their living arrangements. Although the authors note these findings are limited by a small sample size and case attrition.
Loubiere et al. (2022)_u_	France	RCT follow-up	703	SMI	Housing	Un Chez Soi d’Abord Housing First compared with treatment as usual	People experiencing homelessness and with a history of offending	Kmet Quant score = 100	The number of days spent in independent housing increased more over the study period in the HF group than in the TAU group (28.6 [95% CI: 25.1–32.1], *p* < 0.001).
McHugo et al. (2004)_b_	USA	RCT	121	SMI	Housing	Integrated housing compared with parallel housing	People experiencing homelessness	High risk of bias	The receipt of integrated housing services led to more days in stable housing than the parallel housing services control condition, especially for male participants.
Mejia-Lancheros et al. (2020)_u_	Canada	RCT	381	SMI	Housing	At home/Chez Soi housing first compared with treatment as usual	People experiencing homelessness	Kmet Quant score = 96	Participants allocated to HF did not have a significantly lower risk of an incident violence-related TBI event (adjusted hazard ratio: 0.58 [95% CI: 0.29–1.14]) compared with TAU, but they did have a significantly lower number of physical violence-related TBI events (unadjusted incidence rate ratio [IRR]: 0.22 [95% CI: 0.06–0.78]; adjusted IRR: 0.15 [95% CI, 0.05–0.48]).
Morse et al. (1992)_b_	USA	RCT	116	SMI	Housing	Assertive outreach and ICM compared with traditional outpatient treatment or a drop-in center.	People experiencing homelessness	High risk of bias	At a 12-month follow-up, participants from all three conditions spent fewer days homeless per month, and had increased income, interpersonal adjustment, and self-esteem. However, people assigned to the assertive outreach continuous treatment program had more contact with their treatment program, were more satisfied with their program, spent fewer days homeless, and used more community services and resources than people in the traditional outpatient or drop-in center conditions.
Morse et al. (1997)_b_	USA	RCT	165	SMI	Housing	ACT with additional support or ACT alone or case management	People experiencing homelessness	High risk of bias	Compared with people assigned to case management, those assigned to ACT alone, and ACT with additional support from community workers reported a greater number of contacts with the assigned treatment program, better resource utilisation (for example, use of entitlements), increased activity level, and satisfaction with the treatment program. People assigned to ACT alone also achieved more days in stable housing than those in the other two treatment conditions. No treatment group effects were found on income.
Morse et al. (2006)_b_	USA	RCT	149	SMI	Housing	Integrated ACT (IACT), or Active Community Treatment only (ACTO) compared with treatment as usual	People experiencing homelessness	High risk of bias	There was a statistically significant main effect of treatment on stable housing (*F*_2,145_ = 3.76, *p* = .03, *η* ^2^ = .05). Post-hoc analyses indicated that clients in both the ACTO and IACT conditions had significantly more days in stable housing than TAU. There was no significant difference between the IACT and ACTO clients in terms of days in stable housing. There was also a statistically significant main effect of time on stable housing (*F*_3,440_ = 66.20, *p* < .001, *η* ^2^ = .31). Over time, these participants generally increased the numbers of days in stable housing.
Mötteli et al. (2022)_u_	Switzerland	Pragmatic RCT	58	SMI	Housing	Independent supported housing (ISH) compared with treatment as usual	People experiencing housing problems	Kmet Quant score = 92	Over the observed study period of 1 year, almost all participants allocated to ISH were able to live independently, such that the need for inpatient treatment could be significantly reduced: moved from supported housing to independent housing (ISH *n* = 4, TAU *n* = 0); moved from independent housing to supported housing (ISH *n* = 1 who did not use the intervention service as intended, TAU *n* = 3); became homeless (ISH *n* = 0; TAU *n* = 1); moved to a residential care home (ISH *n* = 0, TAU *n* = 1); moved apartments (ISH *n* = 14, TAU *n* = 14). However, social inclusion scores on the Social Functioning Scale and Social Support scores on the ENRICHED Social Support Inventory reduced over time slightly for both groups.
O’Campo et al. (2023)_u_	Canada	RCT – secondary analysis	653	CMD, SMI	Housing	At home/Chez Soi housing first compared with treatment as usual	People experiencing homelessness	Kmet Quant score = 100	At the end of follow-up, the mean percentage of days spent stably housed was higher (*p* < .001) for women in the intervention (74.8%, 95% CI: 71.7% to 77.8%) compared with women in the TAU group (37.9%, 95% CI: 34.4% to 41.3%). Social outcomes were similar for both groups at 6-, 12-, 18- and 24-month postenrollment. At 24 months, the mean change from baseline for community functioning (HF: 3.8, 95% CI 2.8–4.9; TAU: 4.8, 95% CI 3.6–6.0, *p* = .236), psychological community integration (HF: 2.0, 95% CI 1.5–2.4; TAU: 2.0, 95% CI 1.4–2.6, *p* = .941), and the rate ratio for physical community integration (HF: 0.97, 85% CI 0.86–1.08; TAU: 1.03, 95% CI 0.92–1.14, *p* = .439) were similar between both groups.
O’Connell et al. (2023)_u_	USA	RCT – secondary analysis	272	CMD, SMI	Housing	Rent subsidies + intensive case management, intensive case management (ICM-only), or care as usual	People experiencing homelessness	Kmet Quant score = 83	All participants showed sizable improvements in days housed, days homeless, employment, income, and social support with moderate-to-large effect sizes (Cohen’s *d*) ranging from 0.30–3.80 on all but two measures.
Raven et al. (2020)_u_	USA	RCT	423	SMI	Housing	‘Abode’ compared with treatment as usual	People experiencing chronic homelessness	Kmet Quant score = 96	The ‘Abode’ intervention group were more likely to be housed in study period compared with TAU (odds ratio [OR]: 22.34, 95% CI: [11.69, 42.68]). The ‘Abode’ intervention group also had fewer days in shelter compared with TAU (IRR: 0.30, 95% CI: 0.17, 0.53). There were no substantial differences in jail days in the intervention group compared with TAU (IRR: 1.01, 95% CI: 0.73, 1.40).
Shern et al. (2000)_b_	USA	RCT	168	SMI	Housing	‘Choices’ intervention (an intensive case management program of outreach and engagement) compared with treatment as usual	People experiencing homelessness	High risk of bias	Participants assigned to the ‘Choices’ intervention had less difficulty meeting their basic needs, spent less time on the streets (55% vs 28% reduction), and spent more time in community housing (21% vs 9% increase) compared with TAU.
Somers et al. (2017)_k_	USA	Unblinded RCT	297	SMI	Housing	Housing First in both scattered site (SHF) and congregate (CHF) formats compared with treatment as usual	People experiencing homelessness	Kmet Quant score = 92	During the 24-month follow-up period, the % of time spent in stable housing was significantly higher in both intervention arms compared with TAU. Using the intent-to-treat analyses (*n* = 297), the intervention effect (mean difference between intervention and TAU condition) was 48.0% (95% CI: 40.0–56.3) for CHF and 48.2% (95% CI: 39.5–56.9) for SHF. Mean change from baseline to 24-month follow-up did not differ significantly between SHF and TAU for community integration on physical (0.47, 95% CI: −0.14 to 1.09) or psychological subscales (−0.34, 95% CI: −1.88 to 1.20), community functioning (1.66, 95% CI: −1.59 to 4.92), or recovery (0.05, 95% CI: 3.63–3.74). A difference approaching statistical significance (*p* = 0.057) was observed for food security and favouring TAU compared with SHF at 24 months (0.99, 95% CI: −0.02 to 2.01). Mean change from baseline to 24-months was significantly greater in CHF compared with TAU for psychological community integration (2.53, 95% CI: 1.05–4.01) and recovery (5.58, 95% CI: 1.65–9.50). No differences between CHF and TAU were observed for physical community integration (0.47, 95% CI: −0.14 to 1.09), or food security (0.99, 95% CI: 0.02–2.01).
Stergiopoulos et al. (2015)_b_	Canada	Unblinded RCT	1198	SMI	Housing	At home/Chez Soi housing first compared with care as usual	People experiencing homelessness	High risk of bias	At the 24-month follow-up, the adjusted % of days stably housed was higher among the intervention group than the usual care group, although the adjusted mean differences varied across study cities (Site A: 417.3 of 683.0 days [62.7%] for the intervention group vs 189.2 of 621.6 days [29.7%] for the usual care group, mean difference [MD], 33.0% [95% CI, 26.2% to 39.8%]; Site B: 491.5 of 653.4 days [73.2%] for the intervention group vs 157.0 of 606.8 [23.6%] for the usual care group, MD, 49.5% [95% CI, 41.1% to 58.0%]; Site C: 506.7 of 658.1 days [74.4%] for the intervention group vs 255.2 of 626.2 days (38.8%) for the usual care group, MD, 35.6% [95% CI, 29.4% to 41.8%]; Site D: 520.4 of 651.5 days [77.2%] for the intervention group vs 223.1 of 649.1 for the usual care group [31.8%], MD, 45.3% [95% CI, 38.2% to 52.2%]; *p* < .001 for interaction).
Susser et al. (1997)_b_	USA	RCT	96	SMI	Housing	Critical time intervention compared with care as usual	Men experiencing homelessness	High risk of bias	Over the 18-month follow-up period, the average number of homeless nights for people assigned to Critical Time Intervention was 30, compared with 91 for those assigned to CAU. After the 9-month period of active intervention had ended, survival curves demonstrated that this difference between the two groups did not diminish.
Tsemberis et al. (2004)_b_	USA	RCT	206	SMI	Housing	Immediate Housing compared with housing contingent on treatment and sobriety	People experiencing homelessness	High risk of bias	Participants in the Immediate Housing condition had significantly faster decreases in homeless status and increases in housing stability status compared with participants in the Housing contingent condition (F_4,137_ = 10.1, *p* < .001; F_4,137_ = 27.7, *p* < .001). Statistically significant differences were present at all the 6-, 12-, 18-, and 24-month timepoints, with the Immediate Housing group reporting less time spent homeless and more time spent stably housed compared with the control group.
Tinland et al. (2020)_b_	France	RCT	703	SMI	Housing	Scattered housing compared with treatment as usual	People experiencing homelessness	High risk of bias	Participants assigned to the HF group exhibited higher housing stability (difference in slope, 116 [103–128]). Mean difference in costs was €–217 per participant over the 24-month period in favor of the HF group. Delivery of the HF intervention was associated with cost savings in healthcare utilisation costs (RR: 0.62[0.48–0.78]) and residential costs (RR: 0.07 [0.05–0.11]).
People with an offending history									
Chandler and Spicer (2006)_b_	USA	RCT	182	SMI	Offending	Integrated Dual Disorders Treatment program compared with treatment as usual	People with an offending history	High risk of bias	Both groups showed reductions in arrests between the baseline and study period, where this difference was greater for those in the experimental group (arrests per year per person: experimental, pre = 2.89, post = 2.21; control, pre = 2.84, post = 2.61). Conviction rates reduced for those in the experimental group but not for controls (convictions per year per person: experimental, pre = 0.69, post = 0.59; control, pre = 0.61, post = 0.73). Felony convictions increased slightly for both groups (felony conviction per year per person: experimental, pre = 0.29, post = 0.31; control, pre = 0.25, post = 0.28) and jail days decreased for both groups (jail days per year per person: experimental, pre = 96.74, post = 60.71; control, pre = 79.43, post = 59.39).
Cosden et al. (2005)_b_	USA	RCT	235	SMI	Offending	Mental health treatment court with assertive community treatment compared with treatment as usual	People with an offending history	Some concerns	Offenders with a high conviction rate during the program experienced increased offending outcomes after entering the study, both in TAU and treatment groups (main effect for time (pre-post intervention) on outcomes: bookings *F*(1,20) = 33.46, *p* < .001; convictions *F*(1,20) = 17.74, *p* < 0.001; jail days *F*(1,20) = 43.51, *p* < .001). However, most of the sample did not fall into this group of high offending. For these remaining participants, an increase in bookings pre-post intervention was observed in the treatment group which was negligible in the TAU group (mean number of bookings: treatment, pre = 2.97 (SD = 4.42), post = 5.33 (SD = 6.14); TAU, pre = 3.88 (SD = 6.13), post = 3.89 (SD = 5.12); *F*(1,185) = 5.05, *p* < .05). Number of convictions reduced very slightly for both groups (treatment, pre = 1.84 (SD = 2.19), post = 1.82 (SD = 2.04); TAU, pre = 2.32 (SD = 2.54), post = 2.04 (SD = 2.93); no *p*-value reported). Number of days in jails reduced in both treatment and TAU, with a greater reduction noted for the treatment group (treatment: pre = 39.44 (SD = 62.87), post = 24.55 (SD = 39.08); TAU: pre = 47.30 (SD = 71.86), post = 37.51 (SD = 45.11)).
Cusack et al. (2010)_b_	USA	RCT	134	SMI	Offending	Forensic assertive community treatment (FACT) compared with treatment as usual	People with an offending history	High risk of bias	FACT led to fewer bookings (Raw mean [SD], 12-month follow-up: FACT = 0.64[1.2], TAU = 1.42[1.86]; 13–24 month follow-up: FACT = 0.57[1.13], TAU = 0.89[1.82]) and an increased likelihood of staying out of jail (Raw mean [SD], 12-month follow-up: FACT = 0.75[0.77], TAU = 0.85[1.03]; 13–24 month follow-up: FACT = 0.38[0.73], TAU = 0.55[0.90]) and a shorter jail time once in jail (Raw mean [SD], 12-month follow-up: FACT = 18.5[45.3]), TAU = 35.3[56.9]); 13–24 month follow-up: FACT = 20.5[63.7]), TAU = 30.5[51.6]).
Kingston et al. (2018)_b_	Canada	RCT	101	SMI	Offending	Reasoning and Rehabilitation-II Intervention compared with treatment as usual	People with an offending history	High risk of bias	Recidivism data were available for 80 participants who were followed up for an average of 1.5 years after release, whereby those in the treatment group had a slightly lower rate of violent recidivism (13.6% vs 16.7%) but a comparatively increased rate of general recidivism (59.1% vs 52.8%).
Lamberti et al. (2017)_b_	USA	RCT	70	SMI	Offending	Rochester Forensic ACT Model (FACT) compared with treatment as usual	People with an offending history	High risk of bias	Those receiving FACT, compared with TAU, had fewer mean convictions (0.4 (SD = 0.7) vs .0.9 (SD = 1.3), *p* = .023), days in jail (21.56 (SD = 25.9) vs 43.5 (SD = 59.2), p = .025), arrests (0.8 (SD = 1.3) vs 1.3 (SD = 1.7), *p* = .165), and number of incarcerations relating to new crimes (1.3 (SD = 1.5) vs 1.5 (SD = 2.2), *p* = .967).
Rowe et al. (2007)_b_	USA	RCT	114	SMI	Offending	Peer support group intervention compared with treatment as usual	People with an offending history	High risk of bias	Controlling for baseline levels of criminal justice charges, both control (standard services) and intervention groups showed lower number of criminal charges over time (Mean total charges (SD): control, pre = 1 (1.53), time 1 = 0.76 (1.50), time 2 = 0.32 (0.76); Intervention, pre = 1.40 (2.38), time 1 = 1.18 (1.87), time 2 = 0.75 (1.71); *F* = 4.30, *df* = 1 and 111, *p* < .05, *η*2 = .04). The authors stated that there was no main effect on criminal justice involvement but did not report any associated data.
Sacks et al. (2004)_b_	USA	RCT	185	SMI	Offending	Modified Therapeutic Community (MTC) with/without aftercare compared with treatment as usual (MH only)	People with an offending history	High risk of bias	This study found that those in the MTC group had lower rates of reincarceration compared with those assigned to MH only, and that after care decreased reincarceration rates further (MH only = 33%, MTC-prison only = 17%, and MTC-prison + MTC aftercare = 5%); the intervention was also associated with lower criminal activity (MH only = 67%, MTC-prison only = 53%, and MTC-prison + MTC aftercare = 42%) and longer time until incarceration (mean days (SD): MH only = 108.43 (87.80), MTC only = 124.80 (113.56), MTC + aftercare =169.50 (60.10)) or first crime (mean days(SD): MH only = 66.19 (85.33), MTC only = 84.06 (98.76), MTC + aftercare =67.11 (67.99)).
Sacks et al. (2012)_b_	USA	RCT	127	SMI	Offending	Re-entry Modified Therapeutic Community (RMTC) compared with a parole group	People with an offending history	High risk of bias	Reincarceration rates and self-reported criminal activity outcomes were much lower in the RMTC group compared with the parole group at 12-months postrelease (Reincarceration: RMTC = 19%, Parole group = 38%, OR = 0.387, 95% CI: 0.155–0.97, *p* = 0.43; Criminal activity: RMTC = 39%, Parole group = 62%, OR = 0.394, 95% CI: 0.166–0.937, *p* = .35).
Swinkels et al. (2023)_u_	Netherlands	RCT	102	SMI	Social connectedness, Offending	Social Network intervention compared with treatment as usual	People with a history of offending	Kmet Quant score = 100	Participants in the TAU group reported 2.9 times more criminal behaviours compared with participants in the intervention group overall (RR = 0.346, 95% CI: 0.152–0.787, *p* = .011). At 12-month follow-up participants in the intervention condition reported lower criminal behaviours but parameters were consistent with values indicating both increased and decreased criminal behaviours (RR = 0.575, 95% CI: 0.225–1.47). At 18-month follow-up, TAU participants showed 5.6 times more criminal behaviours compared with participants in the treatment condition (RR = 0.180, 95% CI: 0.053–0.611, *p* = .006).
Mothers									
Holt et al. (2021)_u_	Australia	RCT	77	CMD	Family	‘HUGS’ Intervention (CBT plus a mother-infant interaction intervention) compared with a control playgroup	Mothers	Kmet Quant score = 100	At a 6-month follow-up, but not immediately postintervention, among mothers allocated to HUGS, there were minor improvements in parental positive affective involvement and verbalisation (F1,47 = 4.96, *η*p2 = 0.10, *p* = .03) and reductions in measures of impaired bonding (F1,45 = 4.55, *η*p2 = .09, *p* = .04) compared with those in the control playgroup. There were also steady reductions observed in maternal parenting stress in both conditions both postintervention (F1,51 = 0.47, *p* = .50) and during follow-up (F1, 45 = 0.28, *p* = .60).
Oxford et al., 2021_u_	USA	RCT	252	CMD	Family	‘Promoting First Relationships’ (PFR) compared with receiving a resource pack in the mail	Mothers	Kmet Quant score = 100	PFR had a small positive effect on parenting sensitivity (ds = .25 and .26 at 6 and 12 months, respectively), a small effect on maternal understanding of infant behavior at 6-months (d = .21) and small-to-medium at 12-months (d = .45). There were no clear differences between the two groups on maternal confidence.
Perkins et al. (2023)_u_	UK	Unblinded RCT	89	CMD	Family	‘Songs from Home’ compared with wait-list control	Mothers	Kmet Quant score = 100	Both the intervention group and control group reported lower loneliness scores on the UCLA 3-Item Loneliness scale at week six (intervention drop: 38% relative and 25% absolute; control drop: 10% relative and 7% absolute). A large effect between social connectedness and treatment group was reported (F2,114 = 11.949, p < .001, *η*p2 = 0.173), with greater improvements observed in the intervention group (14% relative increase and 7% absolute increase) compared with controls.
Van Lieshout et al. (2021)_u_	Canada	RCT	403	CMD	Social connectedness, Family	Peer-delivered group online CBT-based workshop compared with wait-list control	Mothers	Kmet Quant score = 100	Postintervention, mothers reported improvements in bonding with their infant (B = −3.22; 95% CI, −4.72 to −1.71; p < .001; Cohen d = 0.34) and in ratings of social support on the Social Provision Scale (B = 3.31; 95% CI, 1.04–5.57; p < .001; Cohen d = 0.24). It is important to note that those lost to follow-up reported lower household income on average ($64,454 vs $101,414; t403 = 2.84; *p* = .007), suggesting poorer acceptability and feasibility for mothers from low-income backgrounds.
Caregivers									
Martin-Carrasco et al. (2016)_k_	Spain, Portugal	RCT	223	SMI	Family	Psycho-educational intervention program (PIP) compared with treatment as usual	Caregivers of a person with SMI	Kmet Quant score = 96	PIP was associated with reduced caregiver burden on the Zarit Burden Interview compared with usual care at both 4-month (mean difference = −4.33; 95% CI −7.96, −0.71) and 8-month follow-up (mean difference = −4.46; 95% CI −7.79, −1.13); more pronounced improvements were observed in caregivers without external support compared with caregivers with existing external support. Furthermore, the social dysfunction of the General Health Questionnaire–28 demonstrated a significant interaction favoring PIP (p = .005), seemingly explained by a moderate effect size at 4-month follow-up (SMD = –0.46) which disappeared at 8-month follow-up (SMD = –0.11).
Perlick et al. (2018)_k_	USA	RCT	43	SMI	Family	Family Focused Treatment (FFT-HPI) compared with standard health education	Caregivers of a person with bipolar disorder	Kmet Quant score = 88	Allocation to FFT-HPI was associated with reduced mean caregiver burden on the Social Behavior Assessment Scale (baseline = 0.76, postintervention = 0.39, 6-month follow-up = 0.26) compared with health education (baseline = 0.70, postintervention = 0.64, 6-month follow-up = 0.41).
People experiencing economic disadvantage									
Baller et al. (2020)_u_	USA	RCT	2,160	SMI	Employment	Supported Employment compared with care as usual	SSDI Beneficiaries	Kmet Quant score = 100	Participants receiving supported employment were 2.6 times more likely than those in the control group to report any earnings (*x* ^2^ = 12.1, *p* < .001) and on average, they earned $737 more over the year than the control group. Over time, their earnings grew by an average of $134 more per year in the intervention group than in the control group. There were no differences between the groups in disability benefit suspension or termination (*x* ^2^ = 2.17, *p* = 1.41).
Karasz et al. (2021)_u_	Bangladesh	Pilot RCT	48	NR	Financial	Asha (HOPE) Project	Low-income women	Kmet Quant score = 88	Asha demonstrated excellent feasibility (100% retention) and improvements in mean differences from baseline to 12-month follow-up in social support (ASHA mean difference: 23.5, control mean difference: 11.3, *p* = .024, 95% CI: −22.6 to −1.7) including tangible support (ASHA mean difference: 3.4, control mean difference: 1.5, *p* = .153, 95% CI: −4.6 to 0.7), affectionate support (ASHA mean difference: 5.4, control mean difference: 1.5, *p* = .001, 95%CI: −6.2 to −1.6), positive social interaction (ASHA mean difference: 4.1, control mean difference: 1.0, *p* = .015, 95% CI: −5.6 to −0.6), emotional support (ASHA mean difference: 8.8, control mean difference: 6.6, *p* = .443, 95% CI: −8.0 to 3.5) and additional support (ASHA mean difference: 1.8, control mean difference: 0.8, *p* = .030, 95% CI: −1.8 to −0.1). Participation in ASHA was also associated with improved household economic decision-making (ASHA mean difference: 1.5, control mean difference: −0.1, *p* = .011, 95% CI: −2.8 to −0.4). Participants assigned to ASHA also reported slightly greater reductions in experiences of physical or mental coercion than the control arm (ASHA mean difference: −0.5, control mean difference: 0.1, *p* = .011, 95% CI: 0.2–1.2).
Older adults									
Granholm et al. (2005)_b_	USA	RCT	76	SMI	Social connectedness	Cognitive behavioral social skills training and treatment as usual compared with treatment as usual only	Middle and older adults	High risk of bias	CBSST was associated with more frequent social functioning activities according to the Independent Living Skills Survey (F = 6.96, df = 1, 68, p = 0.02, *η*2 = 0.08) than those allocated to usual care postintervention, although general social skills did not differ as substantially (F = 3.92, df = 1, 68, p = 0.052, *η*2 = 0.05).
Rajji et al. (2022)_u_	Canada	RCT	63	SMI	Social connectedness	CBSST compared with treatment as usual	Older adults	Kmet Quant score = 100	CBSST was more efficacious in preventing decline in social function over a one-year period, as the trajectories of the Independent Living Skills Survey demonstrated better function in this group at both 36 weeks (Cohen’s *d* = 0.75) and 52 weeks (Cohen’s *d* = 0.92).
Ruiz-Comellas et al. (2022)_u_	Spain	RCT	90	CMD	Community participation	Group moderate-intensity aerobic physical activity program compared with care as usual	Older adults	Kmet Quant score = 92	The authors report improvements in the intervention group in social support according to change scores on the DUKE-UNC (Intervention change scores: −3.59 (11.68), 95% CI: −7.66 to 0.49; Control change scores: 2.97 (9.81), 95% CI: −0.35 to 6.29, *p* = .078; noting that parameter values are consistent with both substantially increased and slightly decreased social support) along with very high satisfaction ratings and good adherence levels (75%).
Minoritised ethnic groups									
Stergiopoulos et al. (2016)_k_	Canada	Unblinded RCT	237	SMI	Housing	Adapted Housing First (adapted-HF) compared with care as usual	People from an ethnic minority group who are experiencing homelessness	Kmet Quant score = 92	Those assigned to adapted-HF reported improved community integration over the study period (change in mean difference = 2.2, 95% CI 0.06–4.3). Similarly, assignment to adapted-HF was associated with more housing stability compared with those assigned to usual care (HF: 75%, 95% CI 70–81, CAU: 41%, 95% CI 35–48). It is important to note that a baseline diagnosis of psychosis was associated with a reduced likelihood of being housed for >50% of the study period (OR = 0.37, 95% CI 0.18–0.72).
Women with experience of intimate partner violence									
Johnson et al. (2020)_u_	USA	RCT	172	CMD, SMI	Trauma, Victimisation	‘HOPE’ (Helping to Overcome PTSD through Empowerment) compared with Person-Centered Therapy (PCT)	Women who have experienced intimate partner violence	Kmet Quant score = 89	Both HOPE and PCT had small-to-medium effects on mean difference severity scores for Intimate Partner Violence between baseline and postintervention (PCT: −1.33, 95% CI: −1.63 to −1.03, HOPE: −1.32, 95% CI –1.62 to −1.02) baseline and 6-month follow-up (PCT: −1.35, 95% CI: −1.65 to −1.05, HOPE: −1.12, 95% CI: −1.42 to −0.83), and baseline and 12-month follow-up (PCT: −1.27, 95% CI: −1.57 to −0.98, HOPE: −1.02, 95% CI: −1.32 to −0.72) and self-rated empowerment between baseline and postintervention (PCT: 0.66 95% CI: 0.36–0.96, HOPE: 0.64, 95% CI: 0.35–0.94), baseline and 6-month follow-up (PCT: 0.61, 95% CI: 0.31–0.91, HOPE: 0.60, 95% CI: 0.30–0.90), and baseline and 12-month follow-up (PCT: 0.73, 95% CI: 0.43–1.03, HOPE: 0.40, 95% CI: 0.10–0.70).
People with a comorbid intellectual disability									
Ali et al. (2021)_u_	UK	Pilot RCT	16	CMD, SMI	Social connectedness	Befriending compared with treatment as usual	People with an intellectual disability	Kmet Quant score = 92	Befriending was found to be acceptable; however, the authors experienced challenges in recruiting to this study, suggesting their recruitment strategy was not feasible for a larger RCT.

Abbreviations: NR = Not reported; _B_ = Sourced from Barnett et al., 2022; _K_ = Sourced from Killaspy et al., 2022; _U_ = Sourced from updated searches. CMD = common mental disorders; SMI = severe mental illness; ACT = assertive community treatment; IACT = integrated assertive community treatment; FACT = forensic assertive community treatment; ACTO = assertive community treatment only; ICM = intensive case management; CBT = cognitive behavioural therapy; SSDI = social security disability income.

a Quality scores were conducted using the Kmet tool for both the updated searches and studies included in Killaspy et al. (2022). Studies included in Barnett et al. (2022) were appraised using the Cochrane Risk of Bias tool.

**Table 2.** A summary of the characteristics of the included nonrandomised studies

Author, date	Location	Study design	Sample size	CMD/SMI	Life domain(s)	Intervention	Target group	Kmet score	Social and/or economic outcomes
People experiencing homelessness or unstable housing									
Brown et al. (2016)_k_	USA	Retrospective pre-post analysis	182	SMI	Housing	Housing First compared with care as usual	People experiencing homelessness	Kmet Quant score = 91	People receiving HF spent significantly less time homeless compared with people receiving usual care. HF residents were more likely to be housed (90%) after 1 year compared with care as usual (35%).
Dehn et al. (2022)_u_	Germany	Quasi-experimental	334	SMI	Housing	Supported housing compared with compared with residential care	People experiencing homelessness or unstable housing	Kmet Quant score = 100	Social functioning improved across both groups (Supported Housing: T1 = 104.9, T2 = 106; Residential Care: T1 = 104.8, T2 = 107.5), albeit with small effects (Time *x* group interaction: *F* = 1.85, *p* = .176, *ES* = .24).
Doré-Gauthier et al. (2020)_u_	Canada	Prospective longitudinal	50	SMI	Housing	Specialised assertive community treatment compared with treatment as usual	Young adults experiencing homelessness or at-risk of homelessness	Kmet Quant score = 90	Homeless young people with psychosis who received intensive assertive community intervention in addition to integrated care for early psychosis (EIS) achieved housing stability more rapidly compared with a historical cohort that received EIS alone (RR =2.38, *p =* .017).
Gutman and Raphael-Greenfield (2017)_k_	USA	Non-randomised controlled trial	15	CMD, SMI	Housing	The SMART Program and treatment as usual compared with treatment as usual only	People experiencing homelessness	Kmet Quant score = 45	At a 6-month follow-up, 57.14% of intervention group participants had transitioned into supportive housing, compared with just 25% of TAU group.
Holmes et al. (2017)_k_	Australia	Quasi-experimental prospective cohort	162	SMI	Housing	Housing First (‘Elizabeth Street Common Ground (ESCG)’)	People experiencing chronic homelessness	Kmet Quant score = 45	The average length of time spent in the ESCG accommodation by participants with psychosis was 685 days (SD = 581, *p* = 0.13). Participants with a diagnosis of schizophrenia were less likely to be evicted (9.5% vs 16.3%, *p* = 0.002) than those without a mental ill-health history or those without a diagnosis of schizophrenia.
Killaspy et al. (2016)_k_	UK	National survey	619	SMI	Housing	Residential care, supported housing, and floating outreach services	People living in supported accommodation	Kmet Quant score = 100	People in supported housing and floating outreach were more socially included, but experienced more crime, than those in residential care. Residential care was more expensive than supported housing or floating outreach.
Killaspy et al. (2020)_k_	UK	Prospective cohort	586	SMI	Housing	Supported accommodation models: Residential care, supported housing, and floating outreach services	People living in supported accommodation	Kmet Quant score = 100	A total of 243 out of 586 participants successfully moved on (residential care 15/146, supported housing 96/244, floating outreach 132/196). This was most likely for floating outreach service users (versus residential care: OR = 7.96, 95% CI: 2.92–21.69, *p* < .001; versus supported housing: OR = 2.74, 95% CI: 1.01–7.41, *p* < .001) and was associated with reduced costs of care, the promotion of human rights and recovery-based practice.
Macnaughton et al. (2018)_k_	Canada	Process evaluation	NA	SMI	Housing	At Home/ Chez Soi Housing First with Assertive Community Treatment	People experiencing homelessness	Kmet Quant score = 92	Fidelity assessments for the 10 included HF programs revealed an average score of 3.3/4, which compares favorably with other HF programs during the stages of early implementation.
Padmakar et al. (2020)_k_	India	Mixed-methods evaluation	11	NR	Housing	Supported Housing (Banyan model)	Women in need of supported housing	Kmet Qual score = 40	Pre-post data following supported housing according to the Banyan model demonstrated an improvement in social relations (no effect sizes reported, *p* < .02).
Rhenter et al. (2018)_k_	France	Qualitative evaluation of an RCT	13	SMI	Housing	L’accompagn-ment Housing First compared with treatment as usual	People experiencing homelessness	Kmet Qual score = 100	This qualitative component of the associated RCT reported identified further factors associated with recovery following HF: “(1) the need for secure space favorable to self-reflexivity; (2) a “honeymoon” effect; (3) the importance of even weak social ties; (4) support from, and hope, among peers.”
Roos et al. (2016)_k_	Norway	Qualitative evaluation	14	SMI	Housing	Sheltered Housing	People in sheltered housing	Kmet Qual score = 90	Qualitative experiences of sheltered housing: residents’ valued access to service providers who were seen as ‘ordinary people’, having fully equipped apartments (including laundry facilities) helped to reduce conflict, short-tenancy agreements made some residents feel insecure, residents spoke of the importance of having meaningful daily activities outside of the residence to avoid re-hospitalisation.
Stanhope et al. (2016)_k_	USA	Longitudinal qualitative evaluation	NA	SMI	Housing	Supportive Housing	People living in supportive housing	Kmet Quant score = 85	Qualitative interviews with case managers to determine their perspectives on supported housing revealed the following key themes: ‘believing medication to be the key to success in the program, persuading residents to take medication, and questioning the utility of the program for residents who were not medication adherent’.
Stergiopoulos et al. (2016)_k_	Canada	Qualitative fidelity evaluation	19	SMI	Housing	At home/Chez Soi Housing first	People experiencing homelessness	Kmet Quant score = 90	The project teams implementing HF achieved high fidelity scores during the baseline assessment, averaging 3.1–3.9 (scale of 0–4, where 4 represents the best possible score) on each of the Fidelity Scale’s primary domains. At an 18-month follow-up, the teams had retained or improved on these scores. The following challenges were identified: ‘(1) housing availability, the extent to which the program helps participants move quickly into permanent housing units of their own choosing; (2) contact with participants, the extent to which the program has a minimal threshold of nontreatment-related contact with participants; and (3) participant representation in the program, the extent to which participants are represented in program operations and have input into policy’.
Worton et al. (2018)_k_	Canada	Case study evaluation	6 sites	SMI	Housing	Housing first	People experiencing homelessness	Kmet Qual score = 92	Field notes and qualitative evaluations of each site produced a set of barriers and facilitators to the implementation of HF. These were found to be different for the exploration and installation stages.
Mothers									
Battle et al. (2023)_u_	USA	Open pilot trial	32 (16 couples)	CMD	Family	Family treatment for postpartum depression	Mothers	Kmet Quant score = 86	Improvements, with medium-to-large effects, were observed in family functioning postintervention across several domains, including communication (*t* = 2.5 (*df* = 8), Hedge’s *g* = 0.60, *p* = .038), role functioning (*t* = 1.6 (*df* = 8), Hedge’s *g* = 0.66, *p* = .143), general family functioning (*t* = 2.4 (*df* = 8), Hedge’s *g* = 0.51, *p* = .043), mothers’ parenting stress *(t =* 3.0 (*df* = 11), Hedge’s *g* = 0.75, *p* = .012), and ratings of key family problems *(t =* 8.3 (*df* = 5), Hedge’s *g* = 3.58, *p* < .001). There were also small improvements among fathers in parenting stress *(t =* 0.8 (*df* = 10), Hedge’s *g* = 0.22, *p* = .415), although stress ratings were lower at baseline relative to mothers.
Chaudhry et al. (2023)_u_	Pakistan	Feasibility RCT	26	CMD	Family	Integrated parenting intervention compared with routine care	Culturally adapted for mothers in Pakistani communities	Kmet Quant score = 100 Kmet Qual score = 90	The authors report 100% retention and 100% session attendance, alongside improvements in parenting stress and child socialisation scores compared with routine care, albeit not sufficiently powered to detect reliable effects.
Minoritised ethnic groups									
Edge et al. (2018)_k_	UK	Feasibility study	31	SMI	Family	Culturally adapted family intervention (CaFI)	People from an African-Caribbean background	Kmet Quant score = 65 Kmet Qual score = 65	The CaFI intervention was feasible (92% of the family units who started CaFI completed all 10 sessions) and qualitative findings also indicated acceptability of CaFI for service users, families, family support members, and healthcare professionals alike.
People experiencing economic disadvantage									
Nguyen et al. (2020)_k_	Vietnam	Pilot proof-of-concept trial	68	SMI	Community participation	Community mental health support group	Low-income households in rural Vietnam	Kmet Quant score = 68 Kmet Qual score = 45	The intervention was considered feasible and acceptable. Participants reported improvements in personal functioning (mean difference = 5.91; 95% CI: 0.29–11.53) and median annual income (preintervention median: 77.7 ± 372.5, one-year postintervention median: 120.8 ± 399.0, *p* = .02), as well as decreased median annual expenses (preintervention median: 1,488.6 ± 2,352.1, one-year postintervention median: 1,122.8 ± 1,100.2, *p* = .0004).

Abbreviations: NR = Not reported; _B_ = Sourced from Barnett et al., 2022; _K_ = Sourced from Killaspy et al., 2022; _U_ = Sourced from updated searches. CMD = common mental disorders; SMI = severe mental illness.

### People experiencing or at risk of homelessness

Targeted interventions for people experiencing homelessness or unstable housing were highly researched (*n* = 50 studies). Most interventions in this domain focused on housing for homeless/precariously housed populations (*n* = 35); the remaining literature addressed housing for people at risk of homelessness, living in sheltered/supported housing, residential care, or transitioning to community housing from sheltered accommodation.

#### Evidence from randomised studies

Fourteen randomised controlled trials (RCTs) evaluated housing first (HF) interventions (Aubry et al., 2016, 2019; Kerman et al., 2020; Kirst et al., 2020; Lachaud et al., 2021; Latimer et al., 2020; Lemoine et al., 2021; Loubière et al., 2022; Mejia-Lancheros et al., 2020; O’Campo et al., 2023; Somers et al., 2017; Stergiopoulos et al., 2015; Stergiopoulos et al., 2016; Tinland et al., 2020) or supplemented housing first (Caplan et al., 2023; Tsemberis, Gulcur, & Nakae, 2004). This approach draws on harm reduction principles, providing immediate access to housing through rent supplements and recovery-oriented support, without requirements such as sobriety. The literature mostly reported improved housing outcomes for those who received HF, namely stable housing and better-quality housing for homeless participants (Table 1).

Other included RCTs evaluated similar approaches to support people experiencing chronic homelessness into more stable housing, such as supported housing (Adamus, Mötteli, Jäger, & Richter, 2022; Mötteli et al., 2022; Raven, Niedzwiecki, & Kushel, 2020), residential treatment (Lipton, Nutt, & Sabatini, 1988), integrated housing (McHugo et al., 2004), housing placements (Burnam et al., 1996; Goldfinger et al., 1999), and interventions involving rent subsidy (Hurlburt, Hough, & Wood, 1996; O’Connell, Tsai, & Rosenheck, 2023). Types of assertive community treatment (ACT) alongside standard or integrated case management were also common in this population (Fletcher et al., 2008; Korr & Joseph, 1995; Lehman, 1997; Morse et al., 1992; Morse et al., 1997, 2006; Shern et al., 2000). The remaining studies evaluated other structured programs, such as the critical time intervention involving case management (Herman et al., 2011; Susser et al., 1997), and the Maintaining Independence and Sobriety through Systems Integration, Outreach and Networking-Veterans Edition (MISSION-VET) intervention (Ellison et al., 2020). Broadly, all of these housing interventions were associated with improved housing stability or fewer nights spent homeless. The final intervention described a supplemented long-term psychotherapy (Laurila, Lindfors, Knekt, & Heinonen, 2024) for people experiencing homelessness and reported improved social support outcomes.

#### Evidence from nonrandomised studies

The nonrandomised studies mostly evaluated HF interventions (Brown et al., 2016; Holmes et al., 2017; Macnaughton et al., 2018; Rhenter, Moreau, & L, 2018; Stergiopoulos et al., 2016; Worton et al., 2018), which similarly broadly reported favorable housing outcomes, experiences, and high fidelity of HF, alongside other types of supported housing (Dehn et al., 2022; Gutman & Raphael-Greenfield, 2017; Killaspy et al., 2016; Killaspy et al., 2020; Stanhope et al., 2016), sheltered housing (Padmakar et al., 2020; Roos et al., 2016), and specialist ACT (Doré-Gauthier et al., 2020), which broadly reported improved housing and social inclusion outcomes and experiences (Table 2).

### People with an offending history

Nine papers reported targeted interventions for people with a current or past offending history, all of which were RCTs.

#### Evidence from randomised studies

An ACT model of case management with nonadversarial court proceedings in the USA was compared with treatment as usual (TAU), assessing outcomes over a 2-year period (Cosden, Ellens, Schnell, & Yamini‐Diouf, 2005). Across both conditions, offenders with a high conviction rate experienced increased arrests (*F*_1,20_ = 33.46, *p* < .001), convictions (*F*_1,20_ = 17.74, *p* < 0.001), and jail days (*F*_1,20_ = 43.51, *p* < .001) postintervention. However, for the remaining participants, an increase in arrests postintervention was observed in the ACT group (*F*_1,185_ = 5.05, *p* < .05), whereas the number of convictions (treatment, pre = 1.84, post = 1.82; TAU, pre = 2.32, post = 2.04) and number of days in jail reduced across both groups (treatment: pre = 39.44, post = 24.55; TAU: pre = 47.30, post = 37.51).

An integrated dual disorders treatment (IDDT) program was compared with service as usual in recidivists with severe mental illness (SMI) and substance use disorders after leaving custody (Chandler & Spicer, 2006). Both groups showed reduced arrests per year, where this difference was greater for those receiving IDDT (arrests per person/year: IDDT pre = 2.89, post = 2.21; control pre = 2.84, post = 2.61). Conviction rates reduced for those receiving IDDT only (convictions per person/year: IDDT pre = 0.69, post = 0.59; control pre = 0.61, post = 0.73). Felony convictions increased slightly for both groups (felony conviction per person/year: IDDT pre = 0.29, post = 0.31; control pre = 0.25, post = 0.28) and jail days decreased for both groups (jail days per person/year: IDDT pre = 96.74, post = 60.71; control pre = 79.43, post = 59.39).

Two RCTs evaluated interventions where adaptations to ACT were applied to create forensic assertive community treatment (FACT), including accepting referrals from criminal justice agencies and making re-arrest prevention an explicit goal. FACT led to fewer bookings (12-month follow-up mean: FACT = 0.64, TAU = 1.42; 13- to 24-month follow-up: FACT = 0.57, TAU = 0.89), an increased likelihood of staying out of jail (12-month follow-up mean: FACT = 0.75, TAU = 0.85; 13- to 24-month follow-up: FACT = 0.38, TAU = 0.55), and a shorter time in jail (12-month follow-up mean: FACT = 18.5, TAU = 35.3; 13- to 24-month follow-up: FACT = 20.5, TAU = 30.5) (Cusack et al., 2010). In the second RCT, FACT led to fewer convictions (mean: 0.4 vs .0.9, *p* = .023), days in jail (mean: 21.56 vs 43.5, *p* = .025), arrests (mean: 0.8 vs 1.3, *p* = .165), and number of incarcerations relating to new offences (mean: 1.3 vs 1.5, *p* = .967) compared with TAU (Lamberti et al., 2017).

A bespoke cognitive-behavioural program targeting antisocial attitudes and recidivism was compared with TAU (Kingston, Olver, McDonald, & Cameron, 2018). Recidivism data were available for 80 participants, out of 101, who were followed up with for an average of 1.5 years after release, whereby those in the treatment group had a slightly lower rate of violent recidivism (13.6% vs 16.7%), but a slightly higher rate of general recidivism compared with TAU (59.1% vs 52.8%).

A bespoke peer support group intervention encouraging social participation and sobriety and reducing criminality was tested in 114 adults who had criminal charges within two years of enrolment in the study (Rowe et al., 2007). Controlling for baseline levels of criminal justice charges, both the control (standard services) group and intervention group showed lower rates of criminal charges over time (mean total charges: control, pre = 1, time 1 = 0.76, time 2 = 0.32; intervention, pre = 1.40, time 1 = 1.18, time 2 = 0.75; *F* = 4.30_1,111_, *p* < .05, *η*2 = .04).

Two RCTs examined a modified therapeutic community (MTC) program for men who were in prison with comorbid substance use problems. The intervention aimed to change attitudes and lifestyles associated with substance abuse, mental ill-health, and criminal thinking (Sacks et al., 2012, 2004). The first study compared MTC with a mental health treatment program (MH) in prison settings, alongside a comparison of MTC with an aftercare option when inmates were released. Those in the MTC group had lower rates of reincarceration compared with those assigned to the MH program, and aftercare decreased reincarceration rates further (MH only = 33%, MTC-prison only = 17%, and MTC-prison + MTC aftercare = 5%). The intervention was associated with lower rates of criminal activity (MH only = 67%, MTC-prison only = 53%, and MTC-prison + MTC aftercare = 42%) and a longer time to subsequent incarceration (mean days: MH only = 108.43, MTC only = 124.80, MTC + aftercare = 169.50) or first offence (mean days: MH only = 66.19, MTC only = 84.06, MTC + aftercare = 67.11).

The second study (Sacks et al., 2012) extended this work to test the effectiveness of MTC as a re-entry treatment in community correction facilities after prison release (RMTC) in comparison with parole supervision and case management. Here, reincarceration rates and self-reported criminal activity were substantially lower in the RMTC group at 12-month postrelease from prison (reincarceration: RMTC = 19%, Parole group = 38%, OR = 0.387, 95% CI: 0.155–0.97, *p* = 0.43; criminal activity: RMTC = 39%, Parole group = 62%, OR = 0.394, 95% CI: 0.166–0.937, *p* = .35).

Finally, a network coaching intervention to strengthen social networks of forensic psychiatric outpatients was compared with TAU (Swinkels et al., 2023). Participants in the intervention group reported fewer criminal behaviours compared with TAU at a 12-month follow-up (RR = 0.575, 95% CI: 0.225–1.47) and an 18-month follow-up (RR = 0.180, 95% CI: 0.053–0.611, *p* = .006).

### Mothers

Six studies evaluated targeted interventions for mothers living with mental ill-health.

#### Evidence from randomised studies

The ‘HUGS’ intervention (Holt, Gentilleau, Gemmill, & Milgrom, 2021) aimed to improve mother–infant interactions. Seventy-seven new mothers with postnatal depression in Australia were randomised to receive either a CBT session followed by a group-based mother–infant interaction intervention (‘HUGS’) or a control playgroup. HUGS was associated with improvements in parental positive affective involvement and verbalisation (*F*_1,47_ = 4.96, *η*_p_^2^ = 0.10, *p* = .03) and reductions in measures of impaired bonding (*F*_1,45_ = 4.55, *η*_p_^2^ = .09, *p* = .04) compared with the control group at 6 months.

An online peer-delivered 1-day CBT–based group workshop was adapted to address content such as social support and sleep difficulties for mothers (Van Lieshout et al., 2021). Mothers with postpartum depression (*n* = 403) in Canada were assigned to either the workshop or a waitlist control group. Mothers reported improvements in bonding with their infant (*B* = −3.22; 95% CI, −4.72 to −1.71; *p* < .001; Cohen *d* = 0.34) and in ratings of social support (*B* = 3.31; 95% CI, 1.04–5.57; *p* < .001; Cohen *d* = 0.24).

The ‘Promoting First Relationship’ (PFR) intervention, initially developed to target child welfare, was adapted to support low-income new mothers with depression, anxiety, or PTSD accessing community or primary care in the USA (Oxford et al., 2021). Two hundred fifty-two mothers received either PFR or were mailed a resource pack. The authors report small positive effects of PFR on parenting sensitivity (6 months: *ds* = .25, 12 months: *ds* = .26) and a small effect on maternal understanding of infant behaviour at 6 months (*d* = .21) and a small-to-medium effect at 12 months (*d* = .45).

The ‘Songs from Home’ intervention is a songwriting program designed to address loneliness in new mothers (Perkins, Spiro, & Waddell, 2023). Mothers with postnatal depression and experiences of loneliness in the UK (*n* = 89) were allocated to either ‘Songs from Home’ or a waitlist control. Both the intervention group and control group reported lower loneliness scores at week six (intervention drop: 38% relative and 25% absolute; control drop: 10% relative and 7% absolute). A large effect between social connectedness and treatment group was also identified (*F*_2,114_ = 11.949, *p* < .001, *η_p_^2^* = 0.173), with greater improvements observed in the intervention group (14% relative increase and 7% absolute increase, respectively).

#### Evidence from nonrandomised studies

One open pilot trial study evaluated the effects of community family treatment for 32 postpartum couples in the USA (Battle et al., 2023). Improvements, with medium-to-large effects, were observed postintervention in family functioning. A feasibility study evaluated a culturally adapted integrated parenting intervention for 26 depressed mothers in a low-income setting in Pakistan compared with routine community care (Chaudhry et al., 2023) and reported 100% retention and attendance.

### People experiencing economic disadvantage

Three studies described interventions targeted toward people experiencing specific economic disadvantage. Two further studies tested interventions developed for populations with multiple marginalised characteristics, including economic disadvantage (Chaudhry et al., 2023; Oxford et al., 2021), which are discussed in the ‘Mothers’ section. Results of interventions relating to homelessness are also relevant.

#### Evidence from randomised studies

A follow-up RCT evaluated adapted-IPS using administrative records of 2,160 individuals with schizophrenia or affective disorder who also received Social Security Disability Insurance (SSDI) payments in the USA (Baller et al., 2020). Adaptations to the IPS intervention for SSDI beneficiaries included payments of the beneficiary’s share of health insurance premiums; access to other evidence-based behavioural health services; and suspension of medical disability reviews for three years after study enrollment. Participants in the intervention group were 2.6 times more likely than those in the control group to receive any earnings, and on average earned more over the year than the control group.

The ‘ASHA’ project aimed to evaluate an integrated depression and economic strengthening intervention in rural Bangladesh (Karasz, Anne, Hamadani, & Tofail, 2021). ASHA was developed via a woman-centered framework that emphasised a woman’s right to respect, dignity, and care. Low-income women with depression (*n* = 48) were randomised to a pilot RCT of either fortnightly depression management and a financial literacy intervention followed by a cash transfer, or no intervention. The authors report improvements from baseline to 12-month follow-up in social support, such as tangible support (ASHA mean difference: 3.4, control mean difference: 1.5, *p* = .153, 95% CI: −4.6 to 0.7), positive social interaction (ASHA mean difference: 4.1, control mean difference: 1.0, *p* = .015, 95% CI: −5.6 to −0.6) and emotional support (ASHA mean difference: 8.8, control mean difference: 6.6, *p* = .443, 95% CI: −8.0 to 3.5), as well as household economic decision-making (ASHA mean difference: 1.5, control mean difference: −0.1, *p* = .011, 95% CI: −2.8 to −0.4), and reductions in experiences of physical/mental coercion compared with controls (ASHA mean difference: −0.5, control mean difference: 0.1, *p* = .011, 95% CI: 0.2–1.2).

#### Evidence from nonrandomised studies

The second study tested the acceptability, feasibility, and impact of a community mental health support group for households living in poverty, including 68 individuals with SMI and caregivers (Nguyen, Tran, & G, 2020). Group support sessions, facilitated by trained Women’s Union staff, covered topics such as personal hygiene, nutrition, physical and mental health care, rights and privileges of people with SMI, rehabilitation, community integration, and reducing caregiver stress. The intervention was reported to be acceptable and feasible, with increased annual household income and decreased annual expenditure reported.

### Older adults

Three studies considered targeted interventions for older adults.

#### Evidence from randomised studies

Three RCTs evaluated targeted interventions for older adults. Two of these (Granholm et al., 2005; Rajji et al., 2022) described modifications made to a cognitive behavioural social skills training (CBSST) intervention for older adults with schizophrenia, such as developing aids to compensate for possible cognitive impairment and integrating age-relevant content (e.g. challenging ageist beliefs and role-playing age-relevant situations). Granholm et al. (2005) reported that, of 76 middle- and older-adults recruited to either CBSST or usual care in the USA, those receiving CBSST performed social functioning activities more frequently than those allocated to usual care postintervention (*F* = 6.96, *df* = 1, 68, *p* = 0.02, *η* ^2^ = 0.08). Rajji et al. (2022) reported that of the 63 participating older adults in Canada, CBSST was more efficacious in preventing decline in social function over one-year period than usual care, as the trajectories of the Independent Living Skills Survey demonstrated better function in this group at both 36 weeks (Cohen’s *d* = 0.75) and 52 weeks (Cohen’s *d* = 0.92).

The third RCT evaluated a physical activity intervention designed to alleviate loneliness in older adults with anxiety or depression (Ruiz-Comellas et al., 2022). Participants accessing primary care services in Spain (*n* = 90) were allocated to the physical activity program or usual care. The intervention group improved in social support outcomes (intervention change scores: −3.59 (11.68), 95% CI: −7.66 to 0.49; control change scores: 2.97 (9.81), 95% CI: −0.35 to 6.29, *p* = .078).

### Caregivers

Two studies evaluated targeted intervention for caregivers.

#### Evidence from randomised studies

One multicenter RCT, conducted in Spain and Portugal, allocated 109 family primary caregivers of individuals living with schizophrenia or schizoaffective disorder to a psychoeducational intervention program (PIP) or usual care (Martin-Carrasco et al., 2016). PIP aimed to alleviate caregiver burden and improve relationships and was associated with reduced caregiver burden at follow-up compared with usual care (4 months: mean difference = −4.33; 95% CI −7.96, −0.71; 8 months: mean difference = −4.46; 95% CI −7.79, −1.13), and reduced social dysfunction (*p* = .005).

A further RCT evaluated family-focused treatment health promoting intervention (FFT-HPI) compared with standard health education among 46 caregivers of individuals living with bipolar disorder in the USA (Perlick, Jackson, & G, 2018). FFT-HPI was associated with greater reductions in caregiver burden postintervention and at 6-month follow-up (baseline = 0.76, 6-month follow-up = 0.26) compared with health education (baseline = 0.70, 6-month follow-up = 0.41).

### Minoritised ethnic groups

Two studies considered targeted interventions for minoritised ethnic groups.

#### Evidence from randomised studies

An adaptation of the HF (adapted-HF) intervention was trialed for use in Canada for individuals from Black or Asian minority ethnic backgrounds (Stergiopoulos et al., 2016). Individuals with SMI who were experiencing homelessness (*n* = 237) were recruited to an unblinded RCT of either adapted-HF or usual care. The adapted-HF intervention employed anti-racist and anti-oppressive frameworks of practice [see (Stergiopoulos et al., 2012)]. Those assigned to adapted-HF reported improved community integration over the study period (change in mean difference = 2.2, 95% CI 0.06–4.3). Assignment to adapted-HF was also associated with more housing stability compared with those assigned to usual care (adapted-HF: 75%, 95% CI 70–81, CAU: 41%, 95% CI 35–48).

#### Evidence from nonrandomised studies

A culturally adapted family intervention (CaFI) was co-produced to support individuals from Black African or Caribbean heritage living with schizophrenia, and their respective family members and/or key workers in the UK (Edge, Degnan, Cotterill, et al., 2018). A cultural adaptation framework was derived from a systematic review to identify and implement the essential elements required to tailor the family intervention to develop therapy and training manuals for CaFI. 92% of the family units who started CaFI completed all sessions, demonstrating feasibility. Qualitative findings also indicated acceptability of CaFI for service users, families/support members, and healthcare professionals alike.

### Women experiencing intimate partner violence

Only one study reported a targeted intervention adapted for women who were accessing shelter following domestic violence.

#### Evidence from randomised studies

The ‘HOPE’ intervention (Helping to Overcome PTSD through Empowerment) was developed specifically for women who had been violently assaulted by a partner and were accessing shelter. Treatment modules focused on establishing safety, improving relationships, assertiveness, anger management, and postshelter concerns. HOPE was compared with an attention-matched control, ‘Present-Centered Therapy’, among 172 women in the USA (Johnson et al., 2020). Both interventions had small-to-medium effects on mean difference severity scores for intimate partner violence between baseline and postintervention (PCT: −1.33, 95% CI: −1.63 to −1.03, HOPE: −1.32, 95% CI –1.62 to −1.02) baseline and 6-month follow-up (PCT: −1.35, 95% CI: −1.65 to −1.05, HOPE: −1.12, 95% CI: −1.42 to −0.83), and baseline and 12-month follow-up (PCT: −1.27, 95% CI: −1.57 to −0.98, HOPE: −1.02, 95% CI: −1.32 to −0.72) – and similarly for self-rated empowerment.

### People with intellectual disabilities

Only one study reported targeted intervention adapted for people with an intellectual disability.

#### Evidence from randomised studies

In a pilot RCT conducted in the UK, participants with a comorbid intellectual disability were randomised to a befriending intervention or usual care plus access to a resource booklet of local activities (Ali et al., 2021). Befrienders were matched with participants based on shared interests and availability, aiming to provide emotional and social support and facilitate access to local activities. Befriending was found to be acceptable; however, challenges in recruiting to this study occurred, indicating a lack of feasibility for a larger RCT.

## Discussion

We identified a range of targeted interventions to improve social and economic circumstances of particularly vulnerable people with mental ill-health. The interventions summarised here showed strong feasibility, acceptability and/or effectiveness across at least one social or economic outcome and highlight the potential utility for targeted interventions to improve socioeconomic inclusion for marginalised or minoritised groups. Most of these interventions were conducted in well-resourced, high-income settings, and this may limit the generalisability of findings to low- and middle-income countries or underresourced settings.

### Key findings across subgroups

The evidence base was particularly strong for targeted interventions for people experiencing or at risk of homelessness. HF represented more than half of the included studies, and these studies reported replicated positive housing outcomes. The success of this bespoke intervention emphasised the benefits of interventions designed for groups with specific needs. Rather than testing generalised interventions on broader populations first, improvements in social inclusion may be most effectively achieved if interventions are designed specifically to address the needs of the most vulnerable first, in line with the framework of proportionate universalism (Carey, Crammond, & De Leeuw, 2015).

Strikingly, with the exception of HF, there were very few replication studies resulting in a broad but heterogeneous literature base and making it difficult to draw comparisons between studies. Nevertheless, a consistent narrative emerged of the encouraging impact of targeted interventions for people with an offending history on outcomes relating to criminal behaviours, for mothers on parenting-related outcomes, and older adults on social functioning outcomes. Evidence was more disparate or sparse in relation to caregivers, people experiencing economic disadvantage, women experiencing intimate partner violence, and people with intellectual disabilities.

In particular, despite extensive research evidencing greater social adversities in people from minoritised ethnic groups, we observed a notable lack of targeted interventions for these communities – just two studies were identified (Edge et al., 2018; Stergiopoulos et al., 2016). People with mental ill-health from minoritised ethnic groups typically experience a range of social adversities, including social isolation (Morgan et al., 2008), poorer access to vocational support (Bertram & Howard, 2006), and barriers to financial health (Stacey & Smith, 2023). Furthermore, the lack of consideration of experiences of racism, complex trauma, and migration stress in the delivery of mental health services contributes to pervasive ethnic inequalities (Bansal et al., 2022). Together, this highlights the need for further intervention development.

Only a few of the identified interventions involved modifications for multiple marginalised characteristics, such as economic hardship, social roles, and demographics simultaneously. This is important from an intersectional perspective, as the most marginalised in society experience social exclusion across multiple domains (Filia et al., 2022; Kuran et al., 2020; Villatoro, Mays, Ponce, & Aneshensel, 2018), and intersectionality theory emphasises that these adversities should not be conceptualised as separable when experienced together (Crenshaw, 1989). More research is therefore warranted in this area. In a concurrent review, we identified an extensive underreporting of basic sociodemographic and intersectional features and associated stratified analyses, demonstrating key barriers to understanding what works for whom (Greenburgh et al., 2025).

### Limitations and conclusions

Several methodological limitations need to be considered in interpreting our findings. We screened for samples with diagnosed mental disorders or those who had accessed mental health services. However, many interventions exist for populations that may be vulnerable to mental ill-health but without a formal diagnosis. Thus, our approach may have missed relevant literature which is not modelled on diagnostic frameworks but rather by social circumstances. Furthermore, as we restricted our search to articles in English language and peer-reviewed journals, we likely overlooked interventions evaluated in non-English speaking countries as well as those within the grey literature. This highlights a broader problem in social intervention research, namely that key providers of support in social domains, for example third-sector organisations and local authorities, struggle to contribute to the evidence base given limited resources in tandem with day-to-day service demands.

Overall, our findings highlight that targeted social and economic interventions for people from marginalised communities who are experiencing mental ill-health may work towards addressing systemic inequalities present in mental health care. The literature base, albeit broad, is highly heterogeneous with little replication between studies. As such, these findings warrant concentrated research efforts toward existing, promising interventions to replicate findings and ultimately strengthen the evidence base to enable widespread implementation.

## Supporting information

Baldwin et al. supplementary material Baldwin et al. supplementary material

## Data Availability

The data extraction spreadsheets for this review are available upon request to the corresponding author.

## Supplementary material

The supplementary material for this article can be found at https://doi.org/10.1017/S0033291725101128.
