# Supplementary material for: Targeted interventions to improve the social and economic circumstances of people with mental ill-health from marginalised communities: a systematic review
Source: Psychol Med. 2025 Jul 28;55:e217. doi: 10.1017/S0033291725101128 (PMC12315659; doi:10.1017/S0033291725101128)
Supplement: Baldwin et al. supplementary material [file S0033291725101128sup001.pdf]

Targeted interventions to improve the social and economic circumstances of people with mental ill-health from marginalised communities: a systematic review

Supplementary materials

Supplementary Materials I: Methods

Supplementary Table I. The full inclusion and exclusion criteria used during screening for the systematic review.

n.b. the criteria are taken from Barnett et al., (2022) and Killaspy et al., (2022). Where there were discrepancies between the two reviews, the more inclusive criteria were adopted, with the exception of including secondary research, such as meta-analyses or systematic reviews.

	Inclusion criteria	Exclusion criteria
**Study design and details**	• Peer-reviewed papers reporting primary empirical data (including RCTs, feasibility and pilot trials, qualitative and mixed-method evaluations)<br>• Published between July 2020 and February 2024<br>• English language<br>• Full-text availability	• Publications that did not report primary empirical data (but relevant reviews will be labelled for reference searching)<br>• Editorials and commentaries<br>• Protocols (but relevant protocols will be labelled to identify associated publications)
**Participants**	• Adults 18+<br>• Any mental health condition or diagnosis of personality disorder established through clinical diagnosis, meeting threshold criteria on an established diagnostic screening tool or symptom severity measure; or users of specialist MH services (minimum 80% sample)	• Children and adolescents under the age of 18<br>• Intellectual/learning disability, dementia or other organic mental disorder, neurodevelopmental disorder or acquired cognitive impairment, anti-social personality disorder, adjustment disorder, substance use disorder (in the absence of any mental illness or personality disorder)
**Intervention**	• Non-pharmacological interventions **designed** to improve social circumstances in any of the included life domains **where this was the primary outcome or otherwise described in the paper as an explicit, direct focus of the intervention.** Including interventions	

	designed to improve more than one life domain, e.g. through helping people access available services, groups or community resources were also included where improving overall social circumstances was the primary aim of the programme.<br>• Social inclusion domains included: housing/homelessness; money and basic needs; work and education; social isolation and connectedness; family, intimate and caring relationships; victimisation and exploitation; offending; rights, inclusion and citizenship<br>• PLUS interventions adapted and developed bespoke for a particular sociodemographic or socioeconomic community. [N.B.]	
**Comparator**	• Any comparators can be included (or no comparator at all)	
**Outcomes**	Studies need to report at least one outcome specifically relating to the social/economic circumstances:<br>• Housing: homelessness; housing instability; housing quality<br>• Money/basic needs: poverty/income: financial barriers; debt; money management<br>• Work/education: unemployment; achieving and sustaining paid employment; precarious work; lack of access/completion of educational goals; lack of meaningful activity; length of illness absence<br>• Social isolation/connectedness: subjective social isolation/loneliness; objective SI and social network; social capital<br>• Social skills, social functioning, engagement in community-based activities, social connection, self-efficacy, hope and empowerment.<br>• Family: partner/sexual relationships; maintaining parenting roles/ contact with children; maintaining	• Instances where the only social outcomes included are those only measured through social cognition tasks e.g. emotion recognition tasks

	contact/cohabitation with family members; caring responsibilities; outcomes relating to family functioning such as expressed emotion and carer burden<br>• Victimisation: victim of crime; sexual or physical assault; domestic violence/coercive control; exploitation, harassment, safeguarding concerns<br>• Offending: risk of offending; transition from prison to community; reoffending<br>• Rights/ inclusion /citizenship: social exclusion and participation; access to public services; immigration status; privacy/dignity resulting from social circumstances<br>• Or fidelity and acceptability assessments of interventions which directly aim to improve the social or economic circumstances of people living with a mental health condition.	

N.B. This inclusion criteria was applied at the broader level from initial identification of literature (see Greenburgh et al., 2024), and a further inclusion criteria was applied for this particular research question of the systematic review: The intervention must be adapted or developed for work with a specific sociodemographic or socioeconomic population, for example of the basis of: i) Gender, ii) Age, iii) Race or ethnicity, iv) People experiencing homelessness or unstable housing, v) Low income communities, vi) Parental or caregiver roles, vii) People with an offending history, viii) People with a comorbid disability. We considered targeted interventions to be bespoke where the authors describe an intervention which was specifically created for the target population, whilst we considered adapted interventions to be where authors describe a broader, existing intervention which was altered for a particular target population.

Full search strategy, data extraction and quality assessment procedures

The Killaspy et al (2022) searches (Supplementary Materials II) were replicated in MEDLINE via Ovid, and the Barnett et al (2022) searches (Supplementary Materials III) were replicated in PsycINFO via Ovid, Web of Science (SciELO database), and the Cochrane Central Register of Controlled Trials. Database records were imported, de-duplicated, and screened on Rayyan software according to a set of inclusion criteria (see above) based upon original criteria set out in the two reviews.

Study selection was performed in duplicate by a team of researchers (HB, AG, HW, ZA) at the title and abstract and the full-text screening stages. Data extraction and quality appraisal were performed by one researcher (see data extraction form information in Supplementary Materials IV), where all data extractions were checked by a second member of the team and a random sample (10%) of quality appraisals were conducted in duplicate by a second researcher to ensure accuracy and comprehensiveness. Conflicts in inclusion decisions, extractions and appraisals were discussed with the wider review team until a consensus was reached. Quality appraisal of each included study was conducted using the Kmet quality assessment checklist (Kmet, Leanne M. ; Cook, Linda S. ; Lee, Robert C., 2004).

Data extraction fields

Data extraction was conducted according to: article information (authors; date; source [Barnett review, Killaspy review, Updated searches]; study design; country in which the research was conducted; whether the research took place in an urban, rural or mixed environment; and whether there was any lived experience in the design or conduct of the research), characteristics of the sample (sample size; diagnostic profile of the sample; whether the sample comprised only people with psychosis; proportion of the sample with substance abuse problems noted; proportion of the sample with physical health morbidities noted; ethnicity; gender; age; indicators of participant socioeconomic status; refugee status), the intervention tested (intervention name; intervention domain; comparator conditions, if applicable; setting in which the intervention was tested; whether the intervention was adapted or designed for a particular sociodemographic or socioeconomic group), and any applicable outcomes (social and/or economic participation outcome measures; clinical outcome measures; any other outcome measures; effectiveness findings – including whether analyses were stratified by gender, ethnicity or socioeconomic status, and the relevant effectiveness results in social or economic inclusion domains). Interventions were broadly categorised into nine different domains (Employment; Social connectedness and social skills; Housing; Community support/participation; Family; Education; Offending; Debt and Finance; and Trauma and Victimisation) according to their design and the primary social or economic outcome they targeted, where these categories are broadly in line with classification frameworks (Appleton et al., 2023). We note that data relating to social and/or participation outcomes were only extracted where social and/or economic participation measures could be explicitly separated (i.e. in a subscale), rather than when these factors formed part of a composite score with non-social or economic outcomes.

Supplementary Materials II:

Killaspy et al., (2022) Search strings

Ovid MEDLINE(R) ALL <1946 to February 26, 2024>

1 *Mental Disorders/ 143705

2 exp Mentally Ill Persons/ 6451

3 "Diagnosis, Dual (Psychiatry)"/ 3742

4 ("mental illness*" or "mental distress" or "mental disturbance*" or "mental disorder*" or "mentally ill").tw. 97826

5 or/1-4 212155

6 Psychotic Disorders/53383

7 Schizophrenia/ 112566

8 Affective Disorders, Psychotic/ 2319

9 Bipolar Disorder/ 45856

10 Psychoses, Substance-Induced/ 5392

11 (psychotic or psychosis or psychoses or schizophrenia or bipolar or "schizoaffective affective disorder*" or "delusional disorder*").tw. 239531

12 ("complex trauma" or "serious mental illness*").tw. 5471

13 or/6-12 297478

14 (severe or chronic or enduring or persistent or persisting or complex or "serious and continuing").tw. 4194181

15 ("high level" and (disabilit* or need*)).tw. 16968

16 ("psychosocial disabilit*" or "psycho-social disabilit*").tw. 384

17 or/14-16 4208099

18 5 and 17 35490

19 13 or 18 322714

20 exp Mental Health Services/ 106854

21 exp Community Psychiatry/ 2091

22 community health services/ or exp community mental health services/ or patient participation/ 81603

23 Residential Facilities/ 5760

24 Residential Treatment/ 3345

25 Health Services for Persons with Disabilities/ 149

26 Preventive Health Services/ 14514

27 "inpatient mental health".mp. 698

28 "psychological service*".mp. 1132

29 "Delivery of Health Care"/ 121510

30 "Delivery of Health Care, Integrated"/ 14434

31 Patient Care Management/ 4749

32 Program Evaluation/ 67493

33 "Continuity of Patient Care"/ 20811

34 "Referral and Consultation"/ 77161

35 "model* of care".tw. or "care model*".mp. 21391

36 "service delivery model*".tw. 1557

37 institutionali*.tw. 18419

38 de-institutionali*.tw. 178

39 transition*.tw. 535805

40 discharge.tw. 250147

41 "stepped care".tw. 1711

42 "shared care".tw. 1641

43 "collaborative care".tw. 3129

44 "community care".tw. 5523

45 "integrated care".tw. 6568

46 "integrated model*".tw. 4447

47 "case management".tw. 12712

48 Rehabilitation/ 18716

49 ("psychosocial rehabilitation" or "psycho-social rehabilitation" or recovery-based or "recovery based" or recovery-orient* or "recovery orient*" or "psychosocial support*" or "psycho-social support*").tw. 8369

50 Home Care Services/ 36707

51 ("home based outreach" or "outreach service*" or "assertive community treatment").tw. 1862

52 social support/ 79644

53 Employment, Supported/ 1380

54 ("supported employment" or "individual placement and support" or "competitive employment" or "peer supported work" or "peer workforce" or "vocational rehabilitation" or "employment assistance" or "supported education").tw. 3902

55 ("volunteering" or "voluntary work").tw. 2952

56 Housing/ 20455

57 ("support* housing" or "housing first" or "staffed housing" or "residential support" or "staffed residen*" or "24 hour supported community" or "twenty-four hour supported community" or "residen* rehabilitation" or "support* accommodation").tw. 1783

58 Occupational Health Services/ 10771

59 "occupation based lifestyle intervention*".tw. 4

60 "occupational time use intervention*".tw. 1

61 "Activity based intervention*".tw. 146

62 ((remotivation or re-motivation) adj2 process*).tw. 2

63 Family/ 85586

64 "family involvement".kw. 135

65 ("family psycho-education" or "family psychoeducation" or "family education" or "multiple family groups" or "family involvement" or "family support").tw. 11153

66 Cognitive Behavioral Therapy/ 31001

67 Psychotherapy/ 58435

68 Behavior Therapy/ 30772

69 ("cognitive behavioral therapy" or "cognitive behavioural therapy" or CBT or "cognitive remediation").tw.25574

70 Self Care/ 36385

71 (psychoeducation or "psycho-education" or "self-management" or "self management" or "personal* support*" or "person centered" or "person centred" or "self-help group" or "personal* assistance" or "peer support*" or "peer group" or "peer led" or "peer-let" or "care co-ordinators" or care coordinators).tw. 54352

72 Social Skills/ 2768

73 Social Adjustment/ 23707

74 ("social skills" or "social functioning" or "social network" or "social skills training" or "social cognition training" or "skills training").tw. 40572

75 ("social connection" or "social support*").tw. 56723

76 "social interventions".tw. 935

77 "Social prescribing".tw. 382

78 social participation/ or "Social participation".tw. 6774

79 Harm Reduction/ 4280

80 ("harm reduction" or "motivational interviewing").tw. 12259

81 ("early intervention" or "priority intervention" or "mental health triage" or "illness management" or "relapse prevention" or "wellness recovery action plan" or ("wellness plan*" or "safety plan*" or "recovery plan*")).tw. 30791

82 "shared decision making".tw. 14095

83 partnership*.tw. 48776

84 collaborative.tw. 77325

85 "dispersed power".tw,kw. 3

86 "trauma informed".mp. 3041

87 "group work".tw. 1382

88 "evidence informed intervention*".mp. 139

89 "evidence based intervention*".mp. 7226

90 ("psycho-social intervention*" or "psychosocial intervention*").mp. 8739

91 (NDIS or "national disability insurance scheme").mp. 446

92 ("step-down" or secure).tw. 38039

93 ("co-design" or codesign or "co-production" or coproduction).tw. 6097

94 ("open dialogue" or "open dialog").tw. 632

95 ("needs based model*" or "needs-based model").tw. 48

96 "network* model*".tw. 19773

97 or/20-96 1934518

98 Quality of Life/ or "quality of life".tw. 461603

99 Patient Readmission/ or Patient Admission/ or readmission.tw. or readmit*.tw. or admission.tw. or admit*.tw. 508452

100 Activities of Daily Living/ or activities of daily living.tw. 89239

101 Homeless Persons/ or homeless.tw. 13800

102 Occupations/ or Return to Work/ or vocation.tw. or return to work.tw. or return-to-work.tw. or back to work.tw. or back-to-work.tw. or "work participation".tw. or "vocation* outcome*".mp. 39889

103 Health Behavior/ or Risk Reduction Behavior/ or Life Style/ or exercise.tw. or tobacco.tw. or diet.tw. 920637

104 Patient Satisfaction/ or "patient satisfaction".tw. or "consumer satisfaction".tw. or "consumer recovery".tw. or "consumer outcome*".tw. 116784

105 "Carer satisfaction".tw. 98

106 (family relationship* or family connection*).tw. 5268

107 "restrictive intervention*".tw. 162

108 seclusion.tw. 1594

109 restraint*.tw. 29920

110 ("forced medication" or "forced sedation" or coercion).tw. 4291

111 (compulsion or "restrictive practice*" or adherence).tw. 165262

112 (recovery adj3 measur*).mp. 6205

113 connection.tw. 110744

114 inclusion.tw. 347253

115 "consumer experience of care".tw. 1

116 "family experience of care".tw. 21

117 (Choice or Connectedness or Hope or Optimism or Meaning or empower* or Agency or Autonomy or Growth or Wellbeing or well-being or Belonging).tw. 2606185

118 qualitative.tw. 335760

119 "mixed method*".tw. 45479

120 evidence based practice/ 12203

121 ("consumer led" or "consumer academic" or "lived experience academic").tw. 127

122 ("stigma reduction" or "stigma and discrimination").tw. 3673

123 mutuality.tw. 899

124 "personal journey".tw. 298

125 isolation.tw. 299723

126 carer.tw. 6258

127 supporters.tw. 3225

128 "significant other*".tw. 4922

129 engagement.tw. 107402

130 "relational recovery".tw. 4

131 ("therapeutic alliance*" or "therapeutic relationship*").tw. 7348

132 or/98-131 5529052

133 19 and 97 and 132 19990

134 19 and 91 12

135 133 or 134 19995

136 limit 135 to (english language and yr="2020 -Current") 4247

137 19 and 97 51754

138 limit 137 to randomized controlled trial 3192

139 Randomized Controlled Trial/ 609629

140 Randomized Controlled Trials as Topic/ 167234

141 ("randomi#ed controlled trial*" or RCT or "randomi#ed controlled study*" or "randomi#ed trial*or double blind*").mp. 900102

142 139 or 140 or 141 900102

143 137 and 142 4995

144 138 or 143 4995

145 limit 137 to (meta analysis or "systematic review") 1567

146 Meta-Analysis/ 195913

147 Meta-Analysis as Topic/ 23784

148 ("meta analys*" or metaanalys* or "systematic review*").mp. 503098

149 146 or 147 or 148 503098

150 137 and 149 2239

151 145 or 150 2239

152 144 or 151 6539

153 152 not 135 3009

154 limit 153 to (english language and yr="2020 -Current") 679

Supplementary Materials III: Barnett et al., (2022) Search strings

APA PsycInfo <1806 to February Week 4 2024>

1 clinical trials.sh. 12305

2 (randomi#ed or randomi#ation or randomi#ing).ti,ab,id. 113208

3 (RCT or at random or (random* adj3 (administ* or allocat* or assign* or class* or control* or crossover or cross-over or determine* or divide* or division or distribut* or expose* or fashion or number* or place* or recruit* or split or subsitut* or treat*))).ti,ab,id. 130161

4 ((control* adj5 (trial or study or group?)) and (placebo or waitlist* or wait* list* or ((treatment or care) adj2 usual))).ti,ab,id,hw. 26911

5 trial.ti,id. 45596

6 controlled trial.ab. 26277

7 or/1-6 190648

8 ((children? or kids or adolescen* or teens or teenagers or schools or school based) not (adult* or family or families or mother? or woman* or women* or female? or father? or men or mens or male? or relations or former)).ti. 364986

9 prevalence.ti. 23709

10 7 not (8 or 9) 175755

11 limit 10 to yr="2020 -Current" 35795

12 BIPOLAR DISORDER/ or bipolar i disorder/ or bipolar ii disorder/ or cyclothymic disorder/ or mania/ or hypomania/ or exp dissociative disorders/ or PERSONALITY DISORDERS/ or antisocial personality disorder/ or avoidant personality disorder/ or borderline personality disorder/ or dependent personality disorder/ or histrionic personality disorder/ or narcissistic personality disorder/ or obsessive compulsive personality disorder/ or paranoid personality disorder/ or passive aggressive personality disorder/ or schizoid personality disorder/ or schizotypal personality disorder/ or PSYCHOSIS/ or exp acute psychosis/ or affective psychosis/ or exp alcoholic psychosis/ or capgras syndrome/ or chronic psychosis/ or postpartum psychosis/ or reactive psychosis/ or schizophrenia/ or acute schizophrenia/ or catatonic schizophrenia/ or paranoid schizophrenia/ or process schizophrenia/ or schizoaffective disorder/ or schizophreniform disorder/ or undifferentiated schizophrenia/ or delusions/ or schizotypy/ or toxic psychoses/ 193544

13 (bipolar or cyclothymi* or mania or manic or hypermani* or rapid cycling or conversion disorder* or (dissociative adj (amnesi* or fugue* or disorder*)) or borderline state? or catatoni* or character disorder* or delusion* or capgras syndrom* or diogenes syndrom* or depersonalization or depersonalisation or de-personalization or de-personalisation or perceptual disorder* or personality disorder* or BPD or paranoi* or psychiatr* or psychopathol* or psycho-pathol* or psychotic* or psychosis* or psychoses* or schizo* or hebephreni* or serious* mental* or SMI).ti,ab,id. 548961

14 12 or 13 556617

15 11 and 14 4199

16 MENTAL DISORDERS/ or anhedonia/ or neurosis/ or ACUTE STRESS DISORDER/ or adjustment disorders/ or attachment disorders/ or disinhibited social engagement disorder/ or posttraumatic stress disorder/ or complex ptsd/ or desnos/ or acute stress disorder/ or post-traumatic stress/ or traumatic neurosis/ or *emotional trauma/ or AFFECTIVE DISORDERS/ or disruptive mood dysregulation disorder/ or dysthymic disorder/ or seasonal affective disorder/ or major depression/ or anaclitic depression/ or endogenous depression/ or late life depression/ or postpartum depression/ or

reactive depression/ or recurrent depression/ or treatment resistant depression/ or premenstrual dysphoric disorder/ or ANXIETY DISORDERS/ or generalized anxiety disorder/ or exp obsessive compulsive disorder/ or panic attack/ or panic disorder/ or exp phobias/ or trichotillomania/ or phobias/ or acrophobia/ or agoraphobia/ or claustrophobia/ or ophidiophobia/ or social phobia/ or separation anxiety disorder/ or mutism/ or elective mutism/ or EATING DISORDERS/ or anorexia nervosa/ or binge eating disorder/ or bulimia/ or SELF-INJURIOUS BEHAVIOR/ or self-destructive behavior/ or self-inflicted wounds/ or self-mutilation/ or self-poisoning/ or suicide/ or attempted suicide/ or suicidality/ or suicidal ideation/ or suicide prevention/ or SOMATOFORM DISORDERS/ or body dysmorphic disorder/ or exp conversion disorder/ or exp factitious disorders/ or hypochondriasis/ or exp hysteria/ or neurasthenia/ or neurodermatitis/ or somatization disorder/ or somatoform pain disorder/ or munchausen syndrome/ or munchausen syndrome by proxy/ 459895

17 "Depression (Emotion)"/ 27158

18 "Stress and trauma related disorders"/ 56

19 Mental Health/ or (mental* adj2 (health* or ill*)).ti,ab,id.300112

20 Mental Health Services/ or Community Mental Health Services/ or Community Counseling/ or Community Psychiatry/ 48950

21 Mental Health Program Evaluation/ 2457

22 (acute stress or adjustment disorder* or ADNOS or affective disorder* or agoraphobi* or anorexia nervosa or anxiety or astheni* or attachment disorder* or binge eat* or binging or body dysmorphi* or bulimi* or combat disorder* or obsessive or compulsi* or OCD or depression or depressed or depressive or dyssomni* or dyspareunia* or dysphori* or dysthymi* or dystoni* or eating disorder* or EDNOS or emotional trauma or fear or health anxiety or hoarding or hyperactivity or hypochondri* or hysteri* or medically unexplained or malingering or MDD or common mental or (mental* adj2 (health or well*)) or mood or moods or munchausen or MUPS or mutism or neurastheni* or neurotic or neuros* or panic or phobi* or PND or ((post-trauma* or posttrauma*) adj stress*) or psychogenic or psychosomatic or PTSD or (self adj (injur* or harm or mutilat*)) or psychosexual or (psychological adj3 sexual adj3 dysfunction*) or social* anxi* or somati* or somatoform or suicid* or parasuicid* or trichotillomani*).ti,ab,id. 1065011

23 or/16-22 1146616

24 11 and 23 13736

25 social prescribing.mp. 134

26 ((chang* or develop* or enhanc* or initiative? or intervention? or program* or mitigat* or address* or improv* or target*) adj3 (community or living or social) adj3 (condition? or circumstance?)).ti,ab,id. 1498

27 ((communit* or social) adj (connect* or engagement? or link* or referral? or intervention? or wellbeing)).ti,ab,id. 15946

28 "sense of belonging".ti,ab,id. 4477

29 or/26-28 21709

30 15 and 29 26

31 24 and 29 135

32 housing/ or assisted living/ or group homes/ or shelters/ 10482

33 residential facilit*.ti,id. 772

34 homeless/ or homeless mentally ill/ or deinstitutionalization/ 11259

35 ((chang* or develop* or enhanc* or initiative? or intervention? or program* or mitigat* or address* or improv* or target*) adj3 (housing or neighbo?rhood?)).ti,ab,id. 4789

36 homeless*.ti,ab,id. 13421

37 ((housing adj (first or stability or instability)) or permanent housing).ti,ab,id. 1283

38 housing.ti. or ((housing adj (strateg* or polic* or project* or program* or quality)) or new* buil* or social housing*).ti,ab,id. 5863

39 independent living programs/ or living arrangements/ or residential care institutions/ 15199

40 halfway houses/ or independent living programs/ or living arrangements/ or residential care institutions/ 15507

41 poverty areas/ or social environments/ 9810

42 ((autonomous or assisted or sheltered or support*) adj3 (housing or accommodation or dwelling?)).ti,ab,id. 2363

43 (((clubhouse or club house) adj model?) or ((autonomous or independent or assisted) adj living)).ti,ab,id. 5114

44 (community residences or group homes or community living or supervised apartments).ti,ab,id. 3471

45 therapeutic social clubs/ 157

46 ((independ* or assist* or support* or secur* or sustain* or maint*) adj3 (tenanc* or tenure?)).mp. [mp=title, abstract, heading word, table of contents, key concepts, original title, tests & measures, mesh word] 248

47 ((halfway or satellite) adj (dwelling? or home? or house?)).ti,ab,id. 500

48 (neighbo?rhood? adj (characteristic* or intervention* or program*)).ti,ab,id. 1460

49 ((environment* or housing or neighbo?rhood?) and infrastructure).ti,ab,id. 2510

50 built environment/ or urban planning/ 2275

51 or/32-50 64561

52 15 and 51 48

53 24 and 51 159

54 MONEY.ti,id. 4028

55 socioeconomic status/ or "income (economic)"/ or budgets/ or economic security/ or financial strain/ 39984

56 exp employee benefits/ 6927

57 *disadvantaged/ or *social deprivation/ 7035

58 ((access* or improv* or manag* or supplement*) adj2 (cash or money or financ* or income? or savings)).ti,ab,id. 4775

59 exp income level/ 20363

60 ((financial adj (autonomy or security or insecurity)) or loans or borrowing or budgeting or microcredit or microfinance or social fund*).ti,ab,id. 5378

61 high poverty.ti,ab,id. or poverty.ti. 6309

62 ((address* or escap* or improv* or support* or target*) adj2 (depriv* or poor or poverty)).ti,ab,id. 2624

63 "out of poverty".ab. 223

64 (((food or fuel) adj poverty) or food bank?).ti,ab,id. 189

65 ((alleviat* or ease or manag* or prevent* or reduc* or stop*) adj2 (poverty or ((economic or financial) adj hardship?))).ti,ab,id. 1637

66 ((alleviat* or eas* or manag* or prevent* or reduc* or relief or stop*) adj1 debt?).ti,ab,id. 115

67 debt?.ti,id. 1141

68 (austerity or recession?).ti,ab,id. 3723

69 (((basic or minimum) adj3 (wage? or income?)) or zero hours).ti,ab,id. 705

70 paid work.ti,ab,id. 1813

71 "dealing with money".ab. 17

72 (family adj (income? or tax credit?)).ti,ab,id. 4661

73 "welfare services (government)"/ or community welfare services/ or medicaid/ or welfare reform/ 9000

74 welfare benefit?.ti,ab,id. 484

75 or/54-74 106448

76 15 and 75 48

77 24 and 75 213

78 employment status/ or employability/ or occupational tenure/ or occupational status/ or job security/ or job search/ or supported employment/ or vocational rehabilitation/ or vocational evaluation/ or work adjustment training/ or sheltered workshops/ 33919

79 unemployment/ or personnel termination/ or employee layoffs/ 6046

80 ((chang* or develop* or enhanc* or initiative? or intervention? or program* or address* or improv* or target*) adj3 (employment or unemployment or unemploy*)).ti,ab,id.5738

81 (support* adj3 (employment? or work or vocational or occupation*)).ti,ab,id. 13943

82 ((job? or work* or employment* or employee? or occupation*) adj5 skills adj5 train*).ti,ab,id. 1373

83 (paid adj (job? or employment or work or occupation*)).ti,ab,id. 3133

84 (employment or unemployment or occupation*).ti. 32126

85 individual placement?.ti,ab,id. 479

86 ((finding or gaining or obtaining or keeping or sustaining) adj3 (work or job? or employment or occupation*)).ti,ab,id. 3165

87 (social firms or (sheltered adj (employment or work or occupation*))).ti,ab,id. 241

88 (precar* adj1 (job? or employment or work or occupation*)).ti,ab,id. 539

89 (voluntary work or volunteering).ti,ab,id. 3871

90 (meaningful adj (activit* or job? or employment or work or occupation*)).ti,ab,id. 2364

91 (((return or back) adj2 work) or absenteeism).ti,ab,id. 8541

92 ((alleviat* or ease or manag* or prevent* or reduc* or stop*) adj ((employment or work* or occupation*) adj disabilit*)).ti,ab,id. 47

93 (return* adj2 education).ab.207

94 ((education or learning or training) adj3 (access* or takeup or take up)).ti,ab,id. 5567

95 ((labo?r force or employment or unemployment or occupation*) adj status).ti,ab,id. 9823

96 or/78-95 99361

97 15 and 96 86

98 limit 97 to yr="2020 -Current" 86

99 24 and 96 247

100 family relations/ or intergenerational relations/ or exp marital relations/ 59388

101 family conflict/ or marital conflict/ 6855

102 home environment/ or living alone/12193

103 ((family or families or intergenerat* or inter-generat*) adj (relation* or conflict?)).ti,ab,id. 22811

104 ((sexual or intimate or partner? or marital) adj (relation* or conflict?)).ti,ab,id. 21458

105 ((develop* or enhanc* or initiative? or intervention? or program* or address* or improv* or promot* or target*) adj2 relationship?).ti,ab,id. 21676

106 ((carer? or partner or relationship? or marital) adj support*).ti,ab,id. 1892

107 (child* adj2 (access or contact or custody or maintenance)).ti,ab,id. 5865

108 child custody/ or joint custody/ or child visitation/ or divorce/ or family reunification/ or living arrangements/ 15108

109 ((care proceeding? or family court? or child removal or fostercare or foster care) and (parent* or mother? or father?)).ti,ab,id. 4146

110 parenting/ or parental involvement/ or parental role/ 29491

111 *parents/ or parent* outcome?.ti,ab,id. 29595

112 or/100-111 188026

113 15 and 112 74

114 24 and 112 457

115 parent* mental health.ti,ab,id. 1359

116 114 or 115 1793

117 (VICTIMIZATION or victimisation or revictimi#ation).ti,ab,id,hw. 36020

118 crime victims/ 5792

119 (crime victim? or revictimi*).ti,ab,id. 2263

120 ((victim* or crime?) and survivor*).ti,ab,id,hw. 5794

121 domestic violence/ or battered females/ or exposure to violence/ or intimate partner violence/ or physical abuse/ or exp sexual abuse/ or shelters/ 54588

122 elder abuse/ 2066

123 ((domestic or partner? or spouse?) adj3 (abus* or violen*)).ti,ab,id. 27276

124 ((domestic or marital or partner? or spous*) adj3 (rape or sex* assault*)).ti,ab,id,hw. 819

125 (intimate partner adj2 abus*).ti,ab,id. 760

126 interpersonal control/ or coercion/ 15642

127 coercive control.ti,ab,id. 520

128 ((female? or women?) adj (refuge? or shelter?)).ti,ab,id. 401

129 (exploitation or safe guarding or safeguarding).ti,ab,id. 10155

130 slavery/ or human trafficking/ or *freedom/ 4248

131 or/117-130 118723

132 exp *criminal offenders/ 17329

133 Recidivism.ti,id,sh. 7740

134 ((crime? or criminal* or offend* or offence? or recidiv*) adj3 (initiative? or intervention? or program* or mitigat* or address* or rehabilitat*)).ti,ab,id. 6364

135 ((crime? or criminal* or offend* or offence? or recidiv*) adj3 (diver* or prevent*)).ti,ab,id. 3999

136 prisoners/ or criminal rehabilitation/ or reintegration/ 16469

137 ((inmate? or prison* or convict? or felon? or gang member? or delinquent? or justice-involved or perpetrator? or probation) adj5 (release or integration or reintegrate or re-integrat* or rehabilitat* or re-habilitat* or desistance)).ti,ab,id. 2588

138 (community adj2 (reentry or re-entry or rehabilitat* or re-habilitat*)).ti,ab,id. 1758

139 or/132-138 44392

140 15 and (131 or 139) 85

141 24 and (131 or 139) 313

142 human rights/ or exp civil rights/ or exp freedom/ 41054

143 (((citizen? or civil* or human or legal or social or voting) adj rights) or social justice or equal protection or social protection).ti,ab,id. 27995

144 *needs/ 5787

145 rights.ti,hw. 16424

146 *government policy making/ 16885

147 ((public or social) adj polic*).ti,ab,id. 22128

148 equity-focus*.ti,ab,id. 159

149 *health disparities/ 9025

150 ((social or community or neighbo?rhood?) adj3 (equit* or inequit* or inequalit* or dispar*)).ti,ab,id. 9401

151 digital divide/ or information literacy/ 1169

152 internet access.ti,ab,id. 1331

153 (digital adj (inclusion or exclusion or divide or disparit* or equit* or inequit* or inequalit*)).ti,ab,id. 1686

154 or/142-153 120795

155 15 and 154 34

156 24 and 154 109

157 SOCIAL ISOLATION/ 9426

158 loneliness/ 7323

159 (loneliness or lonely).ti,ab,id. 15834

160 social isolation.ti,ab,id. 10712

161 ((social* or societ* or communit*) adj3 (isolated or isolation)).ti,ab,id. 13730

162 ((alleviat* or ease or manag* or mitigat* or prevent* or overcom* or reduc* or stop*) adj2 (isolation or isolated)).ti,ab,id. 1191

163 ((address* or enhanc* or improv* or increas* or promot* or target*) adj2 (inclusion or inclusivity)).ti,ab,id. 2026

164 or/157-163 34961

165 limit 164 to yr="2020 -Current" 9958

166 15 and 165 43

167 24 and 165 232

168 SOCIAL PARTICIPATION.mp. 4343

169 (social alienation or social inclusion).ti,ab,id. 3432

170 (community involvement or social support or social network or psychosocial environment or psychosocial rehabilitation).ti,id,hw. 66491

171 abandonment/ or alienation/ 3554

172 (abandonment or alienation).ti,id. 3797

173 ((social or societ* or community) adj (confine* or contact or contacts or connect* or inclusion or network* or participation or relations*)).ti,id,hw. 42232

174 ((social* or societ* or communit*) adj3 (alienat* or discriminat* or excluded or exclusion)).ti,ab,id,hw. 13385

175 ((alleviat* or ease or manag* or mitigat* or prevent* or overcom* or reduc* or stop*) adj2 (exclusion or excluded)).ti,ab,id. 323

176 (social capital or social* mobil*).ti,ab,id,hw. 14138

177 (navigator or navigators).ti,id. 342

178 peer? support*.ti,id. 2517

179 Peers/ and Social Support/ 1456

180 (anti-stigma* or ((intervention* or alleviat* or prevent* or reduc* or stop*) adj2 stigma*)).ti,ab,id. 5062

181 (social learning theory or (social adj3 interaction* adj3 (counsel* or educat* or intervention* or program* or therap* or train*)) or SCIT).ti,ab,id. 4159

182 community integration.mp. 1662

183 (peer? adj (support* or navigat*)).ti,id. 2550

184 community involvement/ or *social support/ or *social networks/ 51263

185 ((social or societ* or psychosocial) adj support*).ti,id,hw. 49099

186 ((social* or societ* or communit*) adj network*).ti,id,hw. 29176

187 ((address* or enhanc* or improv* or increas* or promot* or target*) adj3 (social* or communit*) adj3 (network? or support)).ti,ab,id. 4903

188 Psychosocial Rehabilitation/ 4760

189 ((address* or enhanc* or improv* or increas* or promot* or target*) adj2 (inclusion or inclusivity)).ti,ab,id. 2026

190 or/168-189 143458

191 15 and 190 193

192 24 and 190 630

193 30 or 52 or 76 or 98 or 113 or 140 or 155 or 166 or 191 520

194 31 or 53 or 77 or 99 or 116 or 141 or 156 or 167 or 192 3353

195 193 or 194 3489

<u># Web of Science Search Strategy (v0.1)</u>

-

<u># Database: SciELO Citation Index</u>

Entitlements:

- SCIELO.SCIELO: 2002 to 2024

Searches:

1: (TI=(trial) OR AB=(trial) OR TI=(randomized or randomised) OR AB=(”randomized controlled trial” or “randomised controlled trial” or RCT) or AB=(random* SAME (administ* or allocat* or assign* or class* or control* or crossover or cross-over or determine* or divide* or division or distribut* or expose* or fashion or number* or place* or recruit* or split or subsitut* or treat*))) Timespan: 2020-07-01 to 2024-02-28 Date Run: Wed Feb 28 2024 11:02:19 GMT+0000 (Greenwich Mean Time) Results: 10264

2: TI=(children or boys or girls or kids or adolescen* or teen* or school or schools) Timespan: 2020-07-01 to 2024-02-28 Date Run: Wed Feb 28 2024 11:02:54 GMT+0000 (Greenwich Mean Time) Results: 10638

3: (#1 not #2) Timespan: 2020-07-01 to 2024-02-28 Date Run: Wed Feb 28 2024 11:03:16 GMT+0000 (Greenwich Mean Time) Results: 9901

4: (TS=(”serious* mental*” or schizo*) or TI=(bipolar or cyclothymi* or mania or manic or hypermani* or “rapid cycling” or “conversion disorder*” or “dissociative amnesi*” or “dissociative fugue*” or “dissociative disorder*” or “borderline state*” or catatoni* or “character disorder*” or delusion* or “capgras syndrom*” or “diogenes syndrom*” or depersonalization or depersonalisation or de-personalization or de-personalisation or “perceptual disorder*” or “personality disorder*” or BPD or paranoi* or psychiatr* or psychopathol* or psycho-pathol* or psychotic* or psychosis* or psychoses* or hebephreni* or SMI)) Timespan: 2020-07-01 to 2024-02-28 Date Run: Wed Feb 28 2024 11:03:37 GMT+0000 (Greenwich Mean Time) Results: 990

5: (#4 AND #3) Timespan: 2020-07-01 to 2024-02-28 Date Run: Wed Feb 28 2024 11:03:53 GMT+0000 (Greenwich Mean Time) Results: 49

6: (TS=(“mental health” or “mental* ill*” or “common mental” or “mental disorder*” or psychiatric) or TI=(“acute stress” or “adjustment disorder*” or ADNOS or “affective disorder*” or agoraphobi* or “anorexia nervosa” or anxiety or astheni* or “attachment disorder*” or “binge eat*” or binging or “body dysmorphi*” or bulimi* or “combat disorder*” or “obsessive or compulsi*” or OCD or depression or depressed or depressive or dyssomni* or dyspareunia* or dysphori* or dysthymi* or dystoni* or

“eating disorder*” or EDNOS or “emotional trauma” or fear or “health anxiety” or hoarding or hyperactivity or hypochondri* or hysteri* or “medically unexplained” or malingering or MDD or mental* or mood or moods or munchausen or MUPS or mutism or neurastheni* or neurotic or neuros* or panic or phobi* or PND or “post-traumatic stress” or “posttraumatic stress*” or psychogenic or psychosocial* or psychosomatic or PTSD or “self injur*” or “self harm” or “self mutilat*” or psychosexual or (psychological and sexual and dysfunction*) or “social anxi*” or “socially anxi*” or somati* or somatoform or suicid* or parasuicid* or trichotillomani*)) Timespan: 2020-07-01 to 2024-02-28 Date Run: Wed Feb 28 2024 11:04:29 GMT+0000 (Greenwich Mean Time) Results: 6784

7: (#6 AND #3) Timespan: 2020-07-01 to 2024-02-28 Date Run: Wed Feb 28 2024 11:04:50 GMT+0000 (Greenwich Mean Time) Results: 283

8: ((TS=(“social prescribing")) OR (TS=((chang* or develop* or enhanc* or initiative* or intervention* or program* or mitigat* or address* or improv* or target*) SAME (“community condition*” or “conditions in the community” OR “living condition*” or “social condition*” OR “community circumstance**” or “living circumstance*” or “social circumstance*”))) OR (TS=("communit* connect*" or "community engagement*" or "community link*" or "community referral*" or "community intervention*" or "community wellbeing” or "social connect*" or "social engagement*" or "social link*" or "social referral*" or "social intervention*" or "social wellbeing” or "social and emotional wellbeing”))) Timespan: 2020-07-01 to 2024-02-28 Date Run: Wed Feb 28 2024 11:05:09 GMT+0000 (Greenwich Mean Time) Results: 714

9: (#8 AND #5) Timespan: 2020-07-01 to 2024-02-28 Date Run: Wed Feb 28 2024 11:05:29 GMT+0000 (Greenwich Mean Time) Results: 0

10: (#8 AND #7) Timespan: 2020-07-01 to 2024-02-28 Date Run: Wed Feb 28 2024 11:06:54 GMT+0000 (Greenwich Mean Time) Results: 1

11: ((TI=(housing or dwelling* or neighbourhood* or neighborhood* or “residential facilit*” or “independent living”)) OR (TI=(homeless*)) OR (AB=(“housing first” or “housing stability” or “housing instability” or “permanent housing” or “housing strateg*” or “housing polic*” or “housing project*” or “housing program*” or “housing

quality” or “social housing*”)) OR (TI=((autonomous or assisted or sheltered or support*) SAME accommodation)) OR (AB=(“autonomous housing” or “assisted housing” or “sheltered housing” or “autonomous accommodation” or “assisted accommodation” or “sheltered accommodation” or “autonomous dwelling*” or “assisted dwelling*” or “sheltered dwelling*”)) OR (TS=(clubhouse or “club house”)) OR (TI=(“autonomous living” or “independent living” or “assisted living” or “halfway house*” or “halfway home*” or “satellite hous*” or “satellite home*” or “community residences” or “group homes” or “community living” or “supervised apartments” or “therapeutic social clubs”)) OR (AB=(“halfway house*” or “halfway home*” or “satellite hous*” or “satellite home*” or “supervised apartments” or “therapeutic social clubs”)) OR (AB=((“autonomous living” or “independent living” or “assisted living” or “community residences” or “group homes” or “community living”) SAME (intervention* or program*))) OR (TI=((independ* or assist* or support* or secur* or sustain* or maint*) SAME (tenanc* or tenure*))) OR (AB=((independ* or assist* or support* or secur* or sustain* or maint*) SAME (tenanc* or tenure*))) OR TI=("neighbourhood* characteristic*") OR (TS=("neighbourhood* intervention*" or "neighborhood* intervention*" or "neighborhood* program*" or "neighbourhood* program*")) OR (TI=((environment* or housing or neighborhood* or neighbourhood*) SAME infrastructure)) OR (AB=((environment* or housing or neighborhood* or neighbourhood*) SAME infrastructure)) or (TI=(“built environment*” or “urban planning”))) Timespan: 2020-07-01 to 2024-02-28 Date Run: Wed Feb 28 2024 11:07:17 GMT+0000 (Greenwich Mean Time) Results: 1596

12: (#11 AND #5) Timespan: 2020-07-01 to 2024-02-28 Date Run: Wed Feb 28 2024 11:07:34 GMT+0000 (Greenwich Mean Time) Results: 0

13: (#11 AND #7) Timespan: 2020-07-01 to 2024-02-28 Date Run: Wed Feb 28 2024 11:07:50 GMT+0000 (Greenwich Mean Time) Results: 0

14: ((TI=(money or cash or finan* or savings)) OR (TI=(“economic status” or pensions or remuneration or salary or salaries or “personal financ*” or “financial* autonom*” or “financial* secur*” or “paid work” or “financial* insecur*” or loans or borrowing or budgeting or "dealing with money” or microcredit or microfinance or “social fund*” or “economic hardship*” or “financial hardship*” or “family income*” or “family tax credit*” or “universal credit”)) OR (AB=(“economic status” or pensions or remuneration or salary or salaries or “personal financ*” or “financial* autonom*” or “financial* secur*” or “paid work” or “financial* insecur*” or loans or borrowing or budgeting or "dealing with money” or microcredit or microfinance or “social fund*” or

“economic hardship*” or “financial hardship*” or “family income*” or “family tax credit*” or “universal credit”)) OR (TI=(poverty or debt or debts or “social welfare”)) OR (AB=(poverty SAME prevent*)) OR (AB=(“high poverty” or "out of poverty” or “food poverty” or “fuel poverty” or “food bank*” or “welfare benefit*”)) OR (AB=(“basic wage*” or “basic income*” or “minimum wage*” or “minimum income*” or “regular income*”)) OR (TI=(“basic wage*” or “basic income*” or “minimum wage*” or “minimum income*” or “regular income*”))) Timespan: 2020-07-01 to 2024-02-28 Date Run: Wed Feb 28 2024 11:08:05 GMT+0000 (Greenwich Mean Time) Results: 3577

15: (#5 and #14) Timespan: 2020-07-01 to 2024-02-28 Date Run: Wed Feb 28 2024 11:08:20 GMT+0000 (Greenwich Mean Time) Results: 1

Search Name: CCRT_Barnett_Family

Date Run: 28/02/2024 09:45:32

Comment:

ID	Search	Hits
#1	SR-DEPRESSN OR HS-DEPRESSN	35371
#2	MeSH descriptor: [Mental Disorders] this term only	5377
#3	MeSH descriptor: [Anxiety Disorders] explode all trees	10069
#4	MeSH descriptor: [Trichotillomania] this term only	96
#5	MeSH descriptor: [Feeding and Eating Disorders] this term only	1185
#6	MeSH descriptor: [Anorexia Nervosa] this term only	754
#7	MeSH descriptor: [Binge-Eating Disorder] this term only	482
#8	MeSH descriptor: [Bulimia Nervosa] this term only	362
#9	MeSH descriptor: [Mood Disorders] this term only	1127
#10	MeSH descriptor: [Depressive Disorder] explode all trees	16414
#11	MeSH descriptor: [Premenstrual Dysphoric Disorder] this term only	51
#12	MeSH descriptor: [Reactive Attachment Disorder] this term only	24
#13	MeSH descriptor: [Sexual Dysfunctions, Psychological] this term only	504

#14 MeSH descriptor: [Somatoform Disorders] explode all trees 945

#15 MeSH descriptor: [Munchausen Syndrome] explode all trees 2

#16 MeSH descriptor: [Trauma and Stressor Related Disorders] explode all trees 4508

#17 MeSH descriptor: [Trauma and Stressor Related Disorders] explode all trees 4508

#18 MeSH descriptor: [Impulsive Behavior] explode all trees 1672

#19 MeSH descriptor: [Hoarding] this term only 7

#20 MeSH descriptor: [Self-Injurious Behavior] explode all trees 2427

#21 ("anorexia nervosa" or "binge eating disorder" or bulimia or "eating disorder" or automutilation or "suicidal behaviour" or "self poisoning" or "suicidal ideation" or suicide or "suicide attempt" or depression or "agitated depression" or "atypical depression" or "chronic depression" or "depressive psychosis" or dysphoria or dysthymia or "endogenous depression" or involutional or "late life depression" or "major depression" or melancholia or "minor depression" or "mourning syndrome" or "organic depression" or "perinatal depression" or "antenatal depression" or "postnatal depression" or "post-stroke depression" or "postoperative depression" or "premenstrual dysphoric disorder" or "reactive depression" or "recurrent brief depression" or "seasonal affective disorder" or "treatment resistant depression" or neurosis or "affective neurosis" or "anxiety neurosis" or dysthymia or hysteria or neurasthenia or psychasthenia or "adjustment disorder" or "anxiety disorder" or "acute stress disorder" or "generalized anxiety disorder" or panic or "posttraumatic stress disorder" or "separation anxiety" or "obsessive compulsive disorder" or compulsion or obsession or phobia or agoraphobia or claustrophobia or neophobia or "social phobia" or "somatoform disorder" or "body dysmorphic disorder" or "conversion disorder" or "delusional pregnancy" or hypochondriasis or "masked depression" or "psychogenic pain" or somatization or "mood disorder" or "affective neurosis" or "affective psychosis" or "blunted affect" or "major affective disorder" or "minor affective disorder" or "munchausen syndrome by proxy" or "munchausen syndrome" or "psychosexual disorder" or kleptomania or "trichotillomania" or "emotional disorder"):kw 63877

#22 ("MENTAL DISORDERS" or anhedonia or neurosis or "ACUTE STRESS DISORDER" or "adjustment disorders" or "attachment disorders" or "disinhibited social engagement disorder" or "posttraumatic stress disorder" or "complex ptsd" or desnos or "acute stress disorder" or "post-traumatic stress" or "traumatic neurosis" or "emotional trauma" or "AFFECTIVE DISORDERS" or "disruptive mood dysregulation disorder" or "dysthymic disorder" or "seasonal affective disorder" or "major

depression” or “anaclitic depression” or “dysthymic disorder” or “endogenous depression” or “late life depression” or “postpartum depression” or “reactive depression” or “recurrent depression” or “treatment resistant depression” or “premenstrual dysphoric disorder” or “ANXIETY DISORDERS” or “generalized anxiety disorder” or “obsessive compulsive disorder” or “panic attack” or “panic disorder” or phobias or trichotillomania or acrophobia or agoraphobia or claustrophobia or ophidiophobia or “social phobia” or “separation anxiety disorder” or “EATING DISORDERS” or “anorexia nervosa” or “binge eating disorder” or bulimia or “SELF-INJURIOUS BEHAVIOR” or “self-destructive behavior” or “self-inflicted wounds” or “self-mutilation” or “self-poisoning” or suicide or “attempted suicide” or suicidality or “suicidal ideation” or “suicide prevention” or “SOMATOFORM DISORDERS” or “body dysmorphic disorder” or hypochondriasis or hysteria or neurasthenia or neurodermatitis or “somatization disorder” or “somatoform pain disorder” or “munchausen syndrome” or “munchausen syndrome by proxy” or (depression near/2 emotion) or "stress and trauma related disorders" or “mental health” or “mental health services” or “community mental health services” or “community counseling” or “community psychiatry”):kw 45016

#23 ("acute stress" or (adjustment next disorder*) or ADNOS or (affective next disorder*) or agoraphobi* or "anorexia nervosa" or anxiety or astheni* or (attachment next disorder*) or (binge next eat*) or binging or (body next dysmorphi*) or bulimi* or (combat next disorder*) or (obsessive next compulsi*) or depression or depressed or depressive or dyssomni* or dyspareunia* or dysphori* or dysthymi* or dystoni* or (eating next disorder*) or EDNOS or (emotional next trauma) or fear or (health next anxiety) or hoarding or hyperactivity or hypochondri* or hysteri* or (medically next unexplained) or malingering or MDD or (common next mental) or (mental* near/2 (health or well*)) or mood or moods or munchausen or MUPS or mutism or neurastheni* or neurotic or neuros* or panic or phobi* or PND or ((post-trauma* or posttrauma*) next stress*) or psychogenic or psychosomatic or PTSD or (self next (injur* or harm or mutilat*)) or psychosexual or (psychological near/3 sexual near/3 dysfunction*) or "social anxiety" or somati* or somatoform or suicid* or parasuicid* or trichotillomani*):ti,ab 196731

#24 (depress* NEAR (employment or employability or unemployment or "return to work" or vocation* or absenteeism or presenteeism or "job security" or "work related" or "work focussed")):ab241

#25 (#1 OR #2 OR #3 OR #4 OR #5 OR #6 OR #7 OR #8 OR #9 OR #10 OR #11 OR #12 OR #13 OR #14 OR #15 OR #16 OR #17 OR #18 OR #19 OR #20 OR #21 OR #22 OR #23 OR #24) 221261

#26 SR-SCIHZ OR HS-SCHIZ 941

#27 (bipolar or cyclothymi* or mania or manic or hypermani* or “rapid cycling” or (conversion next disorder*) or (dissociative next (amnesi* or fugue* or disorder*)) or "borderline state" or catatoni* or "character disorder" or delusion* or (capgras next syndrom*) or (diogenes next syndrom*) or depersonalization or depersonalisation or de-personalization or de-personalisation or (perceptual next disorder*) or (personality next disorder*) or BPD or paranoi* or psychiatr* or psychopathol* or psycho-pathol* or psychotic or psychosis* or psychoses* or psychotic* or schizo* or hebephreni* or “serious mental” or SMI):ti,ab,kw 69764

#28 MeSH descriptor: [Schizophrenia Spectrum and Other Psychotic Disorders] explode all trees 12178

#29 MeSH descriptor: [Personality Disorders] explode all trees 1828

#30 MeSH descriptor: [Bipolar and Related Disorders] explode all trees 3561

#31 (#26 OR #27 OR #28 OR #29 OR #30) 70436

#32 ("living arrangements" or "home environment" or "family conflict" or "family functioning" or "family relations" or "family reunification" or "intergenerational relations" or "marital conflict" or "marital relations" or divorce or divorced or "separated parent" or "single parent" or "living alone" or parenting or "parental involvement" or "parental role"):kw 4551

#33 ("child custody" or "joint custody" or "custodial care" or (child next visit*)):kw 100

#34 ((living next arrangement*) or (home next environment*) or (family and conflict*) or (family next function*) or (family next relations*) or "family reunification" or (intergenerational next relations*) or "marital conflict" or "marital relations" or divorce or divorced or "separated parent" or "single parent" or "living alone" or parenting or "parental involvement" or (parent* next role*)):ti 2235

#35 ((family or families or intergenerat* or inter-generat*) next (relation* or conflict or conflicts)):ti,ab 770

#36 ((sexual or intimate or partner or partners or marital) next (relation* or conflict or conflicts)):ti,ab 982

#37 ((develop* or enhanc* or initiative* or intervention* or program* or address* or improv* or promot* or target*) near/2 (relationship or relationships or relations)):ti,ab 2011

#38 ((carer or carers or partner or partners or relations or relationship or relationships or marital) next support*):ti,ab 288

#39 (child* near/2 (access or contact or custody or maintenance)):ti,ab 487

#40 (((care next proceeding*) or (family next (court or courts)) or "child removal" or fostercare or “foster care”) and (parent* or mother* or father*)):ti,ab 141

#41 parents:ti or (parent* next outcome*):ti,ab 3767

#42 (parent* next (mental health)):ti,ab 537

#43 (#32 OR #33 OR #34 OR #35 OR #36 OR #37 OR #38 OR #39 OR #40 OR #41 OR #42) 12865

#44 #25 AND #43 with Cochrane Library publication date Between Jul 2020 and Feb 2024, in Trials 1649

#45 #31 AND #43 with Cochrane Library publication date Between Jul 2020 and Feb 2022, in Trials 114

Search Name: CCRT_Barnett_Housing_CMD_SMI

Date Run: 28/02/2024 09:32:03

Comment:

ID	Search	Hits
#1	SR-DEPRESSN OR HS-DEPRESSN	35371
#2	MeSH descriptor: [Mental Disorders] this term only	5377
#3	MeSH descriptor: [Anxiety Disorders] explode all trees	10069
#4	MeSH descriptor: [Trichotillomania] this term only	96
#5	MeSH descriptor: [Feeding and Eating Disorders] this term only	1185
#6	MeSH descriptor: [Anorexia Nervosa] this term only	754
#7	MeSH descriptor: [Binge-Eating Disorder] this term only	482
#8	MeSH descriptor: [Bulimia Nervosa] this term only	362
#9	MeSH descriptor: [Mood Disorders] this term only	1127
#10	MeSH descriptor: [Depressive Disorder] explode all trees	16414
#11	MeSH descriptor: [Premenstrual Dysphoric Disorder] this term only	51
#12	MeSH descriptor: [Reactive Attachment Disorder] this term only	24
#13	MeSH descriptor: [Sexual Dysfunctions, Psychological] this term only	504

#14 MeSH descriptor: [Somatoform Disorders] explode all trees 945

#15 MeSH descriptor: [Munchausen Syndrome] explode all trees 2

#16 MeSH descriptor: [Trauma and Stressor Related Disorders] explode all trees 4508

#17 MeSH descriptor: [Trauma and Stressor Related Disorders] explode all trees 4508

#18 MeSH descriptor: [Impulsive Behavior] explode all trees 1672

#19 MeSH descriptor: [Hoarding] this term only 7

#20 MeSH descriptor: [Self-Injurious Behavior] explode all trees 2427

#21 ("anorexia nervosa" or "binge eating disorder" or bulimia or "eating disorder" or automutilation or "suicidal behaviour" or "self poisoning" or "suicidal ideation" or suicide or "suicide attempt" or depression or "agitated depression" or "atypical depression" or "chronic depression" or "depressive psychosis" or dysphoria or dysthymia or "endogenous depression" or involutional or "late life depression" or "major depression" or melancholia or "minor depression" or "mourning syndrome" or "organic depression" or "perinatal depression" or "antenatal depression" or "postnatal depression" or "post-stroke depression" or "postoperative depression" or "premenstrual dysphoric disorder" or "reactive depression" or "recurrent brief depression" or "seasonal affective disorder" or "treatment resistant depression" or neurosis or "affective neurosis" or "anxiety neurosis" or dysthymia or hysteria or neurasthenia or psychasthenia or "adjustment disorder" or "anxiety disorder" or "acute stress disorder" or "generalized anxiety disorder" or panic or "posttraumatic stress disorder" or "separation anxiety" or "obsessive compulsive disorder" or compulsion or obsession or phobia or agoraphobia or claustrophobia or neophobia or "social phobia" or "somatoform disorder" or "body dysmorphic disorder" or "conversion disorder" or "delusional pregnancy" or hypochondriasis or "masked depression" or "psychogenic pain" or somatization or "mood disorder" or "affective neurosis" or "affective psychosis" or "blunted affect" or "major affective disorder" or "minor affective disorder" or "munchausen syndrome by proxy" or "munchausen syndrome" or "psychosexual disorder" or kleptomania or "trichotillomania" or "emotional disorder"):kw 63877

#22 ("MENTAL DISORDERS" or anhedonia or neurosis or "ACUTE STRESS DISORDER" or "adjustment disorders" or "attachment disorders" or "disinhibited social engagement disorder" or "posttraumatic stress disorder" or "complex ptsd" or desnos or "acute stress disorder" or "post-traumatic stress" or "traumatic neurosis" or "emotional trauma" or "AFFECTIVE DISORDERS" or "disruptive mood dysregulation disorder" or "dysthymic disorder" or "seasonal affective disorder" or "major

depression” or “anaclitic depression” or “dysthymic disorder” or “endogenous depression” or “late life depression” or “postpartum depression” or “reactive depression” or “recurrent depression” or “treatment resistant depression” or “premenstrual dysphoric disorder” or “ANXIETY DISORDERS” or “generalized anxiety disorder” or “obsessive compulsive disorder” or “panic attack” or “panic disorder” or phobias or trichotillomania or acrophobia or agoraphobia or claustrophobia or ophidiophobia or “social phobia” or “separation anxiety disorder” or “EATING DISORDERS” or “anorexia nervosa” or “binge eating disorder” or bulimia or “SELF-INJURIOUS BEHAVIOR” or “self-destructive behavior” or “self-inflicted wounds” or “self-mutilation” or “self-poisoning” or suicide or “attempted suicide” or suicidality or “suicidal ideation” or “suicide prevention” or “SOMATOFORM DISORDERS” or “body dysmorphic disorder” or hypochondriasis or hysteria or neurasthenia or neurodermatitis or “somatization disorder” or “somatoform pain disorder” or “munchausen syndrome” or “munchausen syndrome by proxy” or (depression near/2 emotion) or "stress and trauma related disorders" or “mental health” or “mental health services” or “community mental health services” or “community counseling” or “community psychiatry”):kw 45016

#23 ("acute stress" or (adjustment next disorder*) or ADNOS or (affective next disorder*) or agoraphobi* or "anorexia nervosa" or anxiety or astheni* or (attachment next disorder*) or (binge next eat*) or binging or (body next dysmorphi*) or bulimi* or (combat next disorder*) or (obsessive next compulsi*) or depression or depressed or depressive or dyssomni* or dyspareunia* or dysphori* or dysthymi* or dystoni* or (eating next disorder*) or EDNOS or (emotional next trauma) or fear or (health next anxiety) or hoarding or hyperactivity or hypochondri* or hysteri* or (medically next unexplained) or malingering or MDD or (common next mental) or (mental* near/2 (health or well*)) or mood or moods or munchausen or MUPS or mutism or neurastheni* or neurotic or neuros* or panic or phobi* or PND or ((post-trauma* or posttrauma*) next stress*) or psychogenic or psychosomatic or PTSD or (self next (injur* or harm or mutilat*)) or psychosexual or (psychological near/3 sexual near/3 dysfunction*) or "social anxiety" or somati* or somatoform or suicid* or parasuicid* or trichotillomani*):ti,ab 196731

#24 (depress* NEAR (employment or employability or unemployment or "return to work" or vocation* or absenteeism or presenteeism or "job security" or "work related" or "work focussed")):ab241

#25 (#1 OR #2 OR #3 OR #4 OR #5 OR #6 OR #7 OR #8 OR #9 OR #10 OR #11 OR #12 OR #13 OR #14 OR #15 OR #16 OR #17 OR #18 OR #19 OR #20 OR #21 OR #22 OR #23 OR #24) 221261

#26 SR-SCIHZ OR HS-SCHIZ 941

#27 (bipolar or cyclothymi* or mania or manic or hypermani* or “rapid cycling” or (conversion next disorder*) or (dissociative next (amnesi* or fugue* or disorder*)) or "borderline state" or catatoni* or "character disorder" or delusion* or (capgras next syndrom*) or (diogenes next syndrom*) or depersonalization or depersonalisation or de-personalization or de-personalisation or (perceptual next disorder*) or (personality next disorder*) or BPD or paranoi* or psychiatr* or psychopathol* or psycho-pathol* or psychotic or psychosis* or psychoses* or psychotic* or schizo* or hebephreni* or “serious mental” or SMI):ti,ab,kw 69764

#28 MeSH descriptor: [Schizophrenia Spectrum and Other Psychotic Disorders] explode all trees 12178

#29 MeSH descriptor: [Personality Disorders] explode all trees 1828

#30 MeSH descriptor: [Bipolar and Related Disorders] explode all trees 3561

#31 (#26 OR #27 OR #28 OR #29 OR #30) 70436

#32 (housing or “assisted living” or “independent living” or “community living” “group homes” or “public housing” or “living arrangements” or “emergency shelter” or homeless* or deinstitutionalization or “halfway house” or “halfway houses” or shelters or “residential care institutions” or “residence characteristics”):kw 3713

#33 (“built environment” or “environmental planning” or “city planning” or “urban planning” or neighborhood* or neighbourhood* or “social environment” or “psychosocial environment” or “social environments” or “poverty areas”):kw 2292

#34 ((chang* or develop* or enhanc* or initiative* or intervention* or program* or mitigat* or address* or improv* or target*) near/3 (housing or neighbo*)):ti,ab580

#35 (dwelling* or housing):ti 2370

#36 homeless*:ti,ab,kw 1214

#37 (“housing first” or “permanent housing” or (housing near/2 (stability or instability))):ti,ab 255

#38 ((housing next (strateg* or polic* or project* or program* or quality)) or (new* next buil*) or “social housing”):ti,ab 126

#39 ((autonomous or assisted or sheltered or support* or public) near/3 (housing or accommodation or dwelling*)):ti,ab 467

#40 (((clubhouse or “club house”) next model*) or ((autonomous or independent or assisted) next living)):ti,ab,kw 1996

#41 (tenanc* or tenant* or tenure*):ti,ab,kw 206

#42 (((halfway or satellite) next (dwelling* or home* or house* or housing*)) or "community housing"):ti,ab,kw 63

#43 ((neighbo*) near/2 (characteristic* or intervention* or program*)):ti,ab 134

#44 ((neighbo* or housing) and infrastructure):ti,ab,kw 58

#45 neighbo*:ti or (neighbourhoods or neighborhoods):ti,ab,kw 968

#46 (#32 OR #33 OR #34 OR #35 OR #36 OR #37 OR #38 OR #39 OR #40 OR #41 OR #42 OR #43 OR #44 OR #45) 9447

#47 #25 AND #46 with Cochrane Library publication date Between Jul 2020 and Feb 2024, in Trials 726

#48 #31 AND #46 with Cochrane Library publication date Between Jul 2020 and Feb 2024, in Trials 155

Search Name: CCRT_Barnett_Linking

Date Run: 28/02/2024 11:12:33

Comment:

ID Search Hits

#1 SR-DEPRESSN OR HS-DEPRESSN 35371

#2 MeSH descriptor: [Mental Disorders] this term only 5377

#3 MeSH descriptor: [Anxiety Disorders] explode all trees 10069

#4 MeSH descriptor: [Trichotillomania] this term only 96

#5 MeSH descriptor: [Feeding and Eating Disorders] this term only 1185

#6 MeSH descriptor: [Anorexia Nervosa] this term only 754

#7 MeSH descriptor: [Binge-Eating Disorder] this term only 482

#8 MeSH descriptor: [Bulimia Nervosa] this term only 362

#9 MeSH descriptor: [Mood Disorders] this term only 1127

#10 MeSH descriptor: [Depressive Disorder] explode all trees 16414

#11 MeSH descriptor: [Premenstrual Dysphoric Disorder] this term only 51

#12 MeSH descriptor: [Reactive Attachment Disorder] this term only 24

#13 MeSH descriptor: [Sexual Dysfunctions, Psychological] this term only 504

#14 MeSH descriptor: [Somatoform Disorders] explode all trees 945

#15 MeSH descriptor: [Munchausen Syndrome] explode all trees 2

#16 MeSH descriptor: [Trauma and Stressor Related Disorders] explode all trees 4508

#17 MeSH descriptor: [Trauma and Stressor Related Disorders] explode all trees 4508

#18 MeSH descriptor: [Impulsive Behavior] explode all trees 1672

#19 MeSH descriptor: [Hoarding] this term only 7

#20 MeSH descriptor: [Self-Injurious Behavior] explode all trees 2427

#21 (“anorexia nervosa” or “binge eating disorder” or bulimia or “eating disorder” or automutilation or “suicidal behaviour” or “self poisoning” or “suicidal ideation” or suicide or “suicide attempt” or depression or “agitated depression” or “atypical depression” or “chronic depression” or “depressive psychosis” or dysphoria or dysthymia or “endogenous depression” or involutional or “late life depression” or “major depression” or melancholia or “minor depression” or “mourning syndrome” or “organic depression” or “perinatal depression” or “antenatal depression” or “postnatal depression” or “post-stroke depression” or “postoperative depression” or “premenstrual dysphoric disorder” or “reactive depression” or “recurrent brief depression” or “seasonal affective disorder” or “treatment resistant depression” or neurosis or “affective neurosis” or “anxiety neurosis” or dysthymia or hysteria or neurasthenia or psychasthenia or “adjustment disorder” or “anxiety disorder” or “acute stress disorder” or “generalized anxiety disorder” or panic or “posttraumatic stress disorder” or “separation anxiety” or “obsessive compulsive disorder” or compulsion or obsession or phobia or agoraphobia or claustrophobia or neophobia or “social phobia” or “somatoform disorder” or “body dysmorphic disorder” or “conversion disorder” or “delusional pregnancy” or hypochondriasis or “masked depression” or “psychogenic pain” or somatization or “mood disorder” or “affective neurosis” or “affective psychosis” or “blunted affect” or “major affective disorder” or “minor affective disorder” or “munchausen syndrome by proxy” or “munchausen syndrome” or “psychosexual disorder” or kleptomania or “trichotillomania” or “emotional disorder”):kw 63877

#22 (“MENTAL DISORDERS” or anhedonia or neurosis or “ACUTE STRESS DISORDER” or “adjustment disorders” or “attachment disorders” or “disinhibited social engagement disorder” or “posttraumatic stress disorder” or “complex ptsd” or desnos or “acute stress disorder” or “post-traumatic stress” or “traumatic neurosis” or

“emotional trauma” or “AFFECTIVE DISORDERS” or “disruptive mood dysregulation disorder” or “dysthymic disorder” or “seasonal affective disorder” or “major depression” or “anaclitic depression” or “dysthymic disorder” or “endogenous depression” or “late life depression” or “postpartum depression” or “reactive depression” or “recurrent depression” or “treatment resistant depression” or “premenstrual dysphoric disorder” or “ANXIETY DISORDERS” or “generalized anxiety disorder” or “obsessive compulsive disorder” or “panic attack” or “panic disorder” or phobias or trichotillomania or acrophobia or agoraphobia or claustrophobia or ophidiophobia or “social phobia” or “separation anxiety disorder” or “EATING DISORDERS” or “anorexia nervosa” or “binge eating disorder” or bulimia or “SELF-INJURIOUS BEHAVIOR” or “self-destructive behavior” or “self-inflicted wounds” or “self-mutilation” or “self-poisoning” or suicide or “attempted suicide” or suicidality or “suicidal ideation” or “suicide prevention” or “SOMATOFORM DISORDERS” or “body dysmorphic disorder” or hypochondriasis or hysteria or neurasthenia or neurodermatitis or “somatization disorder” or “somatoform pain disorder” or “munchausen syndrome” or “munchausen syndrome by proxy” or (depression near/2 emotion) or "stress and trauma related disorders" or “mental health” or “mental health services” or “community mental health services” or “community counseling” or “community psychiatry”):kw 45016

#23 ("acute stress" or (adjustment next disorder*) or ADNOS or (affective next disorder*) or agoraphobi* or "anorexia nervosa" or anxiety or astheni* or (attachment next disorder*) or (binge next eat*) or binging or (body next dysmorphi*) or bulimi* or (combat next disorder*) or (obsessive next compulsi*) or depression or depressed or depressive or dyssomni* or dyspareunia* or dysphori* or dysthymi* or dystoni* or (eating next disorder*) or EDNOS or (emotional next trauma) or fear or (health next anxiety) or hoarding or hyperactivity or hypochondri* or hysteri* or (medically next unexplained) or malingering or MDD or (common next mental) or (mental* near/2 (health or well*)) or mood or moods or munchausen or MUPS or mutism or neurastheni* or neurotic or neuros* or panic or phobi* or PND or ((post-trauma* or posttrauma*) next stress*) or psychogenic or psychosomatic or PTSD or (self next (injur* or harm or mutilat*)) or psychosexual or (psychological near/3 sexual near/3 dysfunction*) or "social anxiety" or somati* or somatoform or suicid* or parasuicid* or trichotillomani*):ti,ab 196731

#24 (depress* NEAR (employment or employability or unemployment or "return to work" or vocation* or absenteeism or presenteeism or "job security" or "work related" or "work focussed")):ab241

#25 (#1 OR #2 OR #3 OR #4 OR #5 OR #6 OR #7 OR #8 OR #9 OR #10 OR #11 OR #12 OR #13 OR #14 OR #15 OR #16 OR #17 OR #18 OR #19 OR #20 OR #21 OR #22 OR #23 OR #24) 221261

#26 SR-SCIHZ OR HS-SCHIZ 941

#27 (bipolar or cyclothymi* or mania or manic or hypermani* or “rapid cycling” or (conversion next disorder*) or (dissociative next (amnesi* or fugue* or disorder*)) or "borderline state" or catatoni* or "character disorder" or delusion* or (capgras next syndrom*) or (diogenes next syndrom*) or depersonalization or depersonalisation or de-personalization or de-personalisation or (perceptual next disorder*) or (personality next disorder*) or BPD or paranoi* or psychiatr* or psychopathol* or psycho-pathol* or psychotic or psychosis* or psychoses* or psychotic* or schizo* or hebephreni* or “serious mental” or SMI):ti,ab,kw 69764

#28 MeSH descriptor: [Schizophrenia Spectrum and Other Psychotic Disorders] explode all trees 12178

#29 MeSH descriptor: [Personality Disorders] explode all trees 1828

#30 MeSH descriptor: [Bipolar and Related Disorders] explode all trees 3561

#31 (#26 OR #27 OR #28 OR #29 OR #30) 70436

#32 ((chang* or develop* or enhanc* or initiative* or intervention* or program* or mitigat* or address* or improv* or target*) NEAR (community or living or social) NEAR (condition or conditions or circumstance*)):ti,ab,kw 815

#33 (social* NEAR/3 prescri*):ti,ab,kw 77

#34 (communit* next (connect* or engagement* or link* or referral* or intervention* or wellbeing)):ti,ab,kw 2341

#35 "sense of belonging":ti,ab,kw 83

#36 (#32 OR #33 OR #34 OR #35) 3251

#37 (#25 and #36) with Cochrane Library publication date Between Jul 2020 and Feb 2024, in Trials268

#38 (#31 and #36) with Cochrane Library publication date Between Jul 2020 and Feb 2024, in Trials53

Search Name: **CCRT_Barnett_Money**

Date Run: 28/02/2024 09:23:07

Comment:

ID Search Hits

#1 SR-DEPRESSN OR HS-DEPRESSN 35371

#2 MeSH descriptor: [Mental Disorders] this term only 5377

#3 MeSH descriptor: [Anxiety Disorders] explode all trees 10069

#4 MeSH descriptor: [Trichotillomania] this term only 96

#5 MeSH descriptor: [Feeding and Eating Disorders] this term only 1185

#6 MeSH descriptor: [Anorexia Nervosa] this term only 754

#7 MeSH descriptor: [Binge-Eating Disorder] this term only 482

#8 MeSH descriptor: [Bulimia Nervosa] this term only 362

#9 MeSH descriptor: [Mood Disorders] this term only 1127

#10 MeSH descriptor: [Depressive Disorder] explode all trees 16414

#11 MeSH descriptor: [Premenstrual Dysphoric Disorder] this term only 51

#12 MeSH descriptor: [Reactive Attachment Disorder] this term only 24

#13 MeSH descriptor: [Sexual Dysfunctions, Psychological] this term only 504

#14 MeSH descriptor: [Somatoform Disorders] explode all trees 945

#15 MeSH descriptor: [Munchausen Syndrome] explode all trees 2

#16 MeSH descriptor: [Trauma and Stressor Related Disorders] explode all trees 4508

#17 MeSH descriptor: [Trauma and Stressor Related Disorders] explode all trees 4508

#18 MeSH descriptor: [Impulsive Behavior] explode all trees 1672

#19 MeSH descriptor: [Hoarding] this term only 7

#20 MeSH descriptor: [Self-Injurious Behavior] explode all trees 2427

#21 (“anorexia nervosa” or “binge eating disorder” or bulimia or “eating disorder” or automutilation or “suicidal behaviour” or “self poisoning” or “suicidal ideation” or suicide or “suicide attempt” or depression or “agitated depression” or “atypical depression” or “chronic depression” or “depressive psychosis” or dysphoria or dysthymia or “endogenous depression” or involutional or “late life depression” or “major depression” or melancholia or “minor depression” or “mourning syndrome” or “organic depression” or “perinatal depression” or “antenatal depression” or “postnatal depression” or “post-stroke depression” or “postoperative depression” or “premenstrual dysphoric disorder” or “reactive depression” or “recurrent brief

depression" or "seasonal affective disorder" or "treatment resistant depression" or neurosis or "affective neurosis" or "anxiety neurosis" or dysthymia or hysteria or neurasthenia or psychasthenia or "adjustment disorder" or "anxiety disorder" or "acute stress disorder" or "generalized anxiety disorder" or panic or "posttraumatic stress disorder" or "separation anxiety" or "obsessive compulsive disorder" or compulsion or obsession or phobia or agoraphobia or claustrophobia or neophobia or "social phobia" or "somatoform disorder" or "body dysmorphic disorder" or "conversion disorder" or "delusional pregnancy" or hypochondriasis or "masked depression" or "psychogenic pain" or somatization or "mood disorder" or "affective neurosis" or "affective psychosis" or "blunted affect" or "major affective disorder" or "minor affective disorder" or "munchausen syndrome by proxy" or "munchausen syndrome" or "psychosexual disorder" or kleptomania or "trichotillomania" or "emotional disorder"):kw 63877

#22 ("MENTAL DISORDERS" or anhedonia or neurosis or "ACUTE STRESS DISORDER" or "adjustment disorders" or "attachment disorders" or "disinhibited social engagement disorder" or "posttraumatic stress disorder" or "complex ptsd" or desnos or "acute stress disorder" or "post-traumatic stress" or "traumatic neurosis" or "emotional trauma" or "AFFECTIVE DISORDERS" or "disruptive mood dysregulation disorder" or "dysthymic disorder" or "seasonal affective disorder" or "major depression" or "anaclitic depression" or "dysthymic disorder" or "endogenous depression" or "late life depression" or "postpartum depression" or "reactive depression" or "recurrent depression" or "treatment resistant depression" or "premenstrual dysphoric disorder" or "ANXIETY DISORDERS" or "generalized anxiety disorder" or "obsessive compulsive disorder" or "panic attack" or "panic disorder" or phobias or trichotillomania or acrophobia or agoraphobia or claustrophobia or ophidiophobia or "social phobia" or "separation anxiety disorder" or "EATING DISORDERS" or "anorexia nervosa" or "binge eating disorder" or bulimia or "SELF-INJURIOUS BEHAVIOR" or "self-destructive behavior" or "self-inflicted wounds" or "self-mutilation" or "self-poisoning" or suicide or "attempted suicide" or suicidality or "suicidal ideation" or "suicide prevention" or "SOMATOFORM DISORDERS" or "body dysmorphic disorder" or hypochondriasis or hysteria or neurasthenia or neurodermatitis or "somatization disorder" or "somatoform pain disorder" or "munchausen syndrome" or "munchausen syndrome by proxy" or (depression near/2 emotion) or "stress and trauma related disorders" or "mental health" or "mental health services" or "community mental health services" or "community counseling" or "community psychiatry"):kw 45016

#23 ("acute stress" or (adjustment next disorder*) or ADNOS or (affective next disorder*) or agoraphobi* or "anorexia nervosa" or anxiety or astheni* or (attachment next disorder*) or (binge next eat*) or binging or (body next dysmorphi*) or bulimi* or (combat next disorder*) or (obsessive next compulsi*) or depression or depressed or

depressive or dyssomni* or dyspareunia* or dysphori* or dysthymi* or dystoni* or (eating next disorder*) or EDNOS or (emotional next trauma) or fear or (health next anxiety) or hoarding or hyperactivity or hypochondri* or hysteri* or (medically next unexplained) or malingering or MDD or (common next mental) or (mental* near/2 (health or well*)) or mood or moods or munchausen or MUPS or mutism or neurastheni* or neurotic or neuros* or panic or phobi* or PND or ((post-trauma* or posttrauma*) next stress*) or psychogenic or psychosomatic or PTSD or (self next (injur* or harm or mutilat*)) or psychosexual or (psychological near/3 sexual near/3 dysfunction*) or "social anxiety" or somati* or somatoform or suicid* or parasuicid* or trichotillomani*):ti,ab 196731

#24 (depress* NEAR (employment or employability or unemployment or "return to work" or vocation* or absenteeism or presenteeism or "job security" or "work related" or "work focussed")):ab241

#25 (#1 OR #2 OR #3 OR #4 OR #5 OR #6 OR #7 OR #8 OR #9 OR #10 OR #11 OR #12 OR #13 OR #14 OR #15 OR #16 OR #17 OR #18 OR #19 OR #20 OR #21 OR #22 OR #23 OR #24) 221261

#26 SR-SCIHZ OR HS-SCHIZ 941

#27 (bipolar or cyclothymi* or mania or manic or hypermani* or “rapid cycling” or (conversion next disorder*) or (dissociative next (amnesi* or fugue* or disorder*)) or "borderline state" or catatoni* or "character disorder" or delusion* or (capgras next syndrom*) or (diogenes next syndrom*) or depersonalization or depersonalisation or de-personalization or de-personalisation or (perceptual next disorder*) or (personality next disorder*) or BPD or paranoi* or psychiatr* or psychopathol* or psycho-pathol* or psychotic or psychosis* or psychoses* or psychotic* or schizo* or hebephreni* or “serious mental” or SMI):ti,ab,kw 69764

#28 MeSH descriptor: [Schizophrenia Spectrum and Other Psychotic Disorders] explode all trees 12178

#29 MeSH descriptor: [Personality Disorders] explode all trees 1828

#30 MeSH descriptor: [Bipolar and Related Disorders] explode all trees 3561

#31 (#26 OR #27 OR #28 OR #29 OR #30) 70436

#32 (money or remuneration or salary or salaries or socioeconomics):kw 1843

#33 ((personal near/2 financ*) or (income near/2 (family or household or personal or level)) or "financial management" or "fringe benefit" or bonuses or pension or pensions):kw 1839

#34 (disadvantaged or “social deprivation” or “human needs” or “basic needs” or “personal needs” or “social needs” or welfare):kw 2079

#35 (“economic status” or “socioeconomic status” or “economic security” or “financial strain” or (employee and (assistance or benefits)) or “health insurance” or “workers compensation” or Medicaid):kw 2662

#36 ((access* or improv* or manag* or supplement*) NEAR/2 (cash or money or finance or finances or financial or income or incomes or savings)):ti,ab 552

#37 (financ* near/2 (autonomy or autonomous or security or insecurity or insecurities)):ti,ab 71

#38 (loans or borrowing or budgeting or microcredit or microfinance or (social* next fund*) or “high poverty” or ((income* or wage*) near/2 supplement*)):ti,ab,kw 469

#39 ((address* or escap* or improv* or support* or target*) near/2 (deprived or deprivation or deprivations or poor or poverty)):ti,ab,kw 630

#40 (“food poverty” or “fuel poverty” or “food bank” or “food banks”):ti,ab,kw 38

#41 ((alleviat* or ease or easing or manag* or prevent* or reduc* or stop*) near/2 (poverty or “economic hardship” or “economic hardships” or “financial hardship” or “financial hardships”)):ti,ab142

#42 ((alleviat* or eas* or easing or manag* or prevent* or reduc* or relief or stop*) near/2 (debt or debts)):ti,ab 11

#43 ((basic or minimum) NEAR/3 (wage* or income*)):ti,ab,kw 55

#44 (“zero hours” or “paid work” or “paid employment” or "paid leave" or “family income” or “family incomes” or “tax credit” or “tax credits” or “welfare benefit” or “welfare benefits”):ti,ab,kw 783

#45 ("out of poverty" or "dealing with money"):ab 9

#46 (money or poverty or debt* or welfare):ti 577

#47 (#32 OR #33 OR #34 OR #35 OR #36 OR #37 OR #38 OR #39 OR #40 OR #41 OR #42 OR #43 OR #44 OR #45 OR #46) 10518

#48 #25 AND #47 with Cochrane Library publication date Between Jul 2020 and Feb 2024 707

#49 #31 AND #47 with Cochrane Library publication date Between Jul 2020 and Feb 2024 141

Search Name: **CCRT_Barnett_Rights**

Date Run: 28/02/2024 09:58:13

Comment:

ID Search Hits

#1 SR-DEPRESSN OR HS-DEPRESSN 35371

#2 MeSH descriptor: [Mental Disorders] this term only 5377

#3 MeSH descriptor: [Anxiety Disorders] explode all trees 10069

#4 MeSH descriptor: [Trichotillomania] this term only 96

#5 MeSH descriptor: [Feeding and Eating Disorders] this term only 1185

#6 MeSH descriptor: [Anorexia Nervosa] this term only 754

#7 MeSH descriptor: [Binge-Eating Disorder] this term only 482

#8 MeSH descriptor: [Bulimia Nervosa] this term only 362

#9 MeSH descriptor: [Mood Disorders] this term only 1127

#10 MeSH descriptor: [Depressive Disorder] explode all trees 16414

#11 MeSH descriptor: [Premenstrual Dysphoric Disorder] this term only 51

#12 MeSH descriptor: [Reactive Attachment Disorder] this term only 24

#13 MeSH descriptor: [Sexual Dysfunctions, Psychological] this term only 504

#14 MeSH descriptor: [Somatoform Disorders] explode all trees 945

#15 MeSH descriptor: [Munchausen Syndrome] explode all trees 2

#16 MeSH descriptor: [Trauma and Stressor Related Disorders] explode all trees 4508

#17 MeSH descriptor: [Trauma and Stressor Related Disorders] explode all trees 4508

#18 MeSH descriptor: [Impulsive Behavior] explode all trees 1672

#19 MeSH descriptor: [Hoarding] this term only 7

#20 MeSH descriptor: [Self-Injurious Behavior] explode all trees 2427

#21 (“anorexia nervosa” or “binge eating disorder” or bulimia or “eating disorder” or automutilation or “suicidal behaviour” or “self poisoning” or “suicidal ideation” or

suicide or “suicide attempt” or depression or “agitated depression” or “atypical depression” or “chronic depression” or “depressive psychosis” or dysphoria or dysthymia or “endogenous depression” or involutional or “late life depression” or “major depression” or melancholia or “minor depression” or “mourning syndrome” or “organic depression” or “perinatal depression” or “antenatal depression” or “postnatal depression” or “post-stroke depression” or “postoperative depression” or “premenstrual dysphoric disorder” or “reactive depression” or “recurrent brief depression” or “seasonal affective disorder” or “treatment resistant depression” or neurosis or “affective neurosis” or “anxiety neurosis” or dysthymia or hysteria or neurasthenia or psychasthenia or “adjustment disorder” or “anxiety disorder” or “acute stress disorder” or “generalized anxiety disorder” or panic or “posttraumatic stress disorder” or “separation anxiety” or “obsessive compulsive disorder” or compulsion or obsession or phobia or agoraphobia or claustrophobia or neophobia or “social phobia” or “somatoform disorder” or “body dysmorphic disorder” or “conversion disorder” or “delusional pregnancy” or hypochondriasis or “masked depression” or “psychogenic pain” or somatization or “mood disorder” or “affective neurosis” or “affective psychosis” or “blunted affect” or “major affective disorder” or “minor affective disorder” or “munchausen syndrome by proxy” or “munchausen syndrome” or “psychosexual disorder” or kleptomania or “trichotillomania” or “emotional disorder”):kw 63877

#22 (“MENTAL DISORDERS” or anhedonia or neurosis or “ACUTE STRESS DISORDER” or “adjustment disorders” or “attachment disorders” or “disinhibited social engagement disorder” or “posttraumatic stress disorder” or “complex ptsd” or desnos or “acute stress disorder” or “post-traumatic stress” or “traumatic neurosis” or “emotional trauma” or “AFFECTIVE DISORDERS” or “disruptive mood dysregulation disorder” or “dysthymic disorder” or “seasonal affective disorder” or “major depression” or “anaclitic depression” or “dysthymic disorder” or “endogenous depression” or “late life depression” or “postpartum depression” or “reactive depression” or “recurrent depression” or “treatment resistant depression” or “premenstrual dysphoric disorder” or “ANXIETY DISORDERS” or “generalized anxiety disorder” or “obsessive compulsive disorder” or “panic attack” or “panic disorder” or phobias or trichotillomania or acrophobia or agoraphobia or claustrophobia or ophidiophobia or “social phobia” or “separation anxiety disorder” or “EATING DISORDERS” or “anorexia nervosa” or “binge eating disorder” or bulimia or “SELF-INJURIOUS BEHAVIOR” or “self-destructive behavior” or “self-inflicted wounds” or “self-mutilation” or “self-poisoning” or suicide or “attempted suicide” or suicidality or “suicidal ideation” or “suicide prevention” or “SOMATOFORM DISORDERS” or “body dysmorphic disorder” or hypochondriasis or hysteria or neurasthenia or neurodermatitis or “somatization disorder” or “somatoform pain disorder” or “munchausen syndrome” or “munchausen syndrome by proxy” or (depression near/2

emotion) or "stress and trauma related disorders" or “mental health” or “mental health services” or “community mental health services” or “community counseling” or “community psychiatry”):kw 45016

#23 ("acute stress" or (adjustment next disorder*) or ADNOS or (affective next disorder*) or agoraphobi* or "anorexia nervosa" or anxiety or astheni* or (attachment next disorder*) or (binge next eat*) or binging or (body next dysmorphi*) or bulimi* or (combat next disorder*) or (obsessive next compulsi*) or depression or depressed or depressive or dyssomni* or dyspareunia* or dysphori* or dysthymi* or dystoni* or (eating next disorder*) or EDNOS or (emotional next trauma) or fear or (health next anxiety) or hoarding or hyperactivity or hypochondri* or hysteri* or (medically next unexplained) or malingering or MDD or (common next mental) or (mental* near/2 (health or well*)) or mood or moods or munchausen or MUPS or mutism or neurastheni* or neurotic or neuros* or panic or phobi* or PND or ((post-trauma* or posttrauma*) next stress*) or psychogenic or psychosomatic or PTSD or (self next (injur* or harm or mutilat*)) or psychosexual or (psychological near/3 sexual near/3 dysfunction*) or "social anxiety" or somati* or somatoform or suicid* or parasuicid* or trichotillomani*):ti,ab 196731

#24 (depress* NEAR (employment or employability or unemployment or "return to work" or vocation* or absenteeism or presenteeism or "job security" or "work related" or "work focussed")):ab241

#25 (#1 OR #2 OR #3 OR #4 OR #5 OR #6 OR #7 OR #8 OR #9 OR #10 OR #11 OR #12 OR #13 OR #14 OR #15 OR #16 OR #17 OR #18 OR #19 OR #20 OR #21 OR #22 OR #23 OR #24) 221261

#26 SR-SCIHZ OR HS-SCHIZ 941

#27 (bipolar or cyclothymi* or mania or manic or hypermani* or “rapid cycling” or (conversion next disorder*) or (dissociative next (amnesi* or fugue* or disorder*)) or "borderline state" or catatoni* or "character disorder" or delusion* or (capgras next syndrom*) or (diogenes next syndrom*) or depersonalization or depersonalisation or de-personalization or de-personalisation or (perceptual next disorder*) or (personality next disorder*) or BPD or paranoi* or psychiatr* or psychopathol* or psycho-pathol* or psychotic or psychosis* or psychoses* or psychotic* or schizo* or hebephreni* or “serious mental” or SMI):ti,ab,kw 69764

#28 MeSH descriptor: [Schizophrenia Spectrum and Other Psychotic Disorders] explode all trees 12178

#29 MeSH descriptor: [Personality Disorders] explode all trees 1828

#30 MeSH descriptor: [Bipolar and Related Disorders] explode all trees 3561

#31 (#26 OR #27 OR #28 OR #29 OR #30) 70436

#32 rights:kw,ti 165

#33 (freedoms or citizenship):ti,ab,kw 261

#34 (“human rights” or “civil rights” or “reproductive rights” or ((women* or woman*) next rights)):ti,ab,kw 215

#35 ((citizen* or civil* or human* or legal or social or vote or voting) near/2 (right or rights)):ti,ab 222

#36 (“social justice” or “social protection” or “equal protection” or “personal autonomy”):ti,ab,kw 600

#37 (“public policy” or “public policies” or “government policy” or “policy making”):kw,ti 296

#38 ((government* or public or social) next polic*):ti,kw 206

#39 ((equity or equalit* or inequit* or inequalit* or dispar*) near focus*):ti,ab 107

#40 ("Social Determinants of Health" or "health disparities"):ti,kw 464

#41 ((social or societ* or communit* or neighbo*) near/3 (equit* or inequit* or inequalit* or dispar*)):ti,ab,kw 318

#42 (“digital divide” or “information literacy”):ti,ab,kw 90

#43 ((accessib* or "access to") near (computer* or internet)):ti,kw 32

#44 (digital* near/2 (inclusi* or exclusi* or divide* or disparit* or equit* or inequit* or inequalit*)):ti,ab,kw 309

#45 (#32 OR #33 OR #34 OR #35 OR #36 OR #37 OR #38 OR #39 OR #40 OR #41 OR #42 OR #43 OR #44) 2718

#46 (#25 and #45) 534

#47 CINAHL:AN 29651

#48 (#46 NOT #47) with Cochrane Library publication date Between Jul 2020 and Feb 2024, in Trials211

#49 (#31 and #45) 144

#50 (#49 NOT #47) with Cochrane Library publication date Between Jul 2020 and Feb 2024, in Trials36

Search Name: **CCRT_Barnett_Social Isolation**

Date Run: 28/02/2024 10:47:37

Comment:

ID Search Hits

#1 SR-DEPRESSN OR HS-DEPRESSN 35371

#2 MeSH descriptor: [Mental Disorders] this term only 5377

#3 MeSH descriptor: [Anxiety Disorders] explode all trees 10069

#4 MeSH descriptor: [Trichotillomania] this term only 96

#5 MeSH descriptor: [Feeding and Eating Disorders] this term only 1185

#6 MeSH descriptor: [Anorexia Nervosa] this term only 754

#7 MeSH descriptor: [Binge-Eating Disorder] this term only 482

#8 MeSH descriptor: [Bulimia Nervosa] this term only 362

#9 MeSH descriptor: [Mood Disorders] this term only 1127

#10 MeSH descriptor: [Depressive Disorder] explode all trees 16414

#11 MeSH descriptor: [Premenstrual Dysphoric Disorder] this term only 51

#12 MeSH descriptor: [Reactive Attachment Disorder] this term only 24

#13 MeSH descriptor: [Sexual Dysfunctions, Psychological] this term only 504

#14 MeSH descriptor: [Somatoform Disorders] explode all trees 945

#15 MeSH descriptor: [Munchausen Syndrome] explode all trees 2

#16 MeSH descriptor: [Trauma and Stressor Related Disorders] explode all trees 4508

#17 MeSH descriptor: [Trauma and Stressor Related Disorders] explode all trees 4508

#18 MeSH descriptor: [Impulsive Behavior] explode all trees 1672

#19 MeSH descriptor: [Hoarding] this term only 7

#20 MeSH descriptor: [Self-Injurious Behavior] explode all trees 2427

#21 (“anorexia nervosa” or “binge eating disorder” or bulimia or “eating disorder” or automutilation or “suicidal behaviour” or “self poisoning” or “suicidal ideation” or

suicide or “suicide attempt” or depression or “agitated depression” or “atypical depression” or “chronic depression” or “depressive psychosis” or dysphoria or dysthymia or “endogenous depression” or involutional or “late life depression” or “major depression” or melancholia or “minor depression” or “mourning syndrome” or “organic depression” or “perinatal depression” or “antenatal depression” or “postnatal depression” or “post-stroke depression” or “postoperative depression” or “premenstrual dysphoric disorder” or “reactive depression” or “recurrent brief depression” or “seasonal affective disorder” or “treatment resistant depression” or neurosis or “affective neurosis” or “anxiety neurosis” or dysthymia or hysteria or neurasthenia or psychasthenia or “adjustment disorder” or “anxiety disorder” or “acute stress disorder” or “generalized anxiety disorder” or panic or “posttraumatic stress disorder” or “separation anxiety” or “obsessive compulsive disorder” or compulsion or obsession or phobia or agoraphobia or claustrophobia or neophobia or “social phobia” or “somatoform disorder” or “body dysmorphic disorder” or “conversion disorder” or “delusional pregnancy” or hypochondriasis or “masked depression” or “psychogenic pain” or somatization or “mood disorder” or “affective neurosis” or “affective psychosis” or “blunted affect” or “major affective disorder” or “minor affective disorder” or “munchausen syndrome by proxy” or “munchausen syndrome” or “psychosexual disorder” or kleptomania or “trichotillomania” or “emotional disorder”):kw 63877

#22 (“MENTAL DISORDERS” or anhedonia or neurosis or “ACUTE STRESS DISORDER” or “adjustment disorders” or “attachment disorders” or “disinhibited social engagement disorder” or “posttraumatic stress disorder” or “complex ptsd” or desnos or “acute stress disorder” or “post-traumatic stress” or “traumatic neurosis” or “emotional trauma” or “AFFECTIVE DISORDERS” or “disruptive mood dysregulation disorder” or “dysthymic disorder” or “seasonal affective disorder” or “major depression” or “anaclitic depression” or “dysthymic disorder” or “endogenous depression” or “late life depression” or “postpartum depression” or “reactive depression” or “recurrent depression” or “treatment resistant depression” or “premenstrual dysphoric disorder” or “ANXIETY DISORDERS” or “generalized anxiety disorder” or “obsessive compulsive disorder” or “panic attack” or “panic disorder” or phobias or trichotillomania or acrophobia or agoraphobia or claustrophobia or ophidiophobia or “social phobia” or “separation anxiety disorder” or “EATING DISORDERS” or “anorexia nervosa” or “binge eating disorder” or bulimia or “SELF-INJURIOUS BEHAVIOR” or “self-destructive behavior” or “self-inflicted wounds” or “self-mutilation” or “self-poisoning” or suicide or “attempted suicide” or suicidality or “suicidal ideation” or “suicide prevention” or “SOMATOFORM DISORDERS” or “body dysmorphic disorder” or hypochondriasis or hysteria or neurasthenia or neurodermatitis or “somatization disorder” or “somatoform pain disorder” or “munchausen syndrome” or “munchausen syndrome by proxy” or (depression near/2

emotion) or "stress and trauma related disorders" or “mental health” or “mental health services” or “community mental health services” or “community counseling” or “community psychiatry”):kw 45016

#23 ("acute stress" or (adjustment next disorder*) or ADNOS or (affective next disorder*) or agoraphobi* or "anorexia nervosa" or anxiety or astheni* or (attachment next disorder*) or (binge next eat*) or binging or (body next dysmorphi*) or bulimi* or (combat next disorder*) or (obsessive next compulsi*) or depression or depressed or depressive or dyssomni* or dyspareunia* or dysphori* or dysthymi* or dystoni* or (eating next disorder*) or EDNOS or (emotional next trauma) or fear or (health next anxiety) or hoarding or hyperactivity or hypochondri* or hysteri* or (medically next unexplained) or malingering or MDD or (common next mental) or (mental* near/2 (health or well*)) or mood or moods or munchausen or MUPS or mutism or neurastheni* or neurotic or neuros* or panic or phobi* or PND or ((post-trauma* or posttrauma*) next stress*) or psychogenic or psychosomatic or PTSD or (self next (injur* or harm or mutilat*)) or psychosexual or (psychological near/3 sexual near/3 dysfunction*) or "social anxiety" or somati* or somatoform or suicid* or parasuicid* or trichotillomani*):ti,ab 196731

#24 (depress* NEAR (employment or employability or unemployment or "return to work" or vocation* or absenteeism or presenteeism or "job security" or "work related" or "work focussed")):ab241

#25 (#1 OR #2 OR #3 OR #4 OR #5 OR #6 OR #7 OR #8 OR #9 OR #10 OR #11 OR #12 OR #13 OR #14 OR #15 OR #16 OR #17 OR #18 OR #19 OR #20 OR #21 OR #22 OR #23 OR #24) 221261

#26 SR-SCIHZ OR HS-SCHIZ 941

#27 (bipolar or cyclothymi* or mania or manic or hypermani* or “rapid cycling” or (conversion next disorder*) or (dissociative next (amnesi* or fugue* or disorder*)) or "borderline state" or catatoni* or "character disorder" or delusion* or (capgras next syndrom*) or (diogenes next syndrom*) or depersonalization or depersonalisation or de-personalization or de-personalisation or (perceptual next disorder*) or (personality next disorder*) or BPD or paranoi* or psychiatr* or psychopathol* or psycho-pathol* or psychotic or psychosis* or psychoses* or psychotic* or schizo* or hebephreni* or “serious mental” or SMI):ti,ab,kw 69764

#28 MeSH descriptor: [Schizophrenia Spectrum and Other Psychotic Disorders] explode all trees 12178

#29 MeSH descriptor: [Personality Disorders] explode all trees 1828

#30 MeSH descriptor: [Bipolar and Related Disorders] explode all trees 3561

#31	(#26 OR #27 OR #28 OR #29 OR #30)	70436

#32	(loneliness or lonely):ti,ab,kw	1395

#33	((social* or societ* or communit*) near (isolated or isolation)):ti,ab,kw1724

#34	((alleviat* or ease or manag* or mitigat* or prevent* or overcom* or reduc* or stop*) near/2 (isolation or isolated)):ti,ab,kw	324

#35	((address* or enhanc* or improv* or increas* or promot* or target*) near/2 (inclusion or inclusivity)):ti,ab,kw	673

#36	(#32 or #33 or #34 or #35)	3739

#37	(#25 and #36) with Cochrane Library publication date Between Jul 2020 and Feb 2024, in Trials916

#38	(#31 and #36) with Cochrane Library publication date Between Jul 2020 and Feb 2024, in Trials173

**Search Name:	CCRT_Barnett_Social Participation CMD**

Date Run:	28/02/2024 11:04:53

Comment:

ID	Search	Hits

#1	SR-DEPRESSN OR HS-DEPRESSN	35371

#2	MeSH descriptor: [Mental Disorders] this term only	5377

#3	MeSH descriptor: [Anxiety Disorders] explode all trees	10069

#4	MeSH descriptor: [Trichotillomania] this term only	96

#5	MeSH descriptor: [Feeding and Eating Disorders] this term only1185

#6	MeSH descriptor: [Anorexia Nervosa] this term only	754

#7	MeSH descriptor: [Binge-Eating Disorder] this term only	482

#8	MeSH descriptor: [Bulimia Nervosa] this term only	362

#9	MeSH descriptor: [Mood Disorders] this term only	1127

#10	MeSH descriptor: [Depressive Disorder] explode all trees	16414

#11	MeSH descriptor: [Premenstrual Dysphoric Disorder] this term only	51

#12 MeSH descriptor: [Reactive Attachment Disorder] this term only 24

#13 MeSH descriptor: [Sexual Dysfunctions, Psychological] this term only 504

#14 MeSH descriptor: [Somatoform Disorders] explode all trees 945

#15 MeSH descriptor: [Munchausen Syndrome] explode all trees 2

#16 MeSH descriptor: [Trauma and Stressor Related Disorders] explode all trees 4508

#17 MeSH descriptor: [Trauma and Stressor Related Disorders] explode all trees 4508

#18 MeSH descriptor: [Impulsive Behavior] explode all trees 1672

#19 MeSH descriptor: [Hoarding] this term only 7

#20 MeSH descriptor: [Self-Injurious Behavior] explode all trees 2427

#21 ("anorexia nervosa" or "binge eating disorder" or bulimia or "eating disorder" or automutilation or "suicidal behaviour" or "self poisoning" or "suicidal ideation" or suicide or "suicide attempt" or depression or "agitated depression" or "atypical depression" or "chronic depression" or "depressive psychosis" or dysphoria or dysthymia or "endogenous depression" or involutional or "late life depression" or "major depression" or melancholia or "minor depression" or "mourning syndrome" or "organic depression" or "perinatal depression" or "antenatal depression" or "postnatal depression" or "post-stroke depression" or "postoperative depression" or "premenstrual dysphoric disorder" or "reactive depression" or "recurrent brief depression" or "seasonal affective disorder" or "treatment resistant depression" or neurosis or "affective neurosis" or "anxiety neurosis" or dysthymia or hysteria or neurasthenia or psychasthenia or "adjustment disorder" or "anxiety disorder" or "acute stress disorder" or "generalized anxiety disorder" or panic or "posttraumatic stress disorder" or "separation anxiety" or "obsessive compulsive disorder" or compulsion or obsession or phobia or agoraphobia or claustrophobia or neophobia or "social phobia" or "somatoform disorder" or "body dysmorphic disorder" or "conversion disorder" or "delusional pregnancy" or hypochondriasis or "masked depression" or "psychogenic pain" or somatization or "mood disorder" or "affective neurosis" or "affective psychosis" or "blunted affect" or "major affective disorder" or "minor affective disorder" or "munchausen syndrome by proxy" or "munchausen syndrome" or "psychosexual disorder" or kleptomania or "trichotillomania" or "emotional disorder"):kw 63877

#22 ("MENTAL DISORDERS" or anhedonia or neurosis or "ACUTE STRESS DISORDER" or "adjustment disorders" or "attachment disorders" or "disinhibited social engagement disorder" or "posttraumatic stress disorder" or "complex ptsd" or

desnos or “acute stress disorder” or “post-traumatic stress” or “traumatic neurosis” or “emotional trauma” or “AFFECTIVE DISORDERS” or “disruptive mood dysregulation disorder” or “dysthymic disorder” or “seasonal affective disorder” or “major depression” or “anaclitic depression” or “dysthymic disorder” or “endogenous depression” or “late life depression” or “postpartum depression” or “reactive depression” or “recurrent depression” or “treatment resistant depression” or “premenstrual dysphoric disorder” or “ANXIETY DISORDERS” or “generalized anxiety disorder” or “obsessive compulsive disorder” or “panic attack” or “panic disorder” or phobias or trichotillomania or acrophobia or agoraphobia or claustrophobia or ophidiophobia or “social phobia” or “separation anxiety disorder” or “EATING DISORDERS” or “anorexia nervosa” or “binge eating disorder” or bulimia or “SELF-INJURIOUS BEHAVIOR” or “self-destructive behavior” or “self-inflicted wounds” or “self-mutilation” or “self-poisoning” or suicide or “attempted suicide” or suicidality or “suicidal ideation” or “suicide prevention” or “SOMATOFORM DISORDERS” or “body dysmorphic disorder” or hypochondriasis or hysteria or neurasthenia or neurodermatitis or “somatization disorder” or “somatoform pain disorder” or “munchausen syndrome” or “munchausen syndrome by proxy” or (depression near/2 emotion) or "stress and trauma related disorders" or “mental health” or “mental health services” or “community mental health services” or “community counseling” or “community psychiatry”):kw 45016

#23 ("acute stress" or (adjustment next disorder*) or ADNOS or (affective next disorder*) or agoraphobi* or "anorexia nervosa" or anxiety or astheni* or (attachment next disorder*) or (binge next eat*) or binging or (body next dysmorphi*) or bulimi* or (combat next disorder*) or (obsessive next compulsi*) or depression or depressed or depressive or dyssomni* or dyspareunia* or dysphori* or dysthymi* or dystoni* or (eating next disorder*) or EDNOS or (emotional next trauma) or fear or (health next anxiety) or hoarding or hyperactivity or hypochondri* or hysteri* or (medically next unexplained) or malingering or MDD or (common next mental) or (mental* near/2 (health or well*)) or mood or moods or munchausen or MUPS or mutism or neurastheni* or neurotic or neuros* or panic or phobi* or PND or ((post-trauma* or posttrauma*) next stress*) or psychogenic or psychosomatic or PTSD or (self next (injur* or harm or mutilat*)) or psychosexual or (psychological near/3 sexual near/3 dysfunction*) or "social anxiety" or somati* or somatoform or suicid* or parasuicid* or trichotillomani*):ti,ab 196731

#24 (depress* NEAR (employment or employability or unemployment or "return to work" or vocation* or absenteeism or presenteeism or "job security" or "work related" or "work focussed")):ab241

#25 (#1 OR #2 OR #3 OR #4 OR #5 OR #6 OR #7 OR #8 OR #9 OR #10 OR #11 OR #12 OR #13 OR #14 OR #15 OR #16 OR #17 OR #18 OR #19 OR #20 OR #21 OR #22 OR #23 OR #24) 221261

#26 SR-SCIHZ OR HS-SCHIZ 941

#27 (bipolar or cyclothymi* or mania or manic or hypermani* or “rapid cycling” or (conversion next disorder*) or (dissociative next (amnesi* or fugue* or disorder*)) or "borderline state" or catatoni* or "character disorder" or delusion* or (capgras next syndrom*) or (diogenes next syndrom*) or depersonalization or depersonalisation or de-personalization or de-personalisation or (perceptual next disorder*) or (personality next disorder*) or BPD or paranoi* or psychiatr* or psychopathol* or psycho-pathol* or psychotic or psychosis* or psychoses* or psychotic* or schizo* or hebephreni* or “serious mental” or SMI):ti,ab,kw 69764

#28 MeSH descriptor: [Schizophrenia Spectrum and Other Psychotic Disorders] explode all trees 12178

#29 MeSH descriptor: [Personality Disorders] explode all trees 1828

#30 MeSH descriptor: [Bipolar and Related Disorders] explode all trees 3561

#31 (#26 OR #27 OR #28 OR #29 OR #30) 70436

#32 (loneliness or lonely):ti,ab,kw 1395

#33 ((social* or societ* or communit*) near (isolated or isolation)):ti,ab,kw1724

#34 ((alleviat* or ease or manag* or mitigat* or prevent* or overcom* or reduc* or stop*) near/2 (isolation or isolated)):ti,ab,kw 324

#35 ((address* or enhanc* or improv* or increas* or promot* or target*) near/2 (inclusion or inclusivity)):ti,ab,kw 673

#36 (#32 or #33 or #34 or #35) 3739

#37 (#25 and #36) with Cochrane Library publication date Between Jul 2020 and Feb 2024, in Trials916

#38 (#31 and #36) with Cochrane Library publication date Between Jul 2020 and Feb 2024, in Trials173

#39 (“social alienation” or “social inclusion” or “social participation”):ti,ab,kw 1202

#40 (“community involvement” or “social support” or “social network” or “psychosocial environment” or “psychosocial rehabilitation”):ti,kw 7825

#41 (abandonment or alienation):ti,kw 77

#42 ((social or societ*or community) next (confine* or contact or contacts or connect* or inclusion or network* or participation or relations*)):ti,kw 2725

#43 ((social* or societ* or communit*) near/3 (alienat* or discriminat* or excluded or exclusion)):ti,ab,kw 459

#44 ((alleviat* or ease or manag* or mitigat* or prevent* or overcom* or reduc* or stop*) near/2 (exclusion or excluded)):ti,ab,kw 135

#45 ((social* or societ* or communit*) next network*):ti,ab,kw 2626

#46 ((social or societ* or psychosocial) adj support*):ti,ab,kw 21

#47 ("social capital" or (social* next mobil*)):ti,ab,kw 239

#48 (navigator or navigators):ti,kw 338

#49 ((peer or peers) next support*):ti,kw 604

#50 (anti-stigma* or ((intervention* or alleviat* or prevent* or reduc* or stop*) near/2 stigma*)):ti,ab,kw 944

#51 ("social learning theory" or ((social near interaction*) next (counsel* or educat* or intervention* or program* or therap* or train*))):ti,ab,kw 375

#52 (#39 OR #40 OR #41 OR #42 OR #43 OR #44 OR #45 OR #46 OR #47 OR #48 OR #50 OR #51) 13310

#53 (#31 AND #52) with Cochrane Library publication date Between Jul 2020 and Feb 2024, in Trials444

#54 ("social alienation" or "social inclusion" or "social participation"):ti kw 0

#55 ("community involvement" or "social network" or "psychosocial environment" or "psychosocial rehabilitation"):ti,kw 1045

#56 (abandonment or alienation):ti,kw 77

#57 ((chang* or develop* or enhanc* or initiative* or intervention* or program* or mitigat* or address* or improv* or target*) NEAR ((social or societ*or community) next (confine* or contact or contacts or connect* or inclusion or network* or participation or relations*))):ti,ab,kw 1563

#58 ((social* or societ* or communit*) near/3 (alienat* or discriminat* or excluded or exclusion)):ti,ab,kw 459

#59 ((alleviat* or ease or manag* or mitigat* or prevent* or overcom* or reduc* or stop*) near/2 (exclusion or excluded)):ti,ab,kw 135

#60 ((social* or societ* or communit*) next network*):ti,kw 1356

#61 ((social or societ* or psychosocial) next support*):ti 980

#62 (“social capital” or (social* next mobil*)):ti,ab,kw 239

#63 (navigator or navigators):ti,kw 338

#64 ((peer or peers) next support*):ti,kw 604

#65 (anti-stigma* or ((intervention* or alleviat* or prevent* or reduc* or stop*) near/2 stigma*)):ti,ab,kw 944

#66 ("social learning theory" or ((social near interaction*) next (counsel* or educat* or intervention* or program* or therap* or train*))):ti,ab,kw 375

#67 (#54 OR #55 OR #56 OR #57 OR #58 OR #59 OR #60 OR #61 OR #62 OR #63 OR #64 OR #65 OR #66) 6548

#68 (#31 AND #67) with Cochrane Library publication date Between Jul 2020 and Feb 2024, in Trials338

Search Name: CCRT_Barnett_Social Participation SMI

Date Run: 28/02/2024 10:55:56

Comment:

ID Search Hits

#1 SR-DEPRESSN OR HS-DEPRESSN 35371

#2 MeSH descriptor: [Mental Disorders] this term only 5377

#3 MeSH descriptor: [Anxiety Disorders] explode all trees 10069

#4 MeSH descriptor: [Trichotillomania] this term only 96

#5 MeSH descriptor: [Feeding and Eating Disorders] this term only1185

#6 MeSH descriptor: [Anorexia Nervosa] this term only 754

#7 MeSH descriptor: [Binge-Eating Disorder] this term only 482

#8 MeSH descriptor: [Bulimia Nervosa] this term only 362

#9 MeSH descriptor: [Mood Disorders] this term only 1127

#10 MeSH descriptor: [Depressive Disorder] explode all trees 16414

#11 MeSH descriptor: [Premenstrual Dysphoric Disorder] this term only 51

#12 MeSH descriptor: [Reactive Attachment Disorder] this term only 24

#13 MeSH descriptor: [Sexual Dysfunctions, Psychological] this term only 504

#14 MeSH descriptor: [Somatoform Disorders] explode all trees 945

#15 MeSH descriptor: [Munchausen Syndrome] explode all trees 2

#16 MeSH descriptor: [Trauma and Stressor Related Disorders] explode all trees 4508

#17 MeSH descriptor: [Trauma and Stressor Related Disorders] explode all trees 4508

#18 MeSH descriptor: [Impulsive Behavior] explode all trees 1672

#19 MeSH descriptor: [Hoarding] this term only 7

#20 MeSH descriptor: [Self-Injurious Behavior] explode all trees 2427

#21 ("anorexia nervosa" or "binge eating disorder" or bulimia or "eating disorder" or automutilation or "suicidal behaviour" or "self poisoning" or "suicidal ideation" or suicide or "suicide attempt" or depression or "agitated depression" or "atypical depression" or "chronic depression" or "depressive psychosis" or dysphoria or dysthymia or "endogenous depression" or involutional or "late life depression" or "major depression" or melancholia or "minor depression" or "mourning syndrome" or "organic depression" or "perinatal depression" or "antenatal depression" or "postnatal depression" or "post-stroke depression" or "postoperative depression" or "premenstrual dysphoric disorder" or "reactive depression" or "recurrent brief depression" or "seasonal affective disorder" or "treatment resistant depression" or neurosis or "affective neurosis" or "anxiety neurosis" or dysthymia or hysteria or neurasthenia or psychasthenia or "adjustment disorder" or "anxiety disorder" or "acute stress disorder" or "generalized anxiety disorder" or panic or "posttraumatic stress disorder" or "separation anxiety" or "obsessive compulsive disorder" or compulsion or obsession or phobia or agoraphobia or claustrophobia or neophobia or "social phobia" or "somatoform disorder" or "body dysmorphic disorder" or "conversion disorder" or "delusional pregnancy" or hypochondriasis or "masked depression" or "psychogenic pain" or somatization or "mood disorder" or "affective neurosis" or "affective psychosis" or "blunted affect" or "major affective disorder" or "minor affective disorder" or "munchausen syndrome by proxy" or "munchausen syndrome" or "psychosexual disorder" or kleptomania or "trichotillomania" or "emotional disorder"):kw 63877

#22 ("MENTAL DISORDERS" or anhedonia or neurosis or "ACUTE STRESS DISORDER" or "adjustment disorders" or "attachment disorders" or "disinhibited social engagement disorder" or "posttraumatic stress disorder" or "complex ptsd" or

desnos or “acute stress disorder” or “post-traumatic stress” or “traumatic neurosis” or “emotional trauma” or “AFFECTIVE DISORDERS” or “disruptive mood dysregulation disorder” or “dysthymic disorder” or “seasonal affective disorder” or “major depression” or “anaclitic depression” or “dysthymic disorder” or “endogenous depression” or “late life depression” or “postpartum depression” or “reactive depression” or “recurrent depression” or “treatment resistant depression” or “premenstrual dysphoric disorder” or “ANXIETY DISORDERS” or “generalized anxiety disorder” or “obsessive compulsive disorder” or “panic attack” or “panic disorder” or phobias or trichotillomania or acrophobia or agoraphobia or claustrophobia or ophidiophobia or “social phobia” or “separation anxiety disorder” or “EATING DISORDERS” or “anorexia nervosa” or “binge eating disorder” or bulimia or “SELF-INJURIOUS BEHAVIOR” or “self-destructive behavior” or “self-inflicted wounds” or “self-mutilation” or “self-poisoning” or suicide or “attempted suicide” or suicidality or “suicidal ideation” or “suicide prevention” or “SOMATOFORM DISORDERS” or “body dysmorphic disorder” or hypochondriasis or hysteria or neurasthenia or neurodermatitis or “somatization disorder” or “somatoform pain disorder” or “munchausen syndrome” or “munchausen syndrome by proxy” or (depression near/2 emotion) or "stress and trauma related disorders" or “mental health” or “mental health services” or “community mental health services” or “community counseling” or “community psychiatry”):kw 45016

#23 ("acute stress" or (adjustment next disorder*) or ADNOS or (affective next disorder*) or agoraphobi* or "anorexia nervosa" or anxiety or astheni* or (attachment next disorder*) or (binge next eat*) or binging or (body next dysmorphi*) or bulimi* or (combat next disorder*) or (obsessive next compulsi*) or depression or depressed or depressive or dyssomni* or dyspareunia* or dysphori* or dysthymi* or dystoni* or (eating next disorder*) or EDNOS or (emotional next trauma) or fear or (health next anxiety) or hoarding or hyperactivity or hypochondri* or hysteri* or (medically next unexplained) or malingering or MDD or (common next mental) or (mental* near/2 (health or well*)) or mood or moods or munchausen or MUPS or mutism or neurastheni* or neurotic or neuros* or panic or phobi* or PND or ((post-trauma* or posttrauma*) next stress*) or psychogenic or psychosomatic or PTSD or (self next (injur* or harm or mutilat*)) or psychosexual or (psychological near/3 sexual near/3 dysfunction*) or "social anxiety" or somati* or somatoform or suicid* or parasuicid* or trichotillomani*):ti,ab 196731

#24 (depress* NEAR (employment or employability or unemployment or "return to work" or vocation* or absenteeism or presenteeism or "job security" or "work related" or "work focussed")):ab241

#25 (#1 OR #2 OR #3 OR #4 OR #5 OR #6 OR #7 OR #8 OR #9 OR #10 OR #11 OR #12 OR #13 OR #14 OR #15 OR #16 OR #17 OR #18 OR #19 OR #20 OR #21 OR #22 OR #23 OR #24) 221261

#26 SR-SCIHZ OR HS-SCHIZ 941

#27 (bipolar or cyclothymi* or mania or manic or hypermani* or “rapid cycling” or (conversion next disorder*) or (dissociative next (amnesi* or fugue* or disorder*)) or "borderline state" or catatoni* or "character disorder" or delusion* or (capgras next syndrom*) or (diogenes next syndrom*) or depersonalization or depersonalisation or de-personalization or de-personalisation or (perceptual next disorder*) or (personality next disorder*) or BPD or paranoi* or psychiatr* or psychopathol* or psycho-pathol* or psychotic or psychosis* or psychoses* or psychotic* or schizo* or hebephreni* or “serious mental” or SMI):ti,ab,kw 69764

#28 MeSH descriptor: [Schizophrenia Spectrum and Other Psychotic Disorders] explode all trees 12178

#29 MeSH descriptor: [Personality Disorders] explode all trees 1828

#30 MeSH descriptor: [Bipolar and Related Disorders] explode all trees 3561

#31 (#26 OR #27 OR #28 OR #29 OR #30) 70436

#32 (loneliness or lonely):ti,ab,kw 1395

#33 ((social* or societ* or communit*) near (isolated or isolation)):ti,ab,kw1724

#34 ((alleviat* or ease or manag* or mitigat* or prevent* or overcom* or reduc* or stop*) near/2 (isolation or isolated)):ti,ab,kw 324

#35 ((address* or enhanc* or improv* or increas* or promot* or target*) near/2 (inclusion or inclusivity)):ti,ab,kw 673

#36 (#32 or #33 or #34 or #35) 3739

#37 (#25 and #36) with Cochrane Library publication date Between Jul 2020 and Feb 2024, in Trials916

#38 (#31 and #36) with Cochrane Library publication date Between Jul 2020 and Feb 2024, in Trials173

#39 (“social alienation” or “social inclusion” or “social participation”):ti,ab,kw 1202

#40 (“community involvement” or “social support” or “social network” or “psychosocial environment” or “psychosocial rehabilitation”):ti,kw 7825

#41 (abandonment or alienation):ti,kw 77

#42 ((social or societ*or community) next (confine* or contact or contacts or connect* or inclusion or network* or participation or relations*)):ti,kw 2725

#43 ((social* or societ* or communit*) near/3 (alienat* or discriminat* or excluded or exclusion)):ti,ab,kw 459

#44 ((alleviat* or ease or manag* or mitigat* or prevent* or overcom* or reduc* or stop*) near/2 (exclusion or excluded)):ti,ab,kw 135

#45 ((social* or societ* or communit*) next network*):ti,ab,kw 2626

#46 ((social or societ* or psychosocial) adj support*):ti,ab,kw 21

#47 (“social capital” or (social* next mobil*)):ti,ab,kw 239

#48 (navigator or navigators):ti,kw 338

#49 ((peer or peers) next support*):ti,kw 604

#50 (anti-stigma* or ((intervention* or alleviat* or prevent* or reduc* or stop*) near/2 stigma*)):ti,ab,kw 944

#51 ("social learning theory" or ((social near interaction*) next (counsel* or educat* or intervention* or program* or therap* or train*))):ti,ab,kw 375

#52 (#39 OR #40 OR #41 OR #42 OR #43 OR #44 OR #45 OR #46 OR #47 OR #48 OR #50 OR #51) 13310

#53 (#31 AND #52) with Cochrane Library publication date Between Jul 2020 and Feb 2024, in Trials444

Supplementary Materials IV:

Supplementary Table 2. Glossary of key terms

Acronym or term	Description
**ACT**	Assertive Community Treatment (ACT) is an intensive and integrated approach to community based mental health care for people living with severe mental illness.
**ASHA**	The ASHA Project aims to evaluate the impact of depression treatment and economic strengthening intervention for low-income women in rural Bangladesh. ASHA means ‘Hope’ in Bangladeshi.
**Banyan**	The ‘Banyan’ recovery model is a supported housing initiative which aids service users with the transition from hospital-based settings to community-based independent living facilities.
**CaFI**	The Culturally-adapted Family Intervention (CAFI) aims to support individuals from Black African or Caribbean heritage living with schizophrenia, and their respective family members and/or key workers.
**CBSST**	Cognitive Behavioural and Social Skills Training (CBSST) combines elements of Cognitive Behavioural Therapy (CBT) with Social Skills Training (SST) and targets functional impairments in people living with psychosis.
**Critical Time Intervention**	The Critical Time Intervention (CTI) is a time-limited intervention that aims to provide support to people living with severe mental illness during vulnerable periods of transition.
**FACT**	Forensic Assertive Community Treatment (FACT), drawn from the ACT model, is a community-based intervention strategy for people who are living with a severe mental illness and are involved in the criminal justice system.
**FFT-HPI**	The Family-Focused Treatment Health Promoting Intervention (FFT-HPI) works with caregivers of people living with mental ill-health to enhance skills surrounding illness-management and self-care.
**HOPE**	The ‘Helping to Overcome PTSD through Empowerment’ (HOPE) intervention was developed specifically for women who had been violently assaulted by a partner and were accessing shelter.
**Housing First**	Housing First is a model of care which aims to provide stable housing to people who have experienced homelessness and mental ill-health. The support provided is intensive, holistic and open-ended.
**HUGS**	‘HUGS’ is a group-based mother-infant interaction intervention, which may be preceded by a CBT session for postnatal depression.
**Integrated Dual Disorders Treatment**	The Integrated Dual Disorders Treatment (IDDT) intervention aims to improve the quality of life of people living with severe mental illness and comorbid substance use disorder, by providing integrated and evidence-based support for both mental health care and substance abuse.
**MISSION-VET**	Maintaining Independence and Sobriety through Systems Integration, Outreach and Networking-Veterans Edition (MISSION-VET) provides case management, psychoeducational treatment, and vocational support to homeless or formerly homeless veterans with co-occurring psychiatric and substance use disorders.
**mTC**	Modified Therapeutic Communities (mTC) draw on the key principles of Therapeutic Communities to provide more integrated care for people living with both severe mental illness and co-occurring substance use disorders.

**PFR**	Promoting First Relationships (PFR) was initially developed as an intervention for toddlers in state dependency/child welfare and has been adapted to support low-income new mothers living with mental ill-health.
**Songs from Home**	'Songs from Home' is a songwriting programme intervention which is designed to address loneliness in new mothers.
